# The assessment of presence and performance in an AR environment for motor imitation learning: A case-study on violinists

**DOI:** 10.1016/j.chb.2023.107810

**Published:** 2023-09

**Authors:** Adriaan Campo, Aleksandra Michałko, Bavo Van Kerrebroeck, Boris Stajic, Maja Pokric, Marc Leman

**Affiliations:** a Department of Art, Music and Theatre Sciences, Faculty of Arts and Philosophy, Institute for Psychoacoustics and Electronic Music (IPEM), Ghent University, Miriam Makebaplein 1, B-9000, Gent België, Belgium; b ARVRtech, Antona Čehova 1, Novi Sad, Serbia

**Keywords:** Presence, Augmented reality, Motor learning, Stereoscopic vision, Skill transfer

## Abstract

The acquisition of advanced gestures is a challenge in various domains of proficient sensorimotor performance. For example, orchestral violinists must move in sync with the lead violinist's gestures. To help train these gestures, an educational music play-back system was developed using a HoloLens 2 simulated AR environment and an avatar representation of the lead violinist. This study aimed to investigate the impact of using a 2D or 3D representation of the lead violinist's avatar on students' learning experience in the AR environment.

To assess the learning outcome, the study employed a longitudinal experiment design, in which eleven participants practiced two pieces of music in four trials, evenly spaced over a month. Participants were asked to mimic the avatar's gestures as closely as possible when it came to using the bow, including bowing, articulations, and dynamics. The study compared the similarities between the avatar's gestures and those of the participants at the biomechanical level, using motion capture measurements, as well as the smoothness of the participants' movements. Additionally, presence and perceived difficulty were assessed using questionnaires.

The results suggest that using a 3D representation of the avatar leads to better gesture resemblance and a higher experience of presence compared to a 2D representation. The 2D representation, however, showed a learning effect, but this was not observed in the 3D condition. The findings suggest that the 3D condition benefits from stereoscopic information that enhances spatial cognition, making it more effective in relation to sensorimotor performance. Overall, the 3D condition had a greater impact on performance than on learning.

This work concludes with recommendations for future efforts directed towards AR-based advanced gesture training to address the challenges related to measurement methodology and participants' feedback on the AR application.

## Highlights

• 11 amateur violinists practiced 2 different pieces 4 times over the course of 1 month with a 2D and 3D violin teacher avatar.
• Performance and learning were assessed using motion capture data, as well as presence.
• Presence and performance were higher in the 3D condition.
• Learning was only observed in the 2D condition.
• Stereoscopy rather than presence, influences advanced sensorimotor imitation learning.

## 1 Introduction

The teaching of refined gestures presents an educational challenge in several domains of skilled sensorimotor performance. With the advent of augmented reality (AR) technologies such as the HoloLens, it becomes possible to practice advanced gestures with a virtual teacher in an AR environment. However, to assess the extent to which immersion, realism and presence influence the effectiveness of a learning experience, it is necessary to set up controlled experiments of sufficient ecological value.

In this study, we aimed to investigate the effectiveness of an AR play-back system for learning advanced gestures. Specifically, we simulated an orchestra sectional rehearsal scenario, where participating violinists were required to mimic a virtual section leader, referred to as the avatar, as closely as possible. The study compared the gesture similarity of participants in different conditions (2D and 3D rendering of the avatar) and over different trials, with the hypothesis that different conditions would induce varying levels of presence in the participants. To examine the relationship between presence and students' musical performance and learning, we employed motion capture and biomedical metrics to evaluate the quality of musical performance. Furthermore, questionnaires were utilized to assess students' perceived level of presence and learning outcomes. Ultimately, the objective of this study is to investigate the impact of representing lead violinists' avatar in 3D on students' musical performance and learning.

The use of play-back systems can be an incredibly valuable tool for music education and practice, especially for violin players. Imitating bow use is especially important in a student-teacher context, as the details of 3D motion are critical for mastering the bow and achieving sound, musical interpretation, virtuosity, and other performance aspects. In addition, the coordinated use of the bow is essential in an orchestral context, where players must move in sync to produce high-quality sound, rhythm clarity, articulation, phrasing, and visual effects.

The achievement of gestural similarity with the section leader, or avatar, is an essential aspect of orchestral practice and explicit orchestral hierarchy (Marotto et al., 2007). Gestural similarity between the section and its leader is especially important in orchestral string sections, as it is a prerequisite for a cohesive string sound (2023Improve Your Orchestral Playing). Therefore, the challenge of mastering the bow in an educational context has significant implications not only for the individual player but also for the overall performance of the orchestra.

In this context, play-back systems relying on 2D rendering lack the stereoscopic information, which is known to enhance spatial awareness (Kober et al., 2012), and accurate 3D motor coordination (Levac et al., 2019). In a more general context, a realistic 3D rendering of a virtual teacher or fellow musician, has the additional benefit that it can enhance the level of presence (Bowman & McMahan, 2007), while a higher realism of humanoid renderings in the environment induces a higher degree of social presence (Yoon et al., 2019). Social presence is important in education (Garrison et al., 1999; Hostetter, 2013), and music ensemble playing (Gaggioli et al., 2017). Finally, a 3D rendering allows for a more realistic playing experience, as players can decide where they position themselves relative to the avatar, i.e., in front of him like in a partial rehearsal, a lesson or a chamber music setting, or behind him, like in the orchestra.

### 1.1 AR and VR

Augmented reality (AR) allows for an interactive experience of a real-world environment in which objects in the real world are enhanced by computer-generated perceptual information, sometimes across multiple sensory modalities (Wellner et al., 1993). AR includes a combination of real and virtual worlds, real-time interaction, and accurate 3D registration of virtual and real objects. Its overlapping sensory information can be constructive (i.e., adding to the natural environment), or destructive (i.e., masking the natural environment) (Furht, 2006). Accordingly, AR is situated on the Milgram Virtuality Continuum (Milgram & Kishino, 1994), but unlike virtual reality (VR), which requires a complete immersion in a simulated environment, AR merely changes one's ongoing perception of the real-world environment. The benefits of AR in music education are explained in paragraph 1.5.

### 1.2 Immersion and presence

Immersion refers to the technological ability of a medium to create realistic experiences that can transport individuals away from their physical surroundings (Slater & Wilbur, 1997). Immersion can be quantified through the technological features of a medium (Cummings & Bailenson, 2016). In contrast, presence is the subjective experience of actually being present in the virtual environment created by the medium (Slater & Wilbur, 1997). There are three distinct subcategories of presence: self-presence, physical presence, and social presence (Lee, 2004).

Physical presence can be defined as the extent to which an individual feels present in the mediated environment rather than in the physical environment (Lee, 2004). This dimension of presence is strongly related to the user's experience of the environmental and spatial properties of the mediated environment. The more intense the experience of physical presence, the less aware individuals are of the technological mediation of their experiences (Lee, 2004).

In contrast, self-presence is the extent to which an individual's virtual self is experienced as their actual self (Lee, 2004).Unlike physical presence, self-presence is not related to the vividness of one's surroundings but rather the degree of identification and connection felt to their virtual body, emotions, or identity (Lee, 2004). This type of presence is of lesser importance for AR (Lee, 2004).

Finally, social presence refers to the extent to which an individual attributes mental states, intelligence, and intentions to a virtual human that might not have them. It was first defined as the salience of the interactants and their interpersonal relationship during a mediated conversation (Short et al., 1976). It refers to the sense of being with another (Biocca et al., 2003) and is dependent on the ease with which one perceives to have the access to the intelligence, intentions, and sensory impressions of another (Biocca, 2006). Social presence is distinct from both physical presence and self-presence as it requires a co-present entity that appears sentient.

### 1.3 Social presence and education

The traditional and widely accepted form of education is face-to-face (FTF) instruction, which is considered the gold standard. In FTF settings, researchers evaluate communication using constructs such as teacher immediacy and intimacy, which are closely related. Intimacy refers to the sense of connectedness experienced by communicators during an interaction, while immediacy relates to the psychological distance between them. Verbal and nonverbal cues, such as facial expressions, vocal cues, gestures, and physical appearance, can determine both intimacy and immediacy (Gunawardena & Zittle, 1997).

In computer-mediated education, the communication process is mediated by technology, creating social climates that differ from those of traditional classrooms. Even two-way interactive technology that allows the transmission of verbal and nonverbal cues can lead to different interaction patterns from those in an FTF context (Short et al., 1976). Thus, the concept of social presence is particularly relevant in this context. It shares similarities with intimacy and immediacy but also accounts for the intermediating variable of media (Gunawardena & Gunawardena, 1995). As some media are better at delivering the verbal and nonverbal cues crucial for intimacy and immediacy, social presence can be considered a function of the medium (Kreijns et al., 2007; Short et al., 1976), among other parameters (Gunawardena & Zittle, 1997; Petersen et al., 2021; Walther, 1992).

Social presence is crucial in computer-mediated education (Garrison et al., 1999; Gunawardena & Zittle, 1997; Richardson & Swan, 2003; Rourke et al., 1999), as increasing social presence can enhance learning (Garrison et al., 1999; Hauber et al., 2005; Hostetter, 2013), student satisfaction (Tu & Tu, 2002), and emotional connections (Aragon, 2003, pp. 57–68) in computer-mediated learning environments, as well as in music ensemble playing (Gaggioli et al., 2017), or collaboration with virtual colleagues (Fancourt & Steptoe, 2019; Van Kerrebroeck et al., 2021).

The connection between presence and learning is a subject of significant interest in XR research (Richey, 2018). In the VR domain, there is generally a positive correlation between presence and learning outcomes (Krassmann et al., 2022). However, the available literature reports contradictory findings (Weidner et al., 2023). Some studies propose that higher presence results in better learning (Ai-Lim Lee et al., 2010; Alhalabi, 2016; Cadet & Chainay, 2020; Kozhevnikov et al., 2013; Rasheed et al., 2015; Ray & Deb, 2017; Stevens & Kincaid, 2015), while others produce inconclusive or even negative results (Allmendinger, 2010; Buttussi & Chittaro, 2018; George et al., 2018; Makransky et al., 2019a; Moreno et al., 2001; Ochs & Sonderegger, 2022; Pears & Konstantinidis, 2022).

Although it is not yet clear how these findings apply to the AR domain, some parallels can be drawn (Weidner et al., 2023). Several studies suggest a positive relationship between presence and learning in the AR domain (Kye & Kim, 2008). Specifically, it was discovered that social presence relates to better collaboration (Yoon et al., 2019), usability (Wang et al., 2020), confidence (Cao et al., 2020), and learning (Chen & Wang, 2018). Additionally, high levels of immersion have a positive influence on the extent of learning in an AR experience (Bressler & Bodzin, 2013; Dunleavy et al., 2009; Georgiou & Kyza, 2017, 2018; Lin et al., 2020; Rossano et al., 2020). For example, they improve learning outcomes (Uriarte-Portillo et al., 2022), increase engagement (Rebollo et al., 2022; Wankel & Blessinger, 2012), and promote enjoyment (Koh et al., 2017).

These studies reinforce the idea that presence impacts students' learning outcomes during AR educational activities and shapes their learning outcomes (Cheng & Tsai, 2013). However, the literature is too diverse to draw definitive conclusions on this relationship at this time (Kaplan et al., 2021).

### 1.4 Avatar representation and learning

Verbal and non-verbal cues are crucial to understanding communication (Nguyen & Canny, 2009), so it is logical that avatar representation has a significant impact on presence and social presence (Cai & Tanaka, 2019; Weidner et al., 2023). For example, literature suggests that realistic-looking avatars increase co-presence (Casanueva & Blake, 2001), and social presence (Pakanen et al., 2022). Several other parameters of avatar representation influence social presence, such as which body parts are displayed (Heidicker et al., 2017; Yoon et al., 2019), gender (Makransky et al., 2019b), interactivity (Oh et al., 2018), movement fidelity (Heidicker et al., 2017; Roth et al., 2016), mouth movement (Yoshimura & Borst, 2021), and more (Weidner et al., 2023).

Spatiality is another important factor. 3D avatars, for example, enable negotiating the relative position between teacher and student (Sellen, 1992), which is relevant since social norms about proxemics seem to remain consistent in virtual and real-world environments (Bailenson et al., 2001). Additionally, 3D avatars help in gaze awareness (Steptoe et al., 2010), which is a critical element of (non-)verbal communication (Argyle et al., 1994; Kendon, 1967). Finally, a 3D environment enables better interactivity through spatial cues, such as depth, resolution, and field of view (Pan & Steed, 2016; Steptoe et al., 2010).

Given the significance of non-verbal communication (Nguyen & Canny, 2009), interaction (Fortin & Dholakia, 2005; Skalski & Tamborini, 2007), and spatiality (Ahn et al., 2014; Kim et al., 2012a; Muhlbach et al., 1995; Takatalo et al., 2011) in social presence, it is unsurprising that a 3D avatar enhances the experience of social presence (Fancourt & Steptoe, 2019; Hauber et al., 2005, 2006; Hepperle et al., 2020; Kim et al., 2012b; Susindar et al., 2019; Van Kerrebroeck et al., 2021), and, as a result, the learning environment (Downey et al., 2012; Murcia-López & Steed, 2016; Sebastian et al., 2019), when compared to a 2D avatar.

However, the additional spatial dimension may come at a cost, such as higher mental load, increased confusion, more task distraction, or reduced task performance overall (Hauber et al., 2006). Therefore, it is necessary to investigate the relationship between stereoscopy, social presence, and learning further (Oh et al., 2018).

### 1.5 AR in music education

The potential of AR in education has been explored in early childhood education (Han et al., 2015), K-12 education and higher education (Moro et al., 2021; Saltan & Arslan, 2017). Several reports and studies, including the influential Horizon reports, proposed AR technology as a key educational technology for the foreseeable future (Johnson et al., 2011, 2014), driving a transformation in education (Johnson et al., 2014) and bringing a potential improvement in learning outcomes (Reilly & Dede, 2019). AR can mimic experiences not available in real life (Wojciechowski & Cellary, 2013; Wu et al., 2013); it helps to save time and space (Aziz et al., 2012; Li, 2010); it has the power to increase student participation (Wojciechowski & Cellary, 2013; Yoon et al., 2012), motivation, attention (Aziz et al., 2012; Sumadio & Rambli, 2010) and cooperation (O'Shea, 2011; Yuen et al., 2011); it creates the possibility to learn through entertainment (Yoon et al., 2012); among other pedagogical benefits (Saltan & Arslan, 2017). Because of its unique capabilities, AR supports constructive learning, learning by doing and authentic learning, which serve to make students active in the learning environment (Shelton & Stevens, 2004, p. 634; Wojciechowski & Cellary, 2013; Yilmaz & Goktas, 2017; Yuen et al., 2011). In higher education, AR has mainly been assessed in the field of science and engineering (Azuma, 1997; Yuen et al., 2011), with positive results overall (Bacca et al., 2014). However, while AR research is prominent in the arts and humanities (Bacca et al., 2014), music education is a rather rare application of AR, and AR applications for instrument learning have only appeared recently. Most AR tools focus on learning piano or guitar (e.g. (Trujano et al., 2018), or (Martin-Gutierrez et al., 2020)) by providing information on timing and rhythm or providing feedback on where to place a player's fingers to produce a particular tone.

The unique advantage of AR in the context of the present study is that the violinist can rehearse synchronized playing, even with a precise measurement of accuracy. Although FTF teaching remains the gold standard for violin lessons, AR can be a valuable alternative when physical presence is not possible, as indicated in other fields (Hale et al., 2022). This became evident during the COVID pandemic, as reiterated in the experiences of the participants in this study. Another potential benefit is that an AR application could provide a completely new way to teach music, as it offers the benefits of digital tools, creates a degree of social presence, presents stereoscopic information, and has the potential for gamification. Moreover, it can provide a realistic simulated experience of playing music with others. Finally, the technology-laden device serves as a valuable tool to study human behavior, also in educational science (Van Kerrebroeck et al., 2021).

### 1.6 HoloLens in education

Of particular interest to many educators is the Microsoft HoloLens, a recently introduced frontal display that uses holographic technology to generate enhanced images. These holograms can be used through hand gestures or voice commands. The HoloLens falls within the AR portion of the Milgram Virtuality Continuum (Milgram & Kishino, 1994), augmenting real-world elements with persistent and interactive visuals. In particular, the HoloLens enables binocular depth cues, which offers the possibility to reproduce virtual 3D models. The detailed 3D models presented in the HoloLens, as well as its hands-free nature and the ability to manipulate holographic images in real space, are some of the prominent benefits of using this technology. Additionally, the HoloLens has demonstrated the potential to significantly improve retention, spatial awareness, and enjoyable teaching (Brun et al., 2019; Hackett & Proctor, 2018; Mitsuno et al., 2019). Finally, this technology provides stereoscopic information, which is lacking in more conventional (a)synchronous remote instruction methods. Since, in analogy with VR, the quality of an AR experience is measured by presence and immersion, it is important to improve our understanding of the understudied relationship between presence, learning and performance in AR.

### 1.7 Training sophisticated music gestures through AR

Traditional music education is usually based on a dyadic teacher-student relationship, where there is often a considerable lag between the motoric performance of the student and feedback of the teacher. Moreover, the teaching of the biomechanical skills required for an accurate and safe performance are often limited by subjective and vague perception and based on oral transmission of content between the teacher and the students (Brandfonbrener, 2003). It therefore seems reasonable to assume that more quantitative methods that have already been tested and are useful in other contexts, such as in sports medicine, can be applied in and be useful for the understanding and teaching of sophisticated gestures for musical performance (Brandfonbrener, 2003). The background knowledge that can clarify motor skills required in musical performance includes motor learning theory and technology-based systems for analysis, monitoring, and evaluation of learning efficiency (Engelhorn, 1983). In motor learning theory, three elements are presented as essential for success: the characterization of the gestures to be acquired, the transfer of gestures between different systems and the acquisition of gestures without injury. Acquiring the characterizing gestures requires scientific analysis to identify motor patterns, such as the coordination of neural and musculo-skeletal systems (Engelhorn, 1983). In addition, the motor behavior of professional players can be used as a reference model to facilitate understanding of the essential gestures to be imparted to music students. By focusing on specific motor behaviors, students can efficiently and effectively incorporate the gestures that suit their technique. Following these findings, an emerging literature is increasingly interested in exploring how whole-body and movement analysis technologies can improve musical performance and learning outcomes, while minimizing the risk of injury (Visentin et al., 2008). On the other hand, several attempts have been made in the literature to assess violin playing quality in a quantitative manner. The rationale was either to provide meaningful feedback during playing (Blanco et al., 2021; Larkin et al., 2008; Van Der Linden et al., 2011), to track the learning process (Konczak et al., 2009; Young & Lippman, 2007), to understand the physics of violin playing (Askenfelt, 1986, 1989; Serafin & Young, 2003; Visentin & Shan, 2003), or simply for the sake of science and technology (Hodgson, 1935; Vamvakousis et al., 2018). However, a metric able to quantify violin playing in a reliable and reproducible way has not been put forward.

### 1.8 Metrics

#### 1.8.1 Presence

Presence in the AR environment is routinely assessed using presence questionnaires. In this work, the Witmer Presence Questionnaire (WPQ) is used, as it is a well-established presence questionnaire, allowing comparison with a wide range of literature (Witmer & Singer, 1998). However, like most available presence assessment tools, this questionnaire is in essence developed for VR environments.

The Makransky Presence Questionnaire (MPQ), also used in this study, allows for probing presence across a wider range of the Milgram Continuum (Makransky et al., 2017). In addition, the MPQ distinguishes between telepresence, social presence, and physical presence. We use the social subset (MPQS) and physical subset (MPQP), but omit the self-presence subset, as it is rather suited for more immersive applications (Lee, 2004).

#### 1.8.2 Biomechanical metrics

In the literature, several biomechanical parameters have been proposed to assess the skill performance of a violinist quantitatively:

- The comparative use of wrist, elbow, and shoulder angles (Konczak et al., 2009)
- Posture (Ackermann & Adams, 2004)
- Sound volume and sound quality (Schoonderwaldt, 2009)
- The Variation in General Movement Patterns (Konczak et al., 2009)
- Jerk and smoothness of movements (also in string crossings) (Ancillao et al., 2017; Gonzalez-Sanchez et al., 2019)

Violinists’ posture and their comparative use of joints show an evolution due to anatomical and artistic development, which can take years (Konczak et al., 2009), posing a challenge for a follow-up study in a shorter time frame. In addition, there is a large natural variation in anatomical parameters, and playing styles within the human population (Shan & Visentin, 2003), making quantitative assessment difficult. While some effort has been made to automatically evaluate sound quality (Blanco et al., 2021), assessing quality remains a challenging domain in sophisticated classical music pieces. In addition, individual sound quality tends to evolve over a long period of time, and is typically practiced and improved during solo practice, rather than group practice.

In the present study, we focus on the overall variation in movement patterns, of the follower (the participant) relative to the (section) leader. The task of a violin section is to move and sound as one unit by coordinated use of the bow. Besides visual aspects, a section's collective bow use determines musical aspects such as loudness, dynamics, accents, color, timbre, rhythm, articulation, phrasing, and intensity. The use of the bow, relative to the violin, was captured with MoCap in both leader and follower, and synchronized, allowing comparison of the dyad. To compare motion curves, a distance measurement is a common approach (Goodallt, 1991). Both bow movements relative to the frog and the bridge are important for sound quality (Schoonderwaldt, 2009). To compensate for any offsets between leader and follower, the 2D Procrustes distance (PD) was calculated as a metric (Goodallt, 1991). The smoothness of movements, on the other hand, is often associated with the skill level (Balasubramanian et al., 2015) and learning effort (Harris & Wolpert, 1998) in motor skill performance, also in violin playing (Ancillao et al., 2017; Gonzalez-Sanchez et al., 2019). As a metric for smoothness, we chose the SPARC index (dSI), as it is considered the state-of-the-art for this purpose (Balasubramanian et al., 2015). Consequently, we define musical performance quality as a function of the skill performance parameters, being gesture similarity between participant and avatar (PD) and the difference in movement smoothness (dSI) between participant and avatar.

#### 1.8.3 Interplay between metrics and conditions

The complexity of a task can be described in terms of the redundancy in execution and task (Levac et al., 2019). Although violin playing is a complex motor task, redundancy in execution is high at the motor level, as execution depends heavily on participants' skill, anatomy, and the music itself (Shan & Visentin, 2003). In contrast, the redundancy in task is low, as participants are asked to imitate the avatar's movements as good as possible (Konczak et al., 2009). Due to that low redundancy, play-back systems relying on 3D rendering can potentially facilitate exact movement imitation in the 3D condition, as assessed by the PD metric. The reason is that 3D rendering provides stereoscopic information, which enhances spatial awareness (Kober et al., 2012), and accurate 3D motor coordination (Levac et al., 2019) as opposed to 2D rendering (Gerig et al., 2018). In addition, it has been suggested that variability in a motor learning task is an important factor to assess motor skill performance and learning (Levac et al., 2019), and variability of movement is a strong indicator of instrument skill level (Konczak et al., 2009), while decrease in variability is an indicator of motor learning (Konczak et al., 2009; Levac et al., 2019). In this regard, training with a 2D or 3D rendering can induce a decrease in PD over time, which can be considered a learning effect (Levac et al., 2019). In a more general context, a realistic 3D rendering of a virtual teacher or fellow musician, allows participants to move freely around the 3D rendering, and position themselves at will during the practice time, increasing ecological validity. Moreover, a 3D rendering, compared to a 2D rendering, can enhance the experience of (social) presence, learning and musical performance quality, as discussed above. Finally, we assess the smoothness of movements, using dSI. Movement smoothness is believed to be related to attention level (Kahol et al., 2008), nervousness (Kolakowska, 2013), and other behavioral factors (Kal et al., 2013), suggesting that there is an interplay with (social) presence. We assume that movement smoothness is related to the PD, as it relates to skill level (Balasubramanian et al., 2015) and learning effort (Harris & Wolpert, 1998), also in violin playing (Ancillao et al., 2017; Gonzalez-Sanchez et al., 2019). In summary, in the context of this work, we define the gradual improvement in musical performance quality over time as “learning”. Accordingly, we anticipate observing a decrease in PD and an increase in dSI as the participants learn during the experiment.

**Fig. 1:**
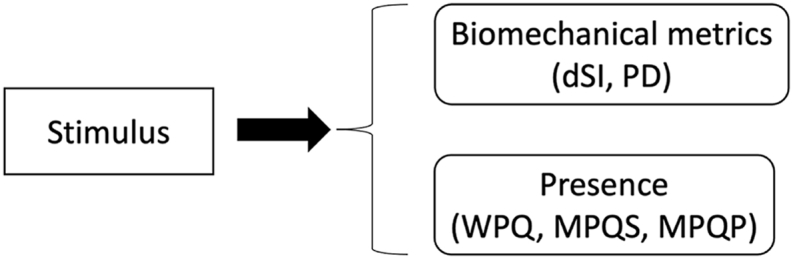
The presumed interplay between presence (MPQS, MPQP, WPQ), and biomechanical metrics (dSI, PD), and experimental conditions (2D/3D rendering).

The interaction between conditions and biomechanical metrics and presence is summarized in Fig. 1.

### 1.9 Experimental design and hypotheses

An educational music play-back system was developed, using a HoloLens 2 simulated AR environment and avatar representation of the principal violinist. The present study compares a 2D and a 3D presentation of the avatar, and it examines the learning and musical performance quality of the participants in the 2 conditions in a within-subjects and longitudinal experiment design, using both biomechanical metrics and presence. The biomechanical markers were newly developed for this study. Eleven participants trained two musical pieces in the 2D, or 3D condition once a week for one month, with a training time of 20 min per piece per week. The pieces and respective conditions remained the same throughout the experiment. After each 15-min training session, the participants performed the full piece synchronized with the avatar of which the data was used for quantitative analysis. The participant's task was to mimic the avatar's movements as closely as possible. The hypotheses of this study are:

1. Violin students will show better musical performance quality in the 3D condition compared to the 2D condition:
  1.1. The similarity between the bow movement of virtual teachers and students is greater.
  1.2. Smoothness of movement is higher.
2. The learning effect on violin playing will be higher in the 3D condition than in the 2D condition.
3. The 3D condition will induce a higher level of presence compared to the 2D condition:
  3.1. The induced level of “physical presence” will be higher.
  3.2. The induced level of “social presence” will be higher.
4. The level of presence in AR influences violin students' musical performance quality.

## 2 Materials and methods

### 2.1 Ethics

Ethics approval was obtained from the Ghent University Ethics committee (ref. 2021–16).

### 2.2 Experimental protocol

Eleven participants rehearsed two orchestral fragments with a virtual audio-visual representation of a concertmaster in an AR environment. Violin players from each violin section were randomly divided into two groups: Group 1: Rehearsing with a 3D avatar on the first fragment and 2D projection of the avatar on the second fragment (3D/2D), and Group 2: Rehearsing with a 2D projection of the avatar on the first fragment and a 3D avatar on the second fragment (2D/3D) (see Table 1). The violinists participated in 4 trials (one trial per week) and condition (2D or 3D) remained the same for each fragment throughout the experiment.

**Table 1:** Overview of participants, their respective violin section and stimulus.

participant	Violin section	Piece (2D condition)	Piece (3D condition)
1	1	Holst	Dvorak
2	1	Holst	Dvorak
3	2	Dvorak	Brahms
4	1	Dvorak	Holst
5	2	Dvorak	Brahms
6	1	Dvorak	Holst
7	2	Brahms	Dvorak
8	2	Brahms	Dvorak
9	1	Dvorak	Holst
10	1	Holst	Dvorak
11	2	Brahms	Dvorak

**Fig. 2:**
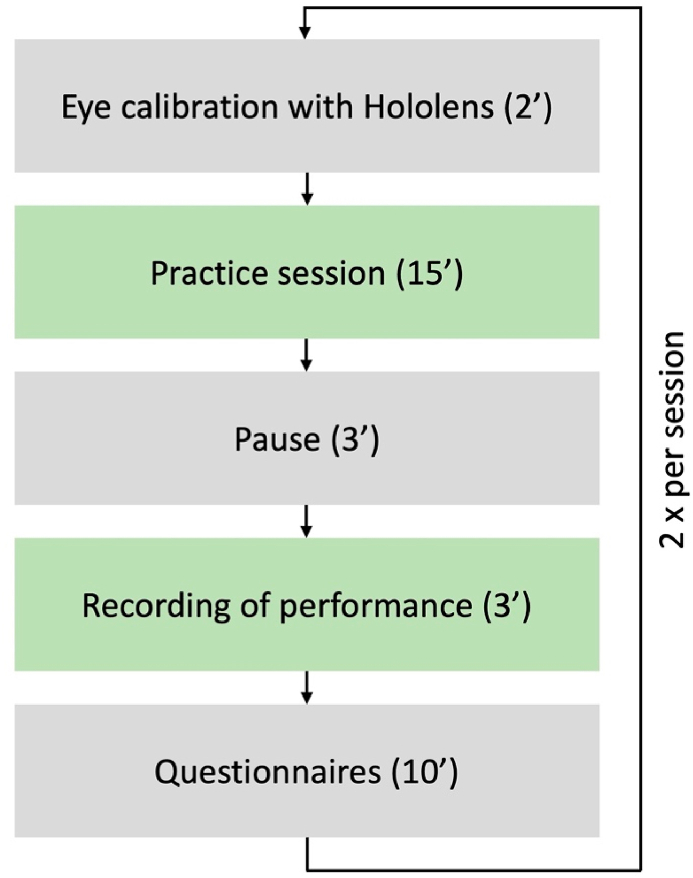
Pipeline of the experimental session. The sequence was repeated for each fragment in the 2 conditions.

For each condition, the participants performed the following steps (Fig. 2): First, the participants practiced the respective fragment with the avatar for 15 min. Then there was a short break, and they were recorded performing the avatar clip. They were asked to mimic bowings, dynamics, and articulation as truthfully as possible. Motion capture data (MoCap), audio, and video of participants were recorded during each full trial and these data were synchronized with the avatar simulations for analysis.

While practicing with the avatar, participants were allowed to walk around and choose their preferred playing position, both in the 2D and 3D condition. However, during the performance measurement, participants had to be in the same position in the MoCap system, and the avatar was always displayed in the same position relative to the participants’ position, so that the avatar was always fully visible in the field of view. Participants were given 15 min at the beginning of each session to practice the music, familiarize themselves with the device, and to flag any problems. This training time allowed the participants to study the pieces – which they did not know before the first trial. Participants could stop, forward and rewind the animation of the avatar at will, to practice and repeat specific passages they found more difficult or skip passages they found easy. During the recorded performance, participants were allowed to play with the score, due to the complexity of the presented music, but also for ecological validity, as scores are usually allowed in orchestra or ensemble context where players sit in a chamber music configuration, so in a circle or semi-circle, which is compatible with the limited FOV of the HoloLens 2. One of the goals of such a rehearsal is to copy the bowings and articulations of the section leader, playing together with the leader while keeping an eye on the score. In a real-life setting, information as presented by the leader is then added to the players' scores so they can reproduce this information during future practice, rehearsals, and performance.

At the end of each trial, participants were asked to complete the Multimodal Presence Scale and Presence Questionnaires along with open-ended questions (see Section 2.6). Then they repeated all the steps with the other condition and the other fragment. The participants were not allowed to practice the fragments between trials (see Fig. 2). At the end of the experiment, 11 × 2 x 4 datasets were obtained (MoCap, audio, video, and questionnaires).

### 2.3 Participants

Eleven participants (3/8 male/female, age 18–25 years) were recruited from the Ghent University Symphonic Orchestra (GUSO, https://guso.ugent.be/, Ghent, Belgium). Six violinists of the first violin section and five violinists of the second violin section participated in the study. All participants played the violin for at least 12 years (mean ± SD = 14.7 ± 2.4 years) and had generally had several years of experience playing in the orchestra (3.7 ± 2.3 years). Furthermore, we used The Goldsmith Musical Sophistication Index (Gold-MSI) to assess their level of musical skill and engagement (4.9 ± 0.5 MSI score) (Müllensiefen et al., 2014). To assess participants’ tendency to become immersed in an artificial environment, we asked them to fill in a standardized self-reported Immersive Tendencies Questionnaire during registration (4.2 ± 0.8 ITQ score) (Witmer & Singer, 1998) (see Table 2).

**Table 2:** Participant demographics. SD is standard deviation, MSI is the music sophistication index, ITQ is the immersive tendencies questionnaire.

	age	age started playing	years played in orchestra	years played violin	MSI	ITQ
mean	21.3	6.6	3.7	14.7	5.1	4.2
median	21.0	6.0	3.0	14.0	5.1	4.1
SD	2.2	1.9	2.3	2.4	0.4	0.8
min	18.0	4.0	0.5	12.0	4.3	3.0
max	25.0	10.0	7.5	20.0	5.8	5.7

### 2.4 Avatars

**Fig. 3:**
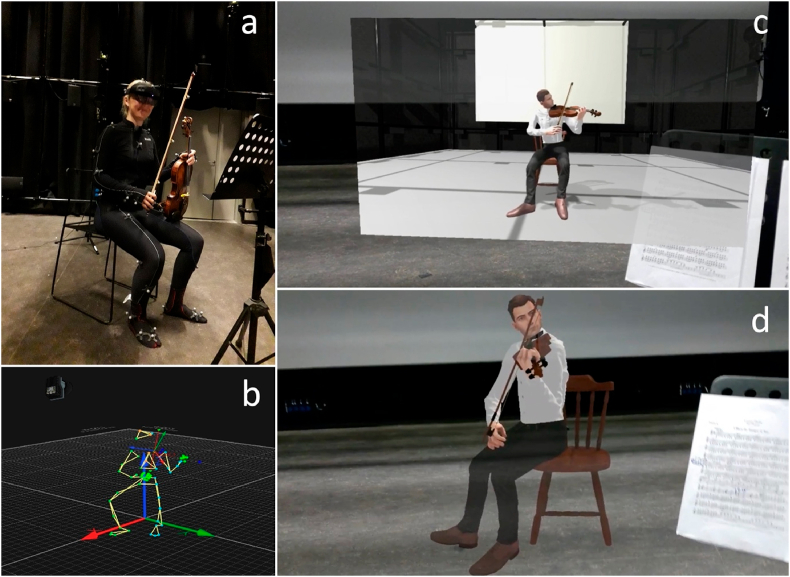
(A) Leader of the second violin in MoCap suit, (b) MoCap recording illustrating the whole-body skeleton, (c) 2D condition, as a 2D projection of a violinist avatar sitting on a chair, and (d) 3D condition, a 3D rendering of the same avatar.

The leader of the first and second violins of the GUSO were recorded playing 2 pieces each. The leader of first violins played: Dvorak: Symphony nr. 8 in G major Op. 88, Part III (bars 1–180) and Holst: The Planets op. 32: I. Mars (bars 17–83; 95–133; 149–167); and the leader of second violins played: Dvorak: Symphony nr. 8 in G major Op. 88, Part I (bars 33–240) and Brahms: Symphony nr. 2 in D major Op. 73, Part III (bars 144–318). These pieces were chosen as to contain the greatest variety in techniques and difficulties, with a minimum number of rests. While recording, the violinists, depending on their preference, played along with either a metronome or an orchestral recording played through headphones. Motion capture data were recorded using a Qualisys MoCap system with 18 cameras including 4 RGB cameras (see Fig. 3a). MoCap and video data were recorded at 120 Hz, and audio was recorded using a Y-pair of condenser microphones at 48,000 Hz and a bit depth of 24 bits. The audio, video and MoCap data were all recorded simultaneously, and were synchronized post-hoc using the SMTPE protocol. The motion capture data were used to create a whole-body skeleton (see Fig. 3b), and based on this skeleton, a male (for the first violin) and a female (for the second violin) avatar was modeled and rigged by the company ARVRtech (ARVRtech, Novi Sad, Serbia).

### 2.5 HoloLens application

**Fig. 4:**
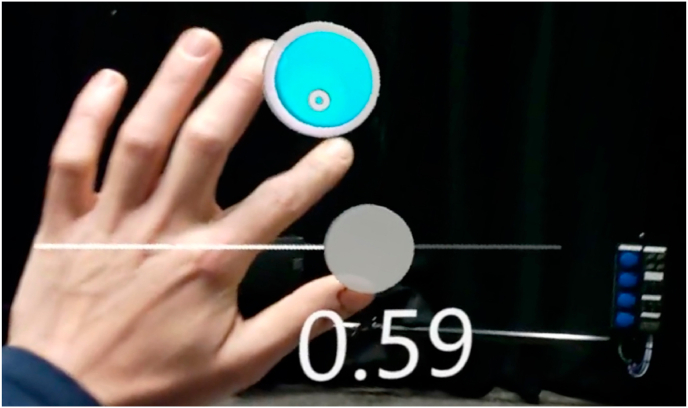
Image of the user interface to stop, start, forward or rewind the avatar.

Avatars of the first and second violin were implemented in a HoloLens application, developed in Unity (Unity version 2020.3.2f1, San Francisco, CA, USA). A 2D condition was created by projecting the avatar in frontal view on a virtual 2D screen (see Fig. 3c). The 3D condition was created with a fully rendered avatar in front of the participants (see Fig. 3d). The performance of the avatar could be started, stopped, forwarded, or rewound by a user interface with a start/stop button and a slider (see Fig. 4). Avatars had different genders, because the section leader of the 1st and 2nd violins was male, respectively female. Matching genders, contributed to the ecological validity of the sectional rehearsal simulation.

### 2.6 Questionnaires

We used the standardized self-reported Presence Questionnaire (Witmer et al., 2005) (hereafter referred to as the Witmer Presence Questionnaire or WPQ) and two sub-sets of questions from the Multimodal Presence Scale for Virtual Reality (Makransky et al., 2017) (hereafter referred to as the Makransky Presence Questionnaire, with social subset MPQS and physical subset MPQP) to inquire about participants’ physical and social presence in a Mixed Reality environment. Participants were asked to fill in these questionnaires after every condition in every trial. In total, each participant filled in the Presence Questionnaire and the Multimodal Presence Scale eight times. In addition, after each session, we asked several open-ended questions regarding the effectiveness of the training in Mixed Reality setup, regarding the similarity of the experience when practicing with a colleague or with the video at home, and regarding possible application improvements. In addition, we probed for the perceived difficulty of the pieces with a self-reported scale from 0 to 100 (easy-difficult).

### 2.7 Data analysis

**Fig. 5:**
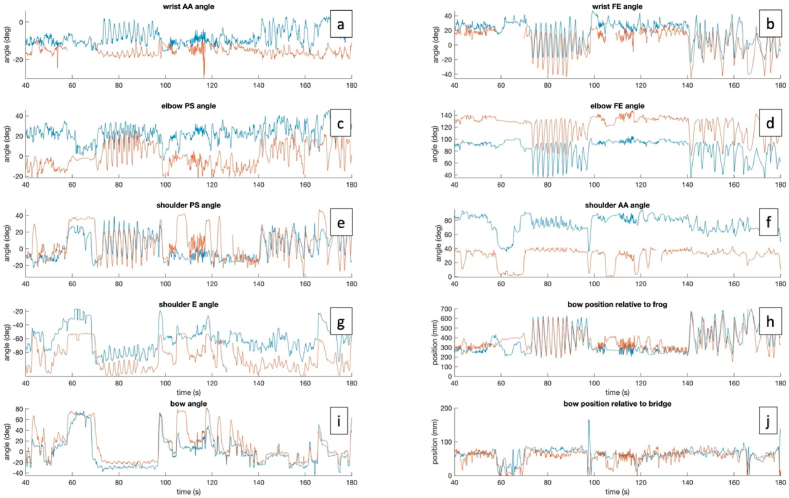
Example of data of both avatar (blue) and a participant (red) of the first violin section, playing Dvorak. Data represent wrist (a,b), elbow (c,d) and shoulder angles (e–g), as well as the distance between contact point between bow and string, and the frog (h) and the bridge (j), respectively, and the tilting angle between the bow and the violin (i). AA = adduction-abduction, FE = flexion-extension, PS = pronation-supination, E = elevation.

**Fig. 6:**
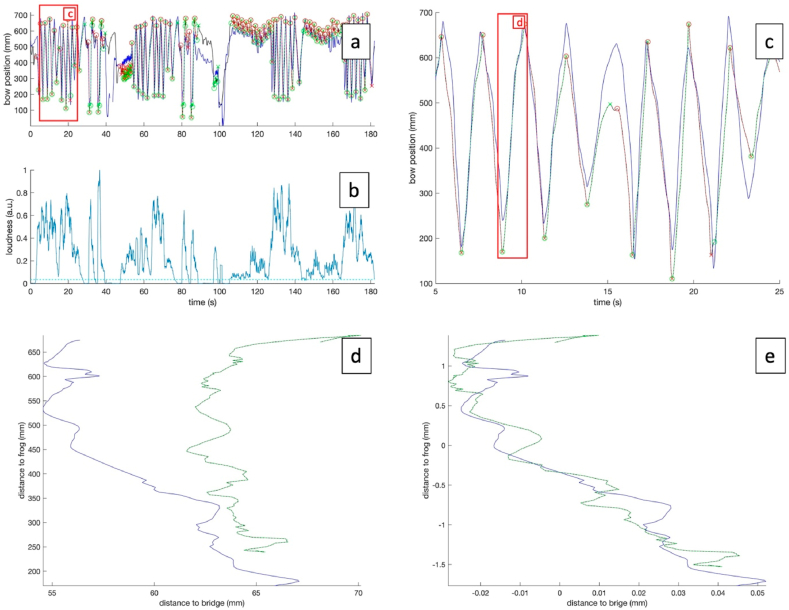
Pipeline illustrating time series comparison between participant and avatar. (a) Represent the bow position of avatar (black) and participant (blue). Green and red dotted lines represent analyzed downstrokes and upstrokes, respectively. (b) Represents the loudness of the performance, the green dotted line represents the loudness threshold for further analysis. (c) Displays a close-up of the red area in (a). (d) Represents a 2D plot of the bow position relative to the bridge (horizontal axis) and the bow position relative to the frog (vertical axis). (e) Alignment of both signals in (d) before Procrustes analysis of the 2D signal. (d) Displays a close-up of the red area in (c).

MoCap data, audio and video data were acquired for every participant. Joint angles of wrist, elbow and shoulder were approximated from the MoCap data using a custom-made MATLAB package (Campo, 2023), based on the standards of the International Society of Biomechanics (ISB) (Grood & Suntay, 1983; Stokdijk et al., 2000; Wu et al., 2002, 2005). Additionally, tilting angle and bow position between the bow and the violin was calculated, respectively defined as the distance of the contact point between bow and string to the bridge and frog (see Fig. 5). Then, regions of interest were extracted based on avatar bow position to isolate individual bow strokes (see Fig. 6). Only regions containing bow strokes exceeding a certain bowing length (150 mm) were kept for further analysis. The audio data of the remaining regions were analyzed on loudness and pitch. Subsequently, of the remaining data, only the regions reaching a certain loudness level (at least 15% of the median loudness) were kept (see Fig. 6). Finally, this set of bow strokes was kept for further analysis as avatar and participant data. To summarize, per bow stroke, following data were obtained:

1. Bow position/velocity/acceleration/jerk
2. Bow angles
3. Wrist/Elbow/Shoulder angles
4. Loudness
5. Intonation
6. Start/end position of the bow

**Fig. 7:**
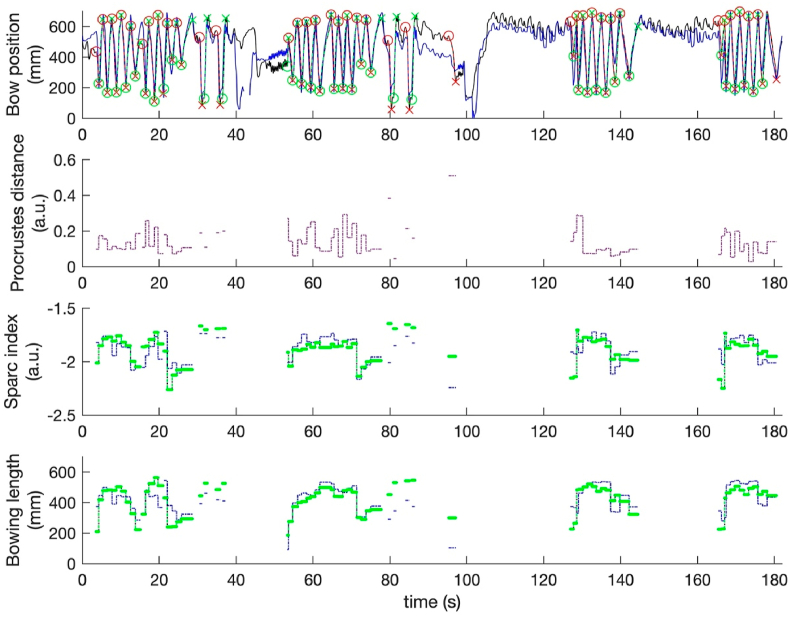
(A) represents the bow position of avatar (black) and participant (blue). Green and red dotted lines represent analyzed downstrokes and upstrokes, respectively. (b) Displays the computed Procrustes distance (PD), of the participant bow strokes as compared to the avatar, and (c,d) displays the SPARC index and bowing length of every participant (dotted blue line) and avatar (dotted green line) bow stroke.

For this study, each bow of between participant and avatar was compared as the 2D curve of bow position using the Procrustes distance (PD) as a metric for difference in shape (Goodallt, 1991; Krishnan et al., 2019) (see Fig. 6). Additionally, the difference in smoothness was computed using the SPARC index (dSI) (Balasubramanian et al., 2015) (see Fig. 7). Participant progress was quantified for each trial using the avatar data as a reference (see Fig. 9).

### 2.8 Statistical analysis

Data from a metric (PD, dSI) were first scaled by subtracting the mean, and dividing by the standard deviation (SD). Then, for each participant's condition in a trial, the participant's individual median over performance time is calculated, giving 11 participants ^∗^ 2 conditions ^∗^ 4 trials = 88 data entries. To account for a possible difference in performance difficulty due to the two pieces and two violin roles, the group median of four groups (piece x violin) is calculated and the participant's group median is subtracted from the participant's individual median, giving a calibrated dataset.

**Fig. 8:**
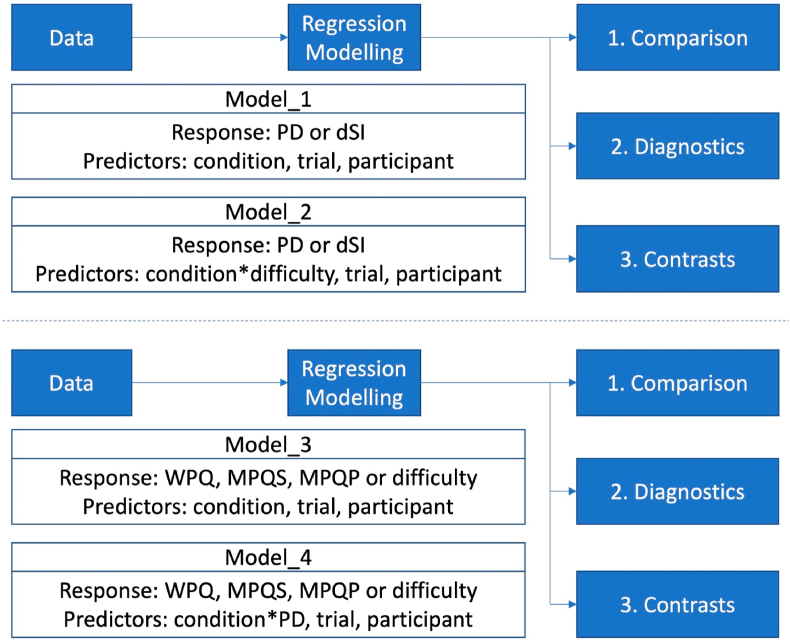
The workflow is divided in two parts. The upper part is the biomechanical workflow (for metrics), the lower part is the presence workflow (for questionnaires). Starting from data we define regression models, which are then compared, diagnosed, and tested for contrasts.

The statistical workflow is shown in Fig. 8. The workflow in the upper pane illustrates the workflow related to the metrics (Hypotheses 1 and 2). Model_1 and model_2 are similar models having either PD or dSI as response, and condition, trial, and participant as predictors. The brms-syntax of the models is:

• model_1: response ∼0 + condition + (1 | condition:participant + condition:trial);
• model_2: response ∼ 0 + condition^∗^difficulty + (1 + difficulty | condition:participant + condition:trial).

**Fig. 9:**
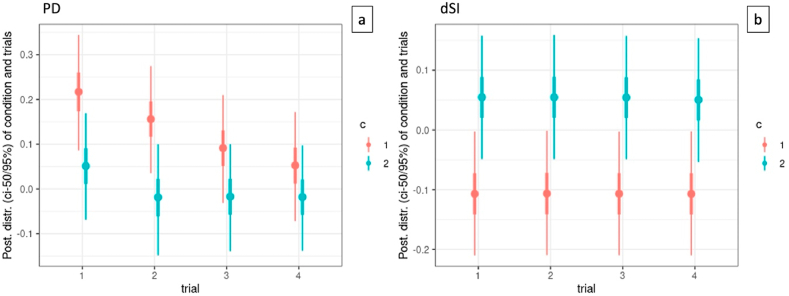
Means of PD (a), dSI (b), per trial per condition. A decreasing/increasing trend can be observed in PD over the different trials. The PD is significantly lower in the 3D condition in each trial, and the dSI is significantly higher in the 3D condition in trial 1–3. PD improved significantly in trial 2, 3 and 4 compared to trial 1, in the 2D condition.

In model_2, a predictor called “perceived difficulty” (difficulty) is added in interaction with condition. We start with (1. Comparison) a comparison of model_1 and model_2 to test whether difficulty should be added to the model. We then perform (2. Diagnostics) a more detailed diagnostics of the best model, as well as (3. Contrasts) a contrast analysis of condition and trials.

The lower pane of Fig. 8 illustrates the workflow related to the questionnaires (Hypotheses 3 and 4). Model_3 and model_4 are similar models having one of the questionnaires as response (WPQ, MPQS, MPQP, and perceived difficulty) except that in model_4, PD is added in interaction with condition. The brms-syntax of the models is:

• model_3: response ∼0 + condition + (1 | condition:participant + condition:trial);
• model_4: response ∼ 0 + condition^∗^PD + (1 + PD | condition:participant + condition:trial).

We start with (1. Comparison) a comparison of model_3 and model_4 to test whether PD should be added to the model. We then proceed with (2. Diagnostics) a more detailed diagnostics of the best model, and (3. Contrast) contrast analysis.

All models are fitted with the R-package brms (Bürkner, 2018). The model comparison is based on the log ratio of the marginal likelihood of two models using the Bayes-factor test (using bayes_factor), and a supplementary check with a leave-one-out cross-validation (loo). The diagnostics of the models was done with pp_check for a global retrodiction check, and mcmc_plot for an overview of the posterior distributions of parameters. We also run a bayes_R2 (Gelman et al., 2019) to get an estimate of the variances, and we use parameters for summary of the model. In testing the contrast of model parameters, we code trials as factors because, in the context of the present study, order was considered more relevant than time between the trials. We use one-sided hypothesis testing, using the posterior probability under the hypothesis against its alternative. The reported posterior probability of direction (pd) represents the certainty with which an effect goes in a particular direction (i.e., is positive or negative). Strong evidence has a pd above 0.95. See Supplementary Material for details.

Note that the hierarchical modelling approach prevents overfitting by shrinking the instances of the group-levels towards the means of the respective group-levels (McElreath, 2020). Another way of looking at these regressions is that they capture the variability related to participant and trial, leaving the variability of interest to condition, which is the focus of our analysis. In Hypothesis 1, it is expected that the PD mean of the 3D condition is lower than the PD mean of the 2D condition, while it is the reverse in the dSI metric. In Hypothesis 2, the effect of Trial on Condition is investigated to see if trials point to improved musical performance quality. If learning is involved, then the means should decrease in the PD metric, and increase in the dSI metric. In Hypothesis 3 and 4, we test whether the different conditions induce different levels of presence and whether levels of presence depend on the metric.

We run the models on a 48 dual core machine (at Ghent University, IPEM), using the R package brms. We take 5000 warmups and 40,000 iterations, with an adapt_delta = 0.995 and max_treedepth = 12, 4 chains, and 24 threads. The large number of iterations was needed in view of a stable Bayes factor test in the R package parameters.

## 3 Results

### 3.1 Motion capture

From the motion capture data, joint angles/angular velocities/angular accelerations were obtained from right shoulder, elbow, and wrist joints. Partial results are displayed in Table 3. The bow position/velocity and acceleration were also obtained, resolved relative to the frog of the bow and the bridge of the violin, as well as angles of the bow relative to the instrument (see Table 3).

**Table 3:** Motion capture parameters. Following joint angles (a) and angular velocities (aV) were approximated for the wrist, elbow, and shoulder joints: Flexion-extension (FE), pronation-supination (PS), elevation (E), adduction-abduction (AA). For the bow, position relative to frog/bridge (s), velocity (v) and acceleration (Acc) were calculated. Mean is reported as mean ± SD. Minimum (min) and maximum (max) values are also given. Values are reported here for first (violin 1) and second violin (violin 2).

			Violin 1			Violin 2		
	parameter		Mean ± SD	min	max	Mean ± SD	min	max
elbow angles	FE	a	94.7 ± 8.6	83.8	102.8	97.9 ± 4.2	93.3	102.0
		aV	26 ± 25	1	81	47 ± 29	5	86
	PS	a	33.0 ± 4.9	27.1	37.8	44 ± 16	40	48
		aV	24 ± 55	1	131	0.5E02 ± 5.7E02	0.0E02	3.1E02
shoulder angles	E	a	−58.0 ± 4.4	−62.6	−53.7	−71.5 ± 1.5	−72.8	−70.2
		aV	14 ± 22	1	56	13 ± 12	3	28
	AA	a	46.6 ± 7.8	40.0	56.1	51 ± 14	48	54
		aV	23 ± 22	1	68	0.3E02 ± 5.2E02	0.0E02	2.0E02
	PS	a	19.1 ± 9.2	7.9	27.4	20 ± 18	13	25
		aV	28 ± 28	1	88	0.7E02 ± 8.3E02	0.0E2	3.7E02
wrist angles	FE	a	5.9 ± 7.7	−2.4	14.3	−5.0 ± 2.9	−8.0	−2.3
		aV	26 ± 24	1	94	30 ± 25	3	74
	AA	a	1.3 ± 1.8	−1.1	3.7	3.0 ± 1.6	1.2	4.8
		aV	15 ± 22	0	86	23 ± 21	2	62
bow angle	AA	a	88.2 ± 2.0	85.8	90.5	85.79 ± 0.99	84.69	86.85
bow	dist frog	s	477 ± 73	388	560	430 ± 34	395	465
		v	2.3E02 ± 1.4E02	0.2E02	4.6E02	3.4E02 ± 1.9E02	0.4E02	0.6E02
		Acc	1.9E03 ± 2.8E03	0.1E03	8.5E03	6.5E03 ± 4.5E03	0.6E03	13.7E03
	dist bridge	s	66.4 ± 5.3	60.2	72.5	54.4 ± 3.8	49.9	59.2
		v	30 ± 38	1	105	70 ± 43	8	138

### 3.2 Questionnaires and qualitative data

Cronbach's alpha was calculated for MPQS (0.82), MPQP (0.85), WPQ (0.90) and MSI (0.80). Analysis of questionnaire data suggests all participants favored rehearsals with 3D avatars independently from the group, music excerpt, and the trial number.

**Fig. 10:**
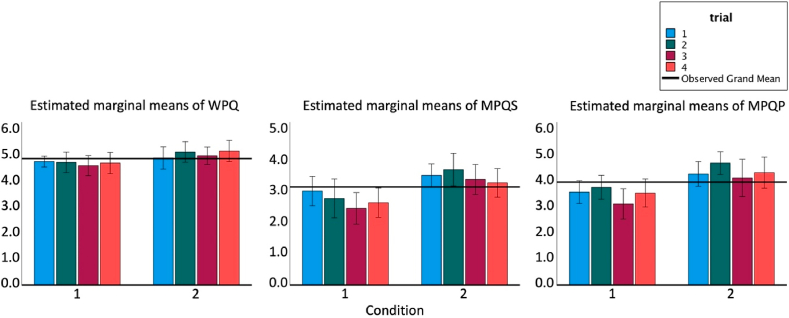
Marginal means of the Witmer Presence questionnaire (WPQ), and the social and physical Makransky multimodal presence scale (MPQS and MPQP), per trial per condition.

Furthermore, there were significant differences between the 2D and 3D conditions regarding participants’ judgments of social and physical presence (see Table 4, Fig. 10, section 3.3) as well as in the open-ended questions. In relation to the 3D condition, 44.2% of the participants responded positively to a seven-point Likert scale inquiring “how similar was the experience to practicing with your colleague?” (Median = 4, IQR = 2). 20.4% of participants were neutral and 34.9% did not find the experience to be familiar with practicing with a colleague.

**Table 4:** Mean ± SD of the Witmer Presence Questionnaire (WPQ), the Makransky multimodal presence scale (MPQ), and the subsets of MPQ: social MPQ (SMPQ) and physical MPQ (PMPQ) for each trial (1–4) and each condition (2D/3D).

trial	condition															
	2D								3D							
	WPQ		MPQ		SMPQ		PMPQ		WPQ		MPQ		SMPQ		PMPQ	
1	4.79	±0.30	3.37	±0.82	3.09	±0.90	3.65	±0.85	4.93	±0.60	3.96	±0.69	3.60	±0.86	4.33	±0.72
2	4.65	±0.55	3.20	±0.59	2.66	±0.77	3.74	±0.66	5.17	±0.56	4.26	±0.70	3.76	±0.84	4.76	±0.74
3	4.63	±0.54	2.85	±0.82	2.53	±0.91	3.18	±0.91	5.01	±0.49	3.87	±0.90	3.48	±0.89	4.3	±1.1
4	4.73	±0.57	3.14	±0.67	2.69	±0.75	3.58	±0.77	5.21	±0.61	3.9	±1.1	3.4	±1.1	4.4	±1.1

In relation to the 2D condition, on the question “how similar the experience was, to practicing with a video?” (Median = 4, IQR = 2), 34.9% of participants responded positively, 23.3% were neutral and 41.9% did not rate the experience as being similar to practicing with video at home. On the question inquiring about the effectiveness of the training in 3D condition (Median = 5, IQR = 1), 95.4% of participants rated the effectiveness of training positively and 4.7% were neutral. In the 2D condition (Median = 5, IQR = 1), the effectiveness of training was rated positively by 72.1% participants, by 11.6% of them as neutral, and by 16.3% as ineffective. Participants’ suggestions for application improvement included the possibility to incorporate audio of the orchestra, the possibility to play in different tempi (especially difficult passages), and the generation of audio feedback (in form of verbal comments) or visual feedback on the overall musical performance quality.

The perceived difficulty of the 4 different pieces is given in Table 5.

**Table 5:** Mean piece difficulty ± SD, per participant per piece as averaged over 4 trials. Difficulty was self-assessed, on a scale from 0 to 100.

	Perceived difficulty (scale 0–100)			
	piece			
Participant	1	2	3	4
P001	24 ± 12	47 ± 11		
P002	10.0 ± 5.0	21.3 ± 8.8		
P003			50 ± 14	50.3 ± 8.3
P004	55.3 ± 8.0	34 ± 16		
P005			85.5 ± 5.4	66.8 ± 6.8
P006	66.5 ± 9.8	27.3 ± 8.1		
P007			28.7 ± 9.1	57 ± 13
P008			45.3 ± 5.7	33.0 ± 4.6
P009	67.3 ± 7.0	41 ± 16		
P010	27.0 ± 8.4	39 ± 10		
P011			49 ± 20	41 ± 14

### 3.3 Statistical analysis

#### 3.3.1 Comparison

The Bayes factor is in favor of model_1 over model_2, having values of 4.1 for PD and 5.7 for dSI, meaning that the perceived difficulty has no added value for a further analysis of the biomechanical metrics. The Bayes factor is in favor of model_3 over model 4, having values of 4.2 for WPQ, 2.8 for MPQS, 3.4 for MPQP, and 2.2 for the perceived difficulty question, meaning that despite some anecdotical evidence for Difficulty, PD has no added value for a further analysis of the presence.

#### 3.3.2 Diagnostics

All models (of type model_1, model_3) converged normally. Posterior prediction checks (pp_check), based on visual inspection, revealed that the models have an acceptable close fit. Details of the analysis can be consulted in the Supplementary Materials. The conditional R2 (variance of fixed and random effects) and the marginal R2 (variance of the fixed effects) is 0.53 and 0.06 for PD and 0.65 and 0.15 for dSI. For WPQ it is 0.73, 0.12, for MPQS it is 0.66, 0.18, for MPQP it is 0.65, 0.22, and finally for Difficulty it is 0.62, 0.06.

#### 3.3.3 Contrasts

A summary of the contrasts is given in Table 6 for PD, and in Table 7 for dSI. The labels in the first column mark the contrasts of posterior distributions given by conditions and trials. For example, c12 is the posterior distribution for condition 1 minus condition 2, c1t12 is the posterior distribution of condition 1 for trial 1 minus trial 2, c12t1 is the posterior distribution of trial 1 for condition 1 minus condition 2, and so on. The second column gives the difference in units of the response (PD, dSI), with columns CI.Lower and CI.Upper marking the critical interval of 95% of this estimate. The column Post.Prob gives the pd or probability of direction of the posterior marked by the label.

**Table 6:** Contrasts for the PD metric. The labels on the left mark the contrast of posterior distributions given by condition and trial. As shown in Table 7 for the dSI metric, the posterior difference of c12 has a pd of 0.98, in favor of condition 1 > condition 2, indicating that the estimated mean tends to be different, which is in strong support of Hypothesis 1. Contrasts among trials, both for condition 1 and condition 2 show pd values around 0.50 meaning that there is no effect, and thus no difference over trials. Given the strong difference between condition 1 and condition 2, and no effect over trials, we see pd values of 0.02 (to be interpreted as 0.98) for contrasts of trials over conditions, meaning that over trials, there remains a considerable distinction between conditions.

Label	Estimate	CI.Lower	CI.Upper	Post.Prob
c12	0.13	−0.03	0.28	0.09
c1t12	0.06	−0.02	0.15	0.88
c1t13	0.13	0.03	0.22	0.99
c1t14	0.16	0.06	0.26	1.00
c1t23	0.07	−0.01	0.14	0.91
c1t24	0.10	0.02	0.19	0.98
c1t34	0.04	−0.04	0.12	0.80
c2t12	0.07	−0.01	0.16	0.92
c2t13	0.07	−0.01	0.15	0.93
c2t14	0.07	−0.01	0.15	0.93
c2t23	0.00	−0.08	0.08	0.48
c2t24	0.00	−0.08	0.08	0.49
c2t34	0.00	−0.08	0.08	0.51
c12t1	0.17	0.03	0.31	0.97
c12t2	0.18	0.04	0.32	0.98
c12t3	0.11	−0.03	0.25	0.90
c12t4	0.07	−0.07	0.21	0.81

**Table 7:** Contrasts for the dSI metric. The labels on the left mark the contrast of posterior distributions given by condition and trial. As shown in Table 8 for the questionnaire metrics, the posterior difference of c12 has a pd of >0.95 for WPQ, MPQS and MPQP, in favor of condition 1 < condition 2, indicating that the estimated mean tends to be different, which is in support of Hypothesis 3, meaning that different conditions induce different levels of presence, and perceived piece difficulty in the participants.

Label	Estimate	CI.Lower	CI.Upper	Post.Prob
c12	−0.16	−0.28	−0.04	0.98
c1t12	0.00	−0.03	0.03	0.49
c1t13	0.00	−0.03	0.03	0.49
c1t14	0.00	−0.03	0.03	0.50
c1t23	0.00	−0.03	0.03	0.50
c1t24	0.00	−0.03	0.03	0.51
c1t34	0.00	−0.03	0.03	0.50
c2t12	0.00	−0.03	0.03	0.50
c2t13	0.00	−0.03	0.03	0.51
c2t14	0.00	−0.02	0.04	0.58
c2t23	0.00	−0.03	0.03	0.51
c2t24	0.00	−0.02	0.04	0.58
c2t34	0.00	−0.02	0.04	0.57
c12t1	−0.16	−0.28	−0.04	0.02
c12t2	−0.16	−0.28	−0.04	0.02
c12t3	−0.16	−0.28	−0.04	0.02
c12t4	−0.16	−0.28	−0.04	0.02

**Table 8:** Contrasts for WPQ, MPQS, MPQP, for condition 1 < condition 2.

Label	Estimate	CI.Lower	CI.Upper	Post.Prob
c12(WPQ)	−0.38	−0.75	0	0.95
c12(MPQS)	−0.81	−1.42	−0.21	0.98
c12(MPQP)	−0.9	−1.5	−0.31	0.99

As shown in Table 6 for the PD metric, the posterior difference of c12 has a pd of 0.09, but this should be interpreted as 0.91 evidence in favor of condition 2 > condition 1, indicating that the estimated mean tends to be different, which is in support of Hypothesis 1. When conditioned by condition 1, the posterior probabilities reveal that Trial1 » Trial3, and Trial1 » Trial4, and Trial2 > Trial3 and Trial 2 » Trial4 (> is a weak effect with pd 0.88 and 0.99, » is a strong effect, with pd above 0.95). This finding suggests a trend of decreasing mean in PD metric, pointing to a learning effect. When conditioned by condition 2, the posterior probabilities reveal that Trial1 > Trial2, Trial 1 > Trial3, Trial1 > Trial4, meaning that apart from a learning effect after Trial1, there is no learning effect over trials. Considering contrasts of trials over conditions, the effect decreases. This means that over trials, there is a trend towards less distinction between conditions.

To sum up, Hypothesis 1 is confirmed in the PD and dSI metric, where the mean of the 3D Condition is lower (for PD) and higher (for dSI) than the mean of the 2D condition. Hypothesis 2 is only partly confirmed in the PD metric with trials in the 2D condition pointing to a learning effect, while trials in the 3D condition show an effect after the first trial but not for the other trials. In contrast, the dSI metric shows no learning effect (see Fig. 9). Hypothesis 3 is confirmed as the 2D condition, having lower values, differs from the 3D condition (see Fig. 10). Hypothesis 4 is not confirmed meaning that the levels of presence do not depend on the quality of the performed task.

## 4 Discussion

In the present study, a small group of violin players practiced their bowing gestures along with a simulation of their concertmaster - in the form of an avatar - four times over the course of a month. The AR simulations were either a 2D stereoscopic projection of an avatar, or a 3D stereoscopic projection of the same avatar on the HoloLens 2. The avatar concertmaster played a piece with specific bowings, articulations, and dynamics, and as in a real rehearsal, participants had to mimic their leader's playing as closely as possible, to function as a cohesive whole during the orchestral performance. With the HoloLens app, participants were free to start, stop, forward or rewind the 2D/3D simulation whenever they wanted, using an interactive 3D interface during the practice sessions, but they needed to play along from beginning to end without interruptions during the recording sessions. During the experiment, participants learned their gestures through imitation, which was evaluated using a measure based on their ability to synchronize with the avatar and how this synchronization improved across trials. This paradigm was chosen because it circumvents the problems of quantifying the quality of musical performances without having a reference at one's disposal, and because imitation learning is one of the most common ways of studying music (Spilka et al., 2010). After each performance, participant's feeling of (social) presence was assessed as well.

In line with Hypothesis 1, the 3D condition has a favorable effect on the musical performance quality, as compared to the 2D condition, in both the PD and the dSI metric, across all trials. This result confirms earlier finding that stereoscopic information, as presented in the 3D condition, can positively influence the success of imitating 3D gestures (Anderson et al., 2019). In addition, it has been shown that the spatial cues of a 3D rendering provide benefits for understanding and memorization of study material (Ragan et al., 2010).

Hypothesis 2 was only partly confirmed. The data show a learning effect in the PD metric for the 2D condition, but not for the 3D condition, while there is no learning effect for the dSI metric. A possible explanation is that the PD metric in the 3D condition was already better than in the 2D condition, leaving less room for improvement and thereby not meeting our detection limits. The PD and dSI metric remained better in the 3D condition independently of the trial (cf. Hypothesis 1).

In line with Hypothesis 3, participants reported a higher sense of presence and social presence in the 3D condition, as assessed by the WPQ, MPQS and MPQP. This finding corresponds with the literature suggesting that both a higher level of realism and a humanoid avatar can enhance the sense of presence (Bowman et al., 2009; Ijsselsteijn et al., 2001; Kober et al., 2012) and social presence (Yoon et al., 2019). In addition, research focusing on adult involvement in music suggests that adults particularly value social interaction (Roulston et al., 2015; Zhukov, 2021), and for amateur musicians, participation in weekly orchestral rehearsals is crucial for their musical development and motivation. Results showing a heightened sense of (social) presence generated by practicing with a 3D avatar may have important implications for traditional instrumental training, for example, when orchestral rehearsals are physically not possible, as was the case during the recent covid pandemic and the resulting closure of all musical and group activities.

As far as Hypothesis 4 is concerned, the 2D and the 3D condition appear to induce different levels of presence, but the musical performance quality had no significant added effect. An explanation may be that presence was assessed after each trial, obscuring causality claims.

Overall, participants indicated that they liked the 3D condition better, which may have affected their engagement and motivation (Sung et al., 2018). Also, there is a potential “WOW effect” (Makransky et al., 2019a) with the 3D simulation, potentially influencing the results. Finally, participants reported different user interactions when using either the 2D or the 3D condition, with some participants reporting reliance on auditory and visual cues in the 3D condition, while relying mainly on auditory cues in the 2D condition.

Also, caution is needed when interpreting presence as assessed by questionnaires (Grassini & Laumann, 2020; Nannipieri, 2022). The first definitions of presence appeared when virtual reality was much more prevalent than mixed reality. Presence in a VR context is thereby described generally as the feeling of “being there”, typically assessed by presence scales as developed by Witmer et al. (Witmer et al., 2005; Witmer & Singer, 1998). Unfortunately, as discussed by Lee et al. (Lee, 2004), this definition of presence (or telepresence) is not readily applicable along the entire Milgram Virtuality Continuum, and it is not clear how the beneficial effects of presence translate from highly immersive environments to the lower ones (Lee, 2020, pp. 1–20; Toet et al., 2022; Weidner et al., 2023). In AR, immersion entails: (1) to generate a high-quality rendering of virtual objects, (2) to precisely register the virtual objects with the real environment, and (3) to do so in an interactive real-time (Regenbrecht & Schubert, 2002). Accordingly, it can be argued that, in AR applications, presence is related to the extent to which the line between real and virtual becomes blurred (McCall et al., 2011), rather than having the feeling of being physically in a different place. Since the original concept of presence was updated with the advent of AR, direct comparisons between VR and AR studies become more complicated (Lee, 2004). In the present work, we will stay close to the definition as posed by Lee et al. (Lee, 2004), distinguishing physical, social and self-presence as assessed with e.g., the multimodal presence scale as designed by Makransky et al. (Makransky et al., 2017). However, if presence can influence the task performance, it is not known how the different subcategories of social, physical, or self-presence affect task performance individually, nor how they interact while doing so. Additionally, the small sample size (N = 11) in this study, makes it unfeasible to make general claims about the relationships between musical performance quality and presence or learning and presence.

There is much discussion in the literature about the interaction between presence and learning (Kaplan et al., 2021). Given the ongoing debate, the issues with defining and assessing presence, and the small sample size in the present study, it is not surprising that we failed to confirm Hypothesis 4. However, if at least some of the reported effects are associated with presence, the current findings are fairly in line with studies showing better task performance with higher presence (Grassini et al., 2020). Moreover, higher presence could help explain the smoother movements in the 3D condition, hypothesizing that the heightened sense of presence serves as an external focus of attention, improving the smoothness and automation of movements (Kal et al., 2013). Also, given the difference in learning between the 2D and 3D condition, it can be hypothesized that in this particular type of motor imitation learning, the effect of presence is smaller than expected (Mattar & Gribble, 2005), as motor imitation tasks are fairly resilient to changes in focus and attention.

To sum up, the results indicate that the 3D condition yields better similarity in gestures, smoother movements, and a higher sense of presence compared to the 2D condition. However, learning effects were only observed in the 2D condition and not in the 3D condition. Overall, the effect size of AR-based learning is more significant in musical performance quality than in learning. The study did not find a significant correlation between presence and musical performance quality.

Furthermore, this study has shed light on the impact of social presence on students' learning outcomes and experience, using 2D/3D avatar representations of teachers or lead violinists. The findings suggest that social presence plays a crucial role in enhancing students' learning outcomes, as demonstrated by improved musical performance quality and a more positive learning experience. These insights could prove valuable for the development of online education and immersive technologies, as they provide evidence of the potential role of virtual teachers in AR.

Finally, incorporating biomechanical metrics provides an objective measure of students' performance (e.g. (Talebi et al., 2023),), or motor learning outcomes (e.g. (Konczak et al., 2009),), overcoming the limitations of traditional self-reports. This approach can be especially useful when combined with the body tracking functionality available in the most recent VR/AR headsets (Movement SDK for Unity, 2023).

## 5 Limitations

Finally, the results presented in this study should be interpreted with caution as many confounders are likely to be present, such as differences between tasks, differences in physical and mental fitness between trials, participant variance, among others. However, it should be noted that measures have been taken to neutralize these confounders as much as possible, both in the design of the experiments and in the preparation of the data and the statistical analysis. The order of conditions was randomized across trials, data were normalized to compensate for differences in tasks, and perceived difficulty was included in the statistical analysis. Variance due to participants and trials was captured in the statistical modeling, and the population was chosen to be highly homogeneous in terms of age and musical background. In addition, physical and mental fitness were assessed, but these data were not included in our statistical analysis. A retrospective power analysis confirmed that a Bayesian statistical analysis was the right approach to deal with the limited number of participants in this study.

## 6 Future prospects

As the data suggest, user experience was highly dependent on participant. In line with the data, different behaviors of participants were observed, especially in relation to slider use such as rewinding, and in seating position. Also, in their answers to open-ended questions, participants reported different needs and uses for such applications, i.e., some found it effective and helpful to practice only with the audio play-back of their violin part, while others would rather practice with the audio play-back of the whole orchestra. Further research is needed to explore the unique ways in which participants engage with these kinds of applications, to improve their functionalities and tailor them to individual needs (Michalko, Campo, Nijs, Leman, Van Dyck).

Additionally, the HoloLens application and rendered avatars used in this study did not include precise fingerings and therefore participants could not improve the fingerings of their left hand. In future experiments, we aim to include fingering information.

Although the HoloLens headset proved to be a promising tool for practicing and performing, its limited field of vision posed certain challenges for participants. However, these limitations and other drawbacks will be addressed in future experiments to enhance the ecological validity of using AR for practicing and performing.

## Credit author statement

**Adriaan Campo**: Software, Methodology, Conceptualization, Validation, Formal analysis, Investigation, Resources, **Data curation**, Writing – original draft, Visualization; **Aleksandra Michałko**: Conceptualization, Investigation, Methodology; **Bavo Van Kerrebroeck**: Software, Methodology, Conceptualization, Writing – review & editing; **Maja Pokric**: Software; **Boris Stajic**: Software; **Marc Leman**: Software, Methodology, Conceptualization, Formal analysis, Investigation, Data curation, Writing – original draft, Visualization, Supervision, Funding acquisition.

## Declaration of competing interest

The authors declare that they have no known competing financial interests or personal relationships that could have appeared to influence the work reported in this paper.

## Data Availability

Data will be made available on request.
