# Supplementary material for: The assessment of presence and performance in an AR environment for motor imitation learning: A case-study on violinists
Source: Comput Human Behav. 2023 Sep;146:107810. doi: 10.1016/j.chb.2023.107810 (PMC10305781; doi:10.1016/j.chb.2023.107810)
Supplement: Multimedia component 1 [file mmc1.pdf]

Supplementary Materials

Table of Contents

Statistical Analysis 2

Data Analysis 34

Detailed workflow: 34

PD MATLAB code: 34

SI MATLAB code: 35

Questionnaires: Musical Background 36

Questionnaires: 2D condition 63

Questionnaires: 3D condition 85

Statistical Analysis

Supplementary Material

2022-10-27

The present document contains details about the data-analysis for the paper entitled: "The assessment of presence and performance in an AR environment for motor imitation learning: a case-study on violinists." Authors: Adriaan Campo, Aleksandra Michałko, Bavo Van Kerrebroeck, Boris Stajic, Maja Pokric, Marc Leman. Basically, the paper tests a violin playback system. The violinist's task is to play in synchrony with the principal violinist of an orchestra (represented as an avatar). The playback system provides the audio, in addition to a 2D or 3D avatar via a Hololens. The focus is mainly on the effect due to the experimental conditions of a 2D or 3D playback.

Workflow

Figure 1 shows two statistical workflows. The upper workflow on top is about the biomechanical metrics from motion capture (here called: procustus and sparc, instead of PD and dSI as in the paper). Each metric represents differences between the violinist and the avatar, summarized over time. These values are used as response in regression models, whereas conditions, participants and trial are used as predictors. Model_1 and model_2 are similar models except that in model_2 `difficulty` is added in interaction with condition. The lower workflow is about the behavioral metrics of the questionnaires. We have 3 presence questionnaires. Model_3 and model_4 are similar models except that in model_4 `procustus` is added in interaction with condition.

In the upper workflow, we start with a comparison of model_1 and model_2 to test whether `difficulty` should be added to the model. We then perform a more detailed diagnostics of the best model, as well as a contrast analysis of condition and trials. In the lower workflow, we start with a comparison of model_3 and model_4 to test whether `procustus` should be added to the model. We then proceed with a more detailed diagnostics of the best model, as well as a contrast analysis of conditions.

The workflows hold a scheme for testing the work hypothesis, as shown in figure 2:

Hypothesis 1. Students will show better violin performance in the 3D condition compared to the 2D condition:

- 1.1. Similarity between virtual teachers' and students' bow movement is higher .
- 1.2. Movement smoothness is higher

Hypothesis 2. Learning effectiveness of violin performance will be higher in the 3D condition compared to the 2D condition.

Hypothesis 3. The 3D condition will induce a higher level of presence compared to the 2D condition:

- 3.1. "physical presence" will be higher
- 3.2. "social presence" will be higher

Hypothesis 4. The level of presence in AR influences students' violin performance.

In hypothesis 1, we first compare model_1 and model_2 and focus on the contrast analysis of the best model. This analysis will tell us the differences between conditions. In hypothesis 2, we expand our contrast analysis by comparing conditions and trials. In hypothesis 3, we do a contrast analysis of model_3.
In hypothesis 4 we test whether the procustus metric might be a relevant predictor variable.

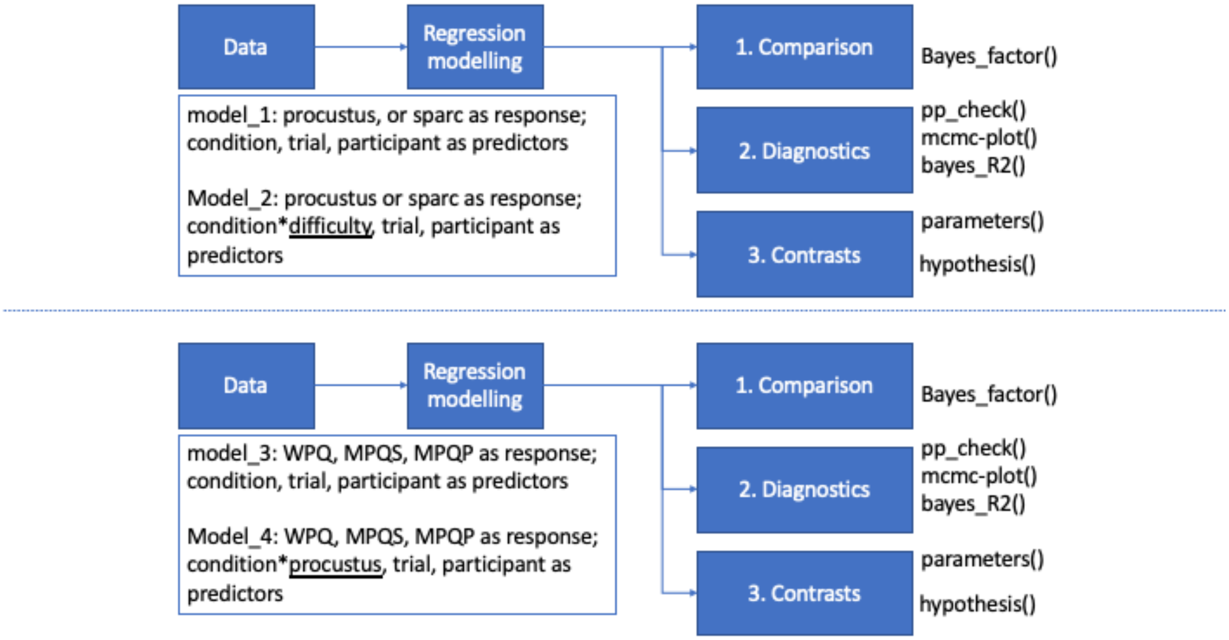

Figure 1: Statistical path

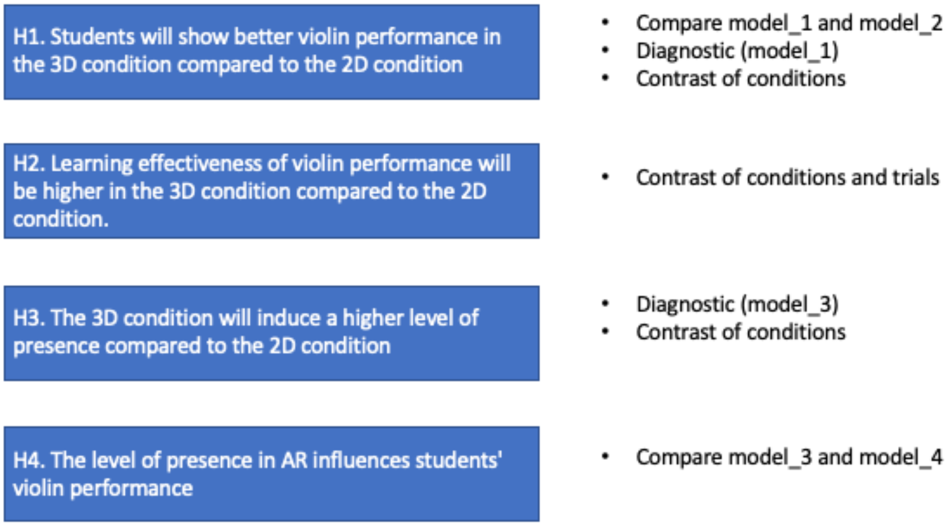

Figure 2: Hypothesis testing

Power Analysis

A retrospective power analysis of the setup shown in Figure 3 reveals that 11 participants and 4 repeated measures has sufficient power when Cohen's D is > 0.6. To show that, we used a hierarchical statistical model with 2 conditions, and with participants and trials modeled as groups for which we assumed a standard deviation of 0.25 and 0.1, respectively. We tested Cohen's D from 0 to 0.6, and a number of participants from 5 to 30. For each dot in the graph below (e.g. D = 0.5, number of participants = 20) we simulated 500 models and tested whether the contrast of conditions has a probability mass of >= .95. The proportion of yes (versus no) is presented as power (in %). The results show that 11 participants have enough power (> 80%) when Cohen's D is > 0.6, meaning that there is <20% to miss an effect when there is in fact an effect (= false negative). In our models, we observe that the calibrated models have D > 0.8 (for the models: see below).

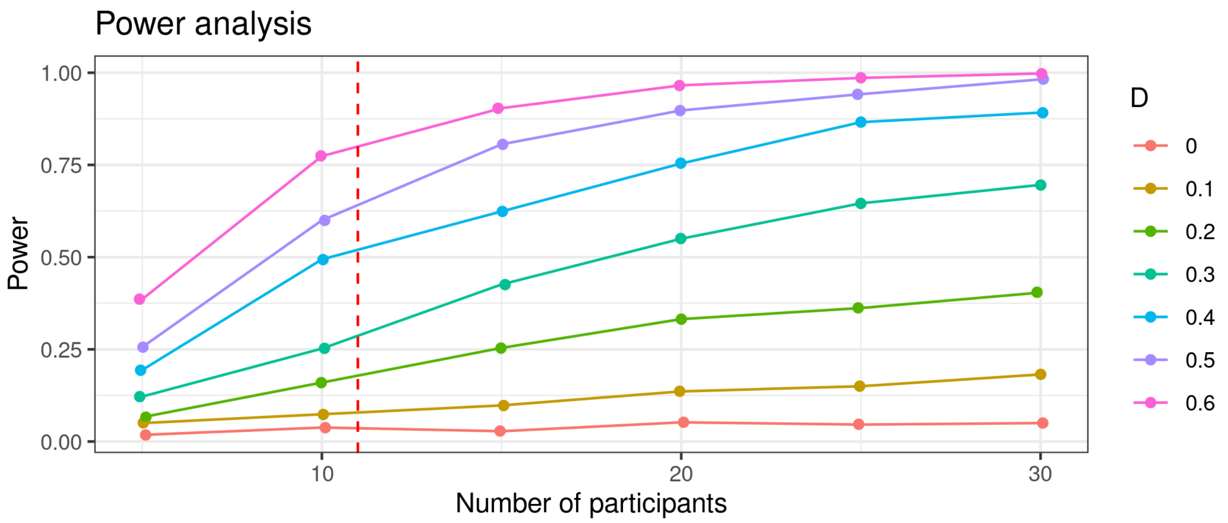

Figure 3: Retrospective power analysis. The dotted vertical line marks 11 participants, as used in thep present study

Data

The first box in the workflow shown in Figure 1 is about data. We use one dataset for all models. The data variables are listed below:

```
load(file = "Data.RData")
str(Data)
```

```
## 'data.frame':    88 obs. of  20 variables:
##  $ participant           : Factor w/ 11 levels "1","2","3","4",..: 1 1 1 1 1 1 1 1 10 10 ...
##  $ condition             : Factor w/ 2 levels "1","2": 1 1 1 1 2 2 2 2 1 1 ...
##  $ trial                 : Factor w/ 4 levels "1","2","3","4": 1 2 3 4 1 2 3 4 1 2 ...
##  $ response1M_procustus  : num  0.5573 0.0622 0.1376 -0.0763 -0.0569 ...
##  $ response0M_procustus  : num  1.332 0.837 0.913 0.699 0.44 ...
##  $ response1M_sparc      : num  -0.0457 -0.0512 -0.2052 -0.0481 0.2803 ...
##  $ response0M_sparc      : num  10.8 10.8 10.6 10.8 10.9 ...
##  $ Age                   : num  23 23 23 23 23 23 23 23 18 18 ...
##  $ MSI                   : num  5.46 5.46 5.46 5.46 5.46 5.46 5.46 5.46 4.62 4.62 ...
```

```
##  $ Age_s                   : num [1:88, 1] 0.833 0.833 0.833 0.833 0.833 ...
##  $ MSI_s                   : num [1:88, 1] 1.09 1.09 1.09 1.09 1.09 ...
##  $ WPQ                     : num  4.07 3.93 4.04 4.11 5 5.52 5.19 5.48 4.81 4 ...
##  $ MPQS                    : num  2.8 3.2 3 2 4 5.2 4.6 4.8 4.8 4 ...
##  $ MPQP                    : num  3.4 3 2.8 2.8 4.2 6 5.2 6 3.8 4.4 ...
##  $ log_response0M_procustus: num  0.287 -0.1776 -0.0914 -0.3584 -0.8199 ...
##  $ log_response1M_procustus: num  -0.585 -2.777 -1.983 NA NA ...
##  $ log_response0M_sparc    : num  2.38 2.38 2.36 2.38 2.39 ...
##  $ log_response1M_sparc    : num  NA NA NA NA -1.27 ...
##  $ difficulty_s            : num [1:88, 1] -0.3716 0.6817 -0.0052 0.5443 0.8649 ...
##  $ responseTSM_procustus   : num  0.2288 0.0186 0.1491 -0.0452 -0.6099 ...
```

```
head(Data)
```

```
##   participant condition trial response1M_procustus response0M_procustus
## 1           1         1     1           0.55729222            1.3323577
## 2           1         1     2           0.06221165            0.8372771
## 3           1         1     3           0.13760954            0.9126750
## 4           1         1     4          -0.07628445            0.6987810
## 5           1         2     1          -0.05685261            0.4404796
## 6           1         2     2          -0.08391585            0.4134164
##   response1M_sparc response0M_sparc Age  MSI     Age_s    MSI_s  WPQ MPQS MPQP
## 1      -0.04573307         10.78419  23 5.46 0.8330441 1.093609 4.07  2.8  3.4
## 2      -0.05117022         10.77875  23 5.46 0.8330441 1.093609 3.93  3.2  3.0
## 3      -0.20521361         10.62471  23 5.46 0.8330441 1.093609 4.04  3.0  2.8
## 4      -0.04809716         10.78182  23 5.46 0.8330441 1.093609 4.11  2.0  2.8
## 5       0.28026266         10.92805  23 5.46 0.8330441 1.093609 5.00  4.0  4.2
## 6       0.18210567         10.82989  23 5.46 0.8330441 1.093609 5.52  5.2  6.0
##   log_response0M_procustus log_response1M_procustus log_response0M_sparc
## 1               0.28695008               -0.5846655             2.378081
## 2              -0.17760015               -2.7772129             2.377577
## 3              -0.09137541               -1.9833351             2.363182
## 4              -0.35841783                       NA             2.377862
## 5              -0.81989104                       NA             2.391333
## 6              -0.88329993                       NA             2.382310
##   log_response1M_sparc difficulty_s responseTSM_procustus
## 1                   NA -0.371561179            0.22879179
## 2                   NA  0.681715889            0.01864679
## 3                   NA -0.005203938            0.14912114
## 4                   NA  0.544331924           -0.04518545
## 5            -1.272028  0.864894510           -0.60994975
## 6            -1.703168  0.086385372           -0.72281118
```

```
summary(Data)
```

```
##   participant condition trial  response1M_procustus response0M_procustus
##  1      : 8   1:44      1:22   Min.   :-0.282306    Min.   :0.3407
##  2      : 8   2:44      2:22   1st Qu.:-0.099844    1st Qu.:0.5068
##  3      : 8             3:22   Median : 0.004495    Median :0.6039
##  4      : 8             4:22   Mean   : 0.053519    Mean   :0.6754
##  5      : 8                    3rd Qu.: 0.096939    3rd Qu.:0.8021
##  6      : 8                    Max.   : 1.236650    Max.   :2.0117
##  (Other):40
```

```
##  response1M_sparc    response0M_sparc      Age             MSI
##  Min.   :-0.763675   Min.   : 9.884   Min.   :18.00   Min.   :4.150
##  1st Qu.:-0.089862   1st Qu.:10.483   1st Qu.:19.00   1st Qu.:4.620
##  Median :-0.007623   Median :10.674   Median :21.00   Median :5.000
##  Mean   :-0.022362   Mean   :10.628   Mean   :21.18   Mean   :4.923
##  3rd Qu.: 0.098376   3rd Qu.:10.791   3rd Qu.:23.00   3rd Qu.:5.460
##  Max.   : 0.364138   Max.   :11.077   Max.   :25.00   Max.   :5.540
##
##        Age_s.V1            MSI_s.V1           WPQ             MPQS
##  Min.   :-1.4578272   Min.   :-1.5740006   Min.   :3.850   Min.   :1.000
##  1st Qu.:-0.9996530   1st Qu.:-0.6169194   1st Qu.:4.440   1st Qu.:2.600
##  Median :-0.0833044   Median : 0.1568910   Median :4.910   Median :3.200
##  Mean   : 0.0000000   Mean   : 0.0000000   Mean   :4.891   Mean   :3.151
##  3rd Qu.: 0.8330441   3rd Qu.: 1.0936088   3rd Qu.:5.260   3rd Qu.:3.800
##  Max.   : 1.7493927   Max.   : 1.2565162   Max.   :6.220   Max.   :5.200
##                                            NA's   :2       NA's   :2
##       MPQP       log_response0M_procustus log_response1M_procustus
##  Min.   :2.000   Min.   :-1.0769          Min.   :-6.2412
##  1st Qu.:3.400   1st Qu.:-0.6797          1st Qu.:-3.3345
##  Median :4.200   Median :-0.5044          Median :-2.3485
##  Mean   :3.991   Mean   :-0.4601          Mean   :-2.4126
##  3rd Qu.:4.600   3rd Qu.:-0.2205          3rd Qu.:-1.4438
##  Max.   :6.000   Max.   : 0.6990          Max.   : 0.2124
##  NA's   :2                                NA's   :43
##  log_response0M_sparc log_response1M_sparc   difficulty_s.V1
##  Min.   :2.291        Min.   :-5.356       Min.   :-1.9285795
##  1st Qu.:2.350        1st Qu.:-2.644       1st Qu.:-0.7951617
##  Median :2.368        Median :-2.261       Median :-0.0738959
##  Mean   :2.363        Mean   :-2.416       Mean   : 0.0000000
##  3rd Qu.:2.379        3rd Qu.:-1.712       3rd Qu.: 0.7847539
##  Max.   :2.405        Max.   :-1.010       Max.   : 2.1929395
##                       NA's   :46
##  responseTSM_procustus
##  Min.   :-0.92818
##  1st Qu.:-0.37454
##  Median :-0.18053
##  Mean   :-0.18441
##  3rd Qu.: 0.01902
##  Max.   : 0.69898
##
```

It is of interest to show the differences between non-calibrated and calibrated metrics, all obtained by taking the median of the data points (summarizing time).

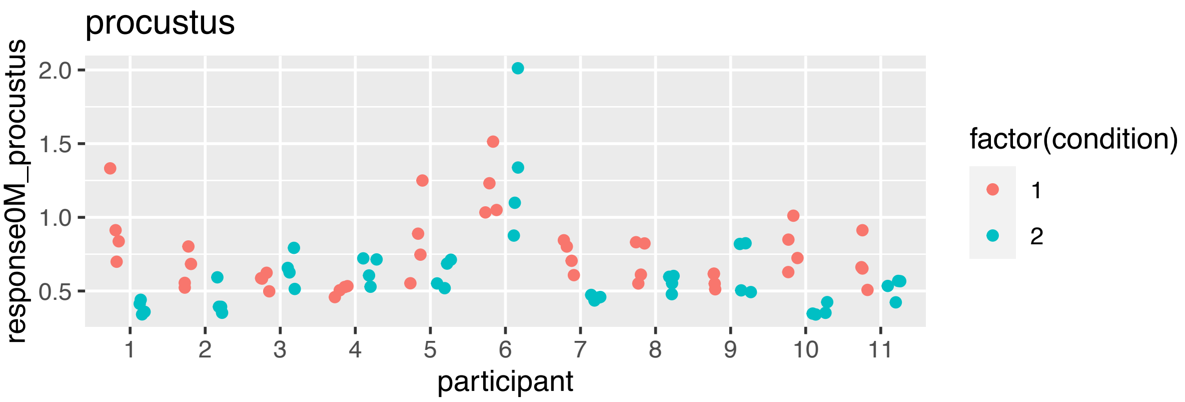
procustus
response0M_procustus
2.0
1.5
1.0
0.5
1
2
3
4
5
6
7
8
9
10
11
participant
factor(condition)
1
2

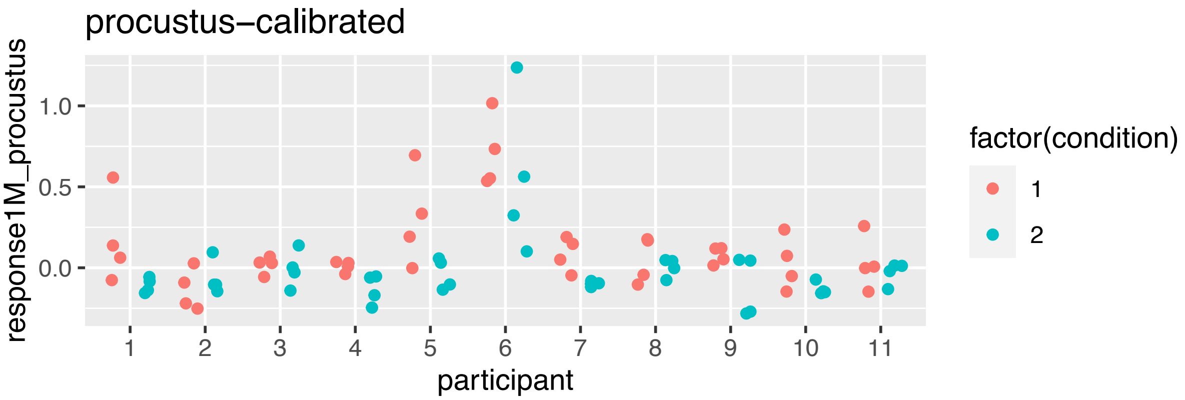
procustus−calibrated
response1M_procustus
1.0
0.5
0.0
1
2
3
4
5
6
7
8
9
10
11
participant
factor(condition)
1
2

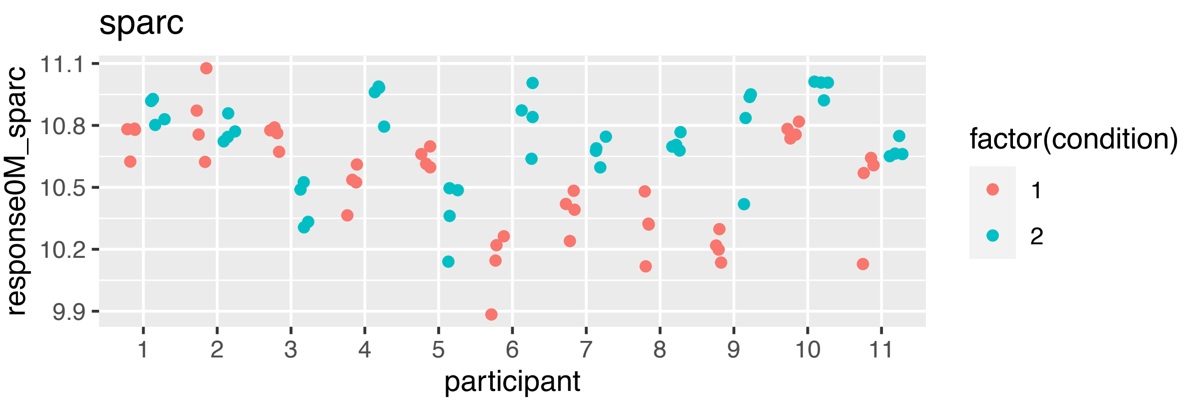

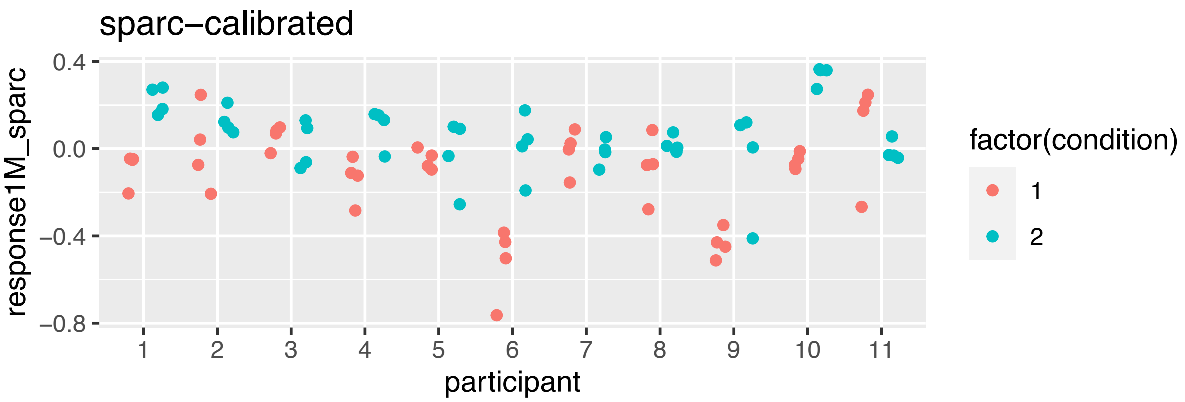

Here we show the answers to 3 presence questionnaires and 1 question about perceived difficulty.

```
## Warning: Removed 2 rows containing missing values (‘geom_point()‘).
## Removed 2 rows containing missing values (‘geom_point()‘).
```

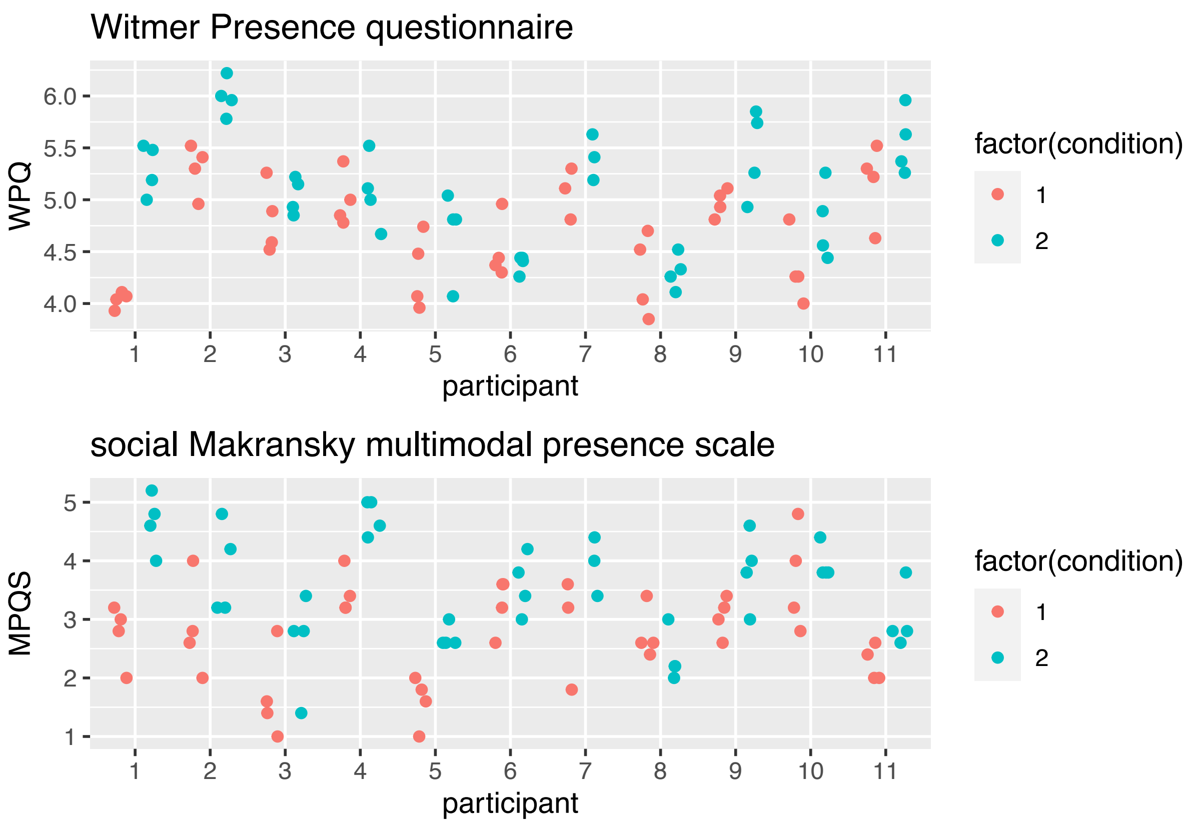


```
## Warning: Removed 2 rows containing missing values (‘geom_point()‘).
```

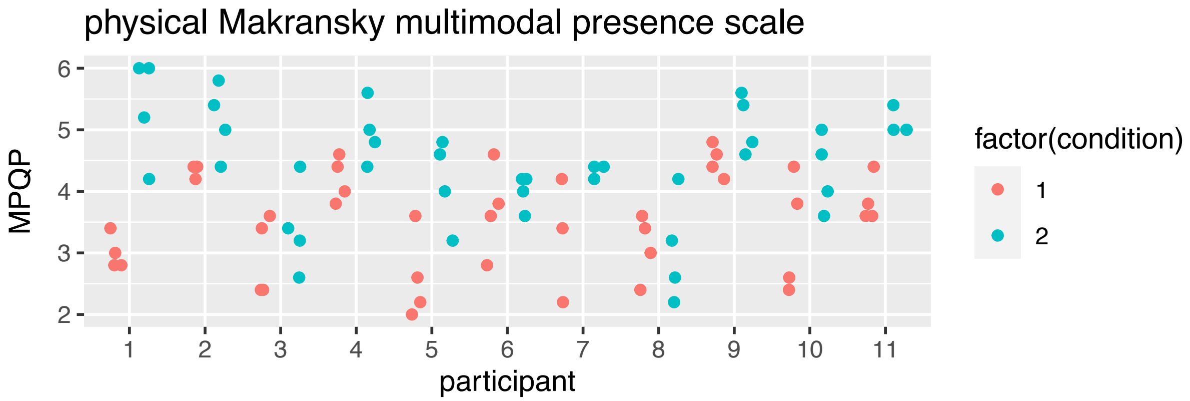

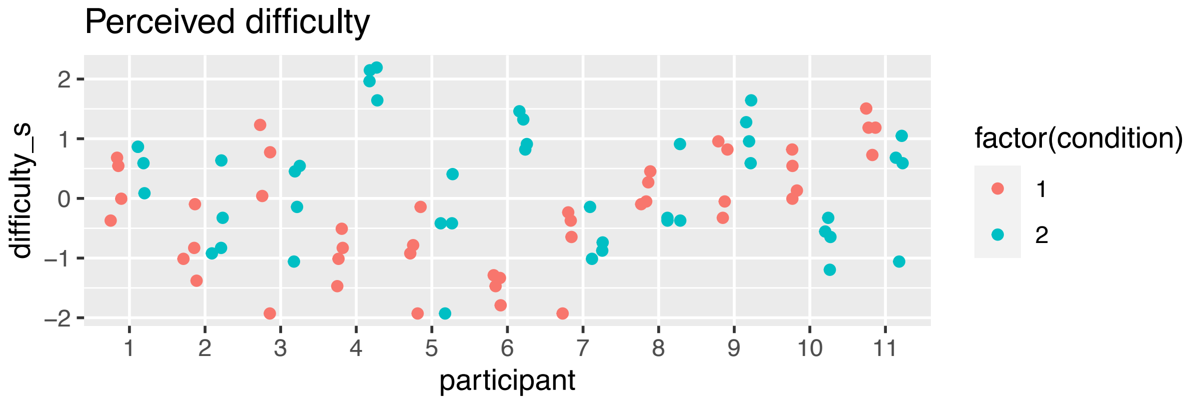

The original data of the metrics procustus and sparc are time series. Here we show a more refined approach for obtaining summarizing data of time series, based on smoothing over time using the R-packages `mgcv` and the function `gam()`. These data are extracted using spline smoothing and only the offsets of the smooths are retained, representing the time series means (TSM). figure 4 shows the smooths for the performance of participant 9, in condition 1 (2D), trials 1 to 4, belonging to group 11 (first violin, first piece).

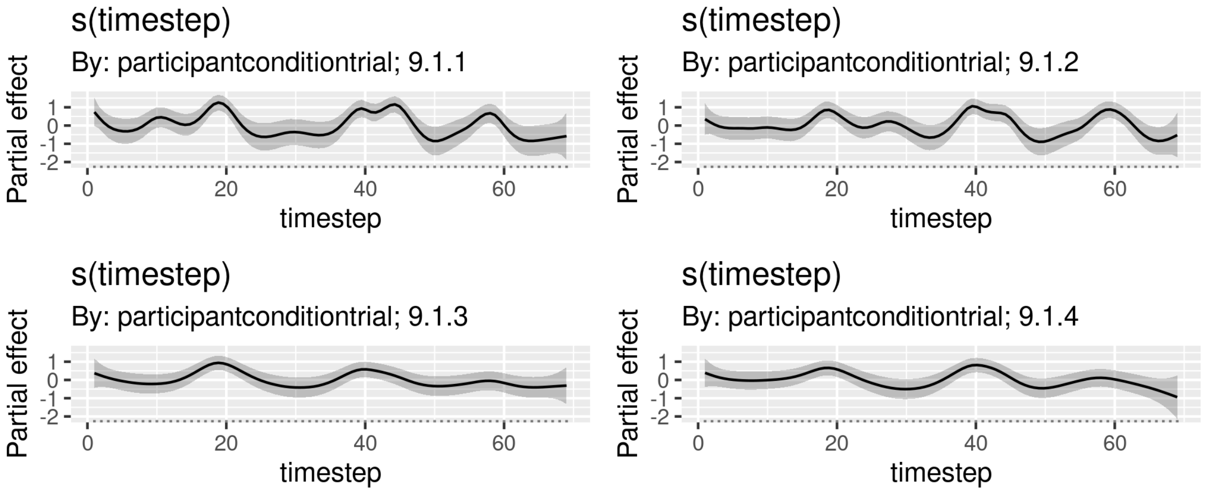

The offsets (called: TSMs) of these smooths are:

```
## participantconditiontrial9.1.1
##                     -0.2689621
```

```
## participantconditiontrial9.1.2
##                      -0.2147609

## participantconditiontrial9.1.3
##                      -0.4157548

## participantconditiontrial9.1.4
##                      -0.3686378
```

When apply this approach to all participants, conditions and trials, we obtain values that can be compared with the values obtaind by just taking the median values over time. Accordingly, the figure (labelled Figure 4) shows the correlation of TSM with 0M and 1M.

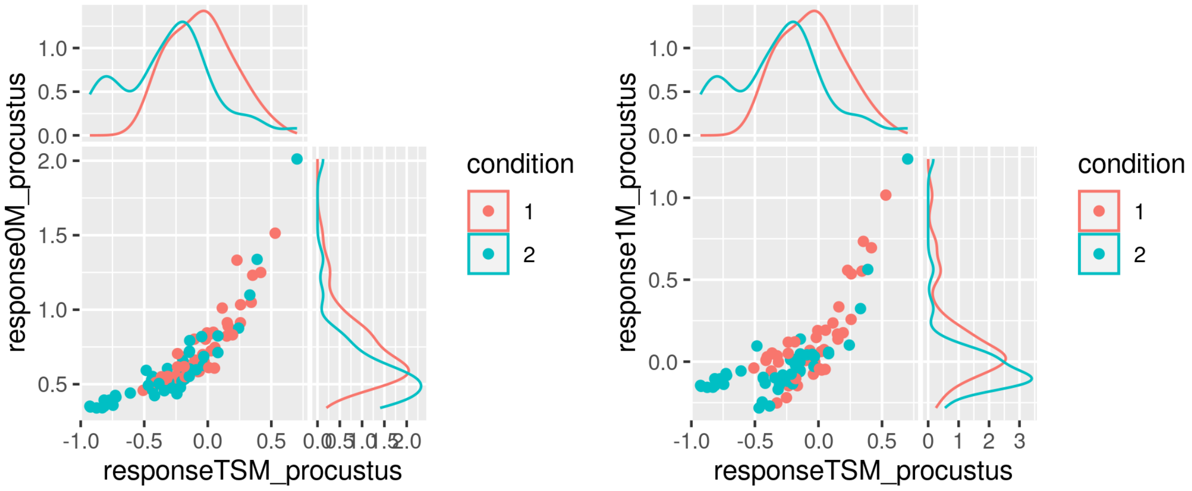

Figure 4: Hypothesis testing

Regression modelling

The second box in the workflow of Figure 1 is about regression modelling. We tested several models but we ended up with four basic models, model_1 and model_2 for the metric workflow, and model_3 and model_4 for the questionnaire workflow. The syntax of the models (here in R package `brms` format) is very similar.

- Model_1: response ~ 0 + condition + (1 | condition:participant + condition:trial))
- Model_2: response ~ 0 + condition*difficulty + (1 + difficulty | condition:participant + condition:trial))
- Model_3: response ~ 0 + condition + (1 | condition:participant + condition:trial))
- Model_4: response ~ 0 + condition*procustus + (1 + procustus | condition:participant + condition:trial))

where:

- `response` is either the procustus of sparc metrics (giving us 2 different models for model_1),
- `condition` is either a 2D or 3D rendering of the visual scene,
- `difficulty` is the perceived difficulty of the task,
- `procustus` is the procustus metric of the task,
- `trial` is the participant's session.

In model_1 and model_2 a `skew_normal` link function is used, in model_3 and model_4 a `gaussian` link function is used. Note further that `participant` and `trial` are exchangeable variables. The advantage of the mixed model is that these variables can be modelled as instances of distributions at a higher hierarchical level. Accordingly, each participant, being exchangeable, is drawn from a normal distribution whose sd is estimated by the model. Same for trial. This modelling approach prevents overfitting by shrinking the instances of the group-level variables `participant` and `trial` towards the means of the respective group-level. Since `condition` has only two levels, we keep it as population variable. Group-level effects of `trial` are used later in a contrast analysis. Another way of looking at this regression is that it captures variability that is related to `participant` and `trial`, leaving a more "pure" variability of interest to `condition`.

We run the models on a 48 dual core machine (at Ghent University, IPEM), using the R package `brms`. We take 5000 warmups and 40000 iterations, with an adapt_delta = 0.995 and max_treedepth = 12, 4 chains, and 24 threads. The large amount of iterations was needed in view of a stable Bayes factor test in the R package `parameters`.

Analysis

We then proceed with the analysis in 3 parts (Figure 1).

- 1. Comparison. We do a comparison of two models (model_1 = without `difficulty`, model_2 = with `difficulty`) using the Bayes-factor test (using `bayes_factor()`). Running ahead, we found that none of the model_2 turn are any better than model_1.
- 2. Diagnostics. We use `pp_check()` for a global retrodiction check and `mcmc_plot()` for an overview of the posterior distributions of parameters, we also run a `bayes_R2()` to get an estimate of the variances, and `parameters()` in order to get a summary of the model.
- 3. Contrasts. We code trials as factors. Alternatively, we could have chosen a longitudinal approach coding trial as `integer` (rather than factor) but we thought that a factor approach was more appropriate given the fact that order was relevant, instead of the exact time between the sessions. We report contrast testing both as table and as plot.

PART 1

Part 1 of this analysis is related to the procustus and sparc metrics and hypothesis 1 and 2.

1. Comparison

We tested the models for procustus and sparc and report here the log of the Bayes factor.

Bayes-factor non-calibrated models

```
## [1] "Bayes factor in favor of model_1 over model_2 (procutus0M):  11.0734169658963"
```

```
## [1] "Bayes factor in favor of model_1 over model_2 (sparc0M):  594.905974803006"
```

Bayes-factor calibrated models

```
## [1] "Bayes factor in favor of model_1 over model_2 (procustus1M):  4.07095152389583"
```

```
## [1] "Bayes factor in favor of model_1 over model_2 (sparc1M):  5.74615655005747"
```

Bayes-factor TSM models

```
## [1] "Bayes factor in favor of model_1 over model_2 (procustusTMS):  5.01452225625957"
```

We conclude that there is strong evidence for model_1 (i.e., without `difficulty`).

2. Diagnostics

We show the diagnostics both for the procustus and sparc model_1:

- the model_1 formula,
- the Bayes_R2 analysis,
- the model parameters,
- the posterior prediction check (pp_check) next to the plot of fixed parameters (i.e. `condition`)

Diagnostics for non-calibrated models

```
## [[1]]
## response0M_procustus ~ 0 + condition + (1 | condition:participant) + (1 | condition:trial)
##
## [[2]]
```

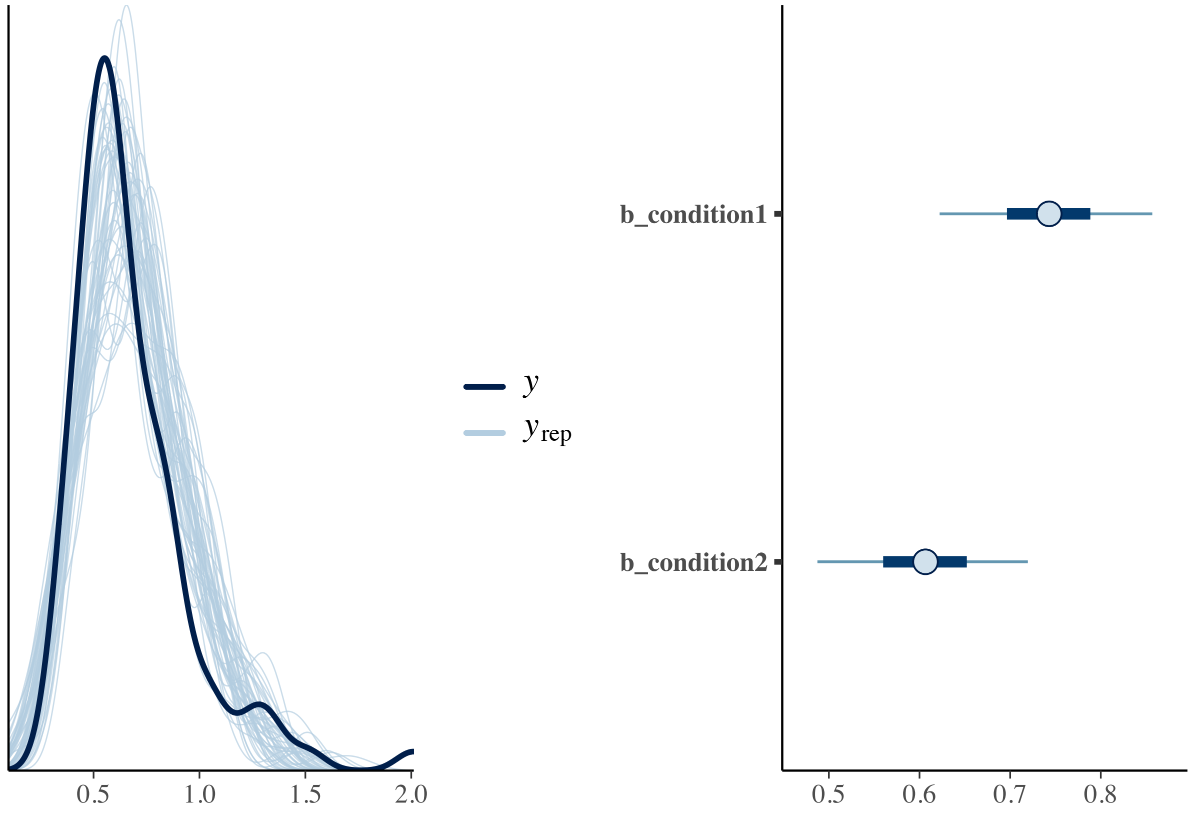


```
##
## [[3]]
##     R2   SD   CI CI_low CI_high CI_method   Component         Effectsize
## 1 0.57 0.07 0.95   0.41    0.69       HDI conditional Bayesian R-squared
## 2 0.06 0.07 0.95   0.00    0.22       HDI    marginal Bayesian R-squared
##
## [[4]]
##                          Parameter Effects   Component Mean   CI CI_low
## 1                     b_condition1   fixed conditional 0.74 0.95   0.59
## 2                     b_condition2   fixed conditional 0.61 0.95   0.46
## 3 sd_condition:participant__Intercept  random conditional 0.18 0.95   0.12
## 4       sd_condition:trial__Intercept  random conditional 0.08 0.95   0.02
## 5                            sigma   fixed       sigma 0.15 0.95   0.12
##   CI_high pd log_BF Rhat      ESS
## 1    0.88  1  11.66    1 35327.68
## 2    0.74  1  11.42    1 36961.64
## 3    0.27  1  11.20    1 33076.10
## 4    0.18  1  -0.64    1 39603.18
## 5    0.18  1  45.94    1 55830.83

## [[1]]
## responseOM_sparc ~ 0 + condition + (1 | condition:participant) + (1 | condition:trial)
##
## [[2]]
```

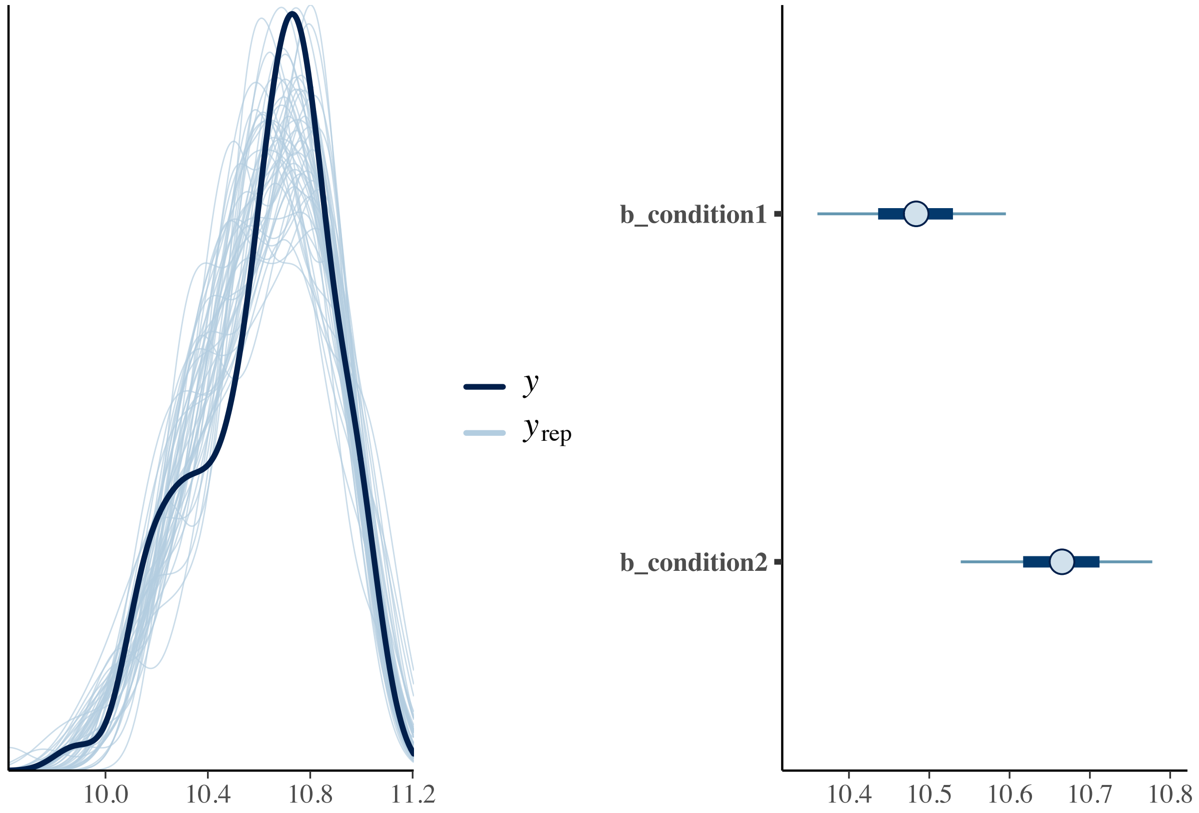


```
##
## [[3]]
##     R2   SD   CI CI_low CI_high CI_method   Component         Effectsize
## 1 0.77 0.02 0.95   0.71    0.81       HDI conditional Bayesian R-squared
## 2 0.13 0.12 0.95   0.00    0.33       HDI    marginal Bayesian R-squared
##
## [[4]]
##                          Parameter Effects   Component  Mean   CI CI_low
## 1                     b_condition1   fixed conditional 10.48 0.95  10.33
## 2                     b_condition2   fixed conditional 10.66 0.95  10.51
## 3 sd_condition:participant__Intercept  random conditional  0.23 0.95   0.16
## 4       sd_condition:trial__Intercept  random conditional  0.01 0.95   0.00
## 5                            sigma   fixed       sigma  0.12 0.95   0.10
##   CI_high pd log_BF Rhat      ESS
## 1   10.62  1 232.00    1 25985.59
## 2   10.80  1  99.34    1 24745.93
## 3    0.33  1  22.66    1 28850.74
## 4    0.05  1  -5.73    1 83617.14
## 5    0.15  1  39.49    1 71133.38
```

Diagnostics for calibrated models

```
## [[1]]
## response1M_procustus ~ 0 + condition + (1 | condition:participant) + (1 | condition:trial)
##
```

```
## [[2]]
```

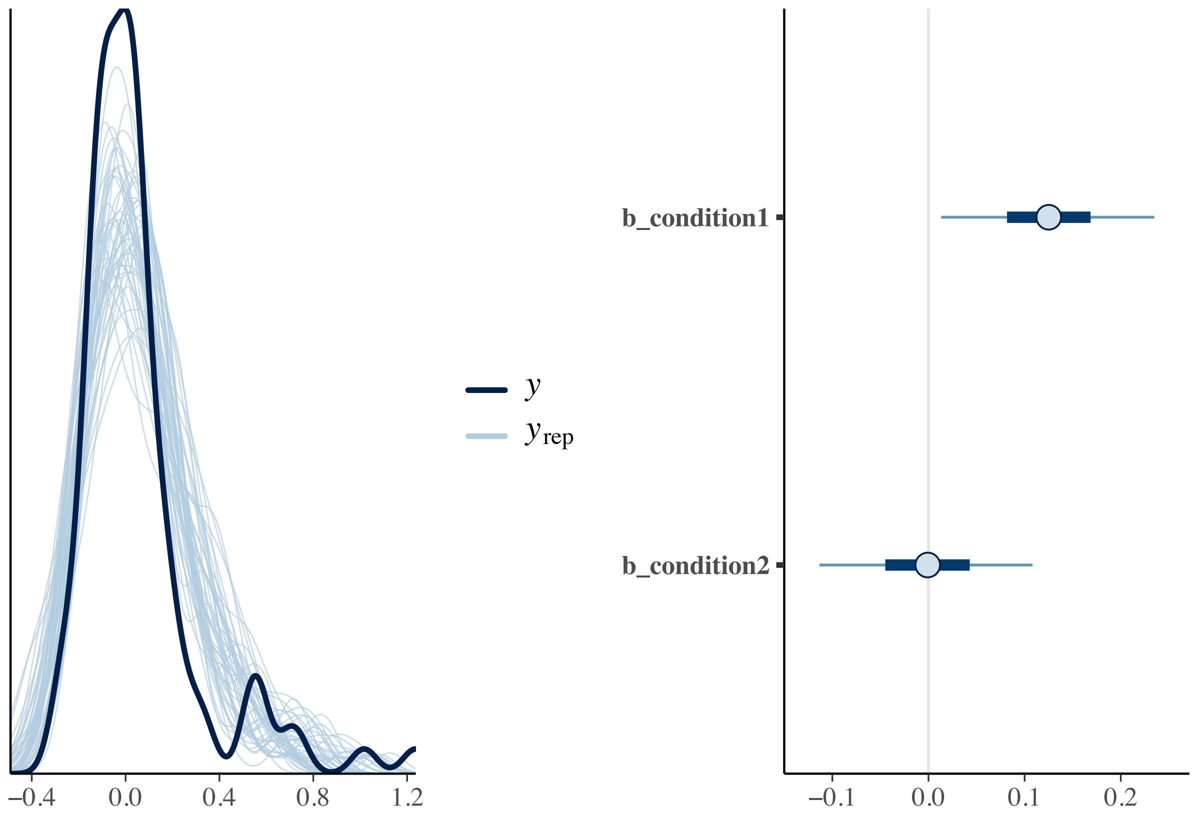


```
##
## [[3]]
##     R2   SD   CI CI_low CI_high CI_method   Component         Effectsize
## 1 0.53 0.08 0.95   0.33    0.67       HDI conditional Bayesian R-squared
## 2 0.06 0.07 0.95   0.00    0.23       HDI    marginal Bayesian R-squared
##
## [[4]]
##                            Parameter Effects   Component Mean   CI CI_low
## 1                       b_condition1   fixed conditional 0.12 0.95  -0.01
## 2                       b_condition2   fixed conditional 0.00 0.95  -0.14
## 3 sd_condition:participant__Intercept  random conditional 0.16 0.95   0.10
## 4       sd_condition:trial__Intercept  random conditional 0.08 0.95   0.03
## 5                              sigma   fixed       sigma 0.15 0.95   0.12
##   CI_high   pd log_BF Rhat      ESS
## 1    0.26 0.96   0.46    1 34938.01
## 2    0.13 0.50  -1.36    1 34492.17
## 3    0.24 1.00   6.19    1 32387.70
## 4    0.19 1.00  -0.10    1 38051.45
## 5    0.19 1.00  43.31    1 50570.28
```

```
## [[1]]
## response1M_sparc ~ 0 + condition + (1 | condition:participant) + (1 | condition:trial)
##
## [[2]]
```

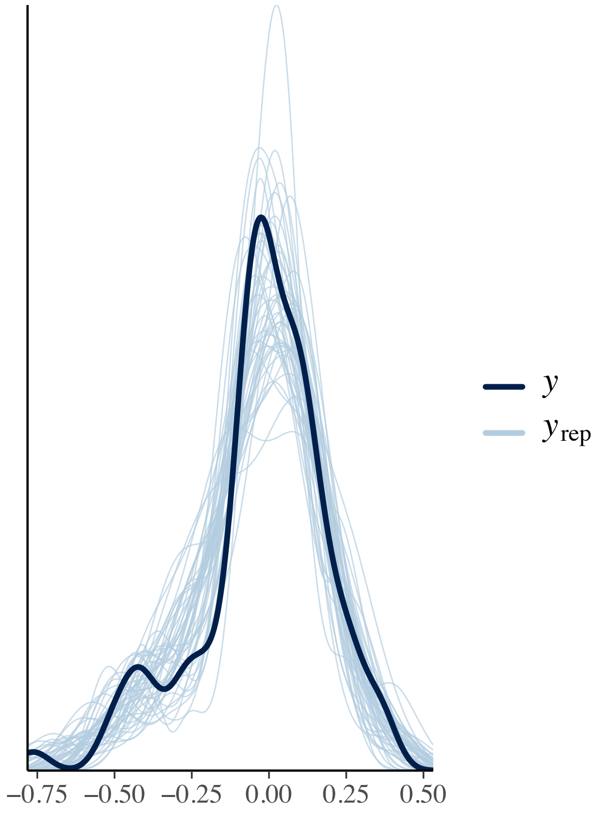

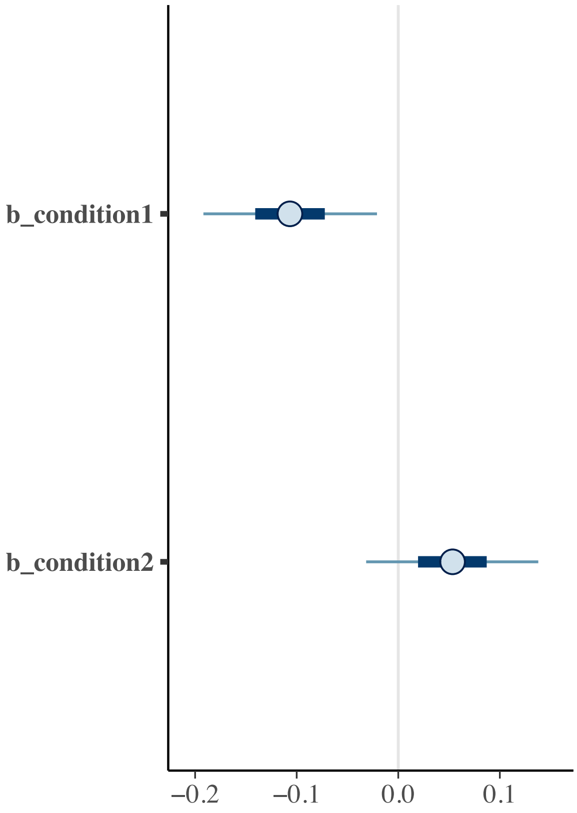


```
##
## [[3]]
##     R2   SD   CI CI_low CI_high CI_method   Component         Effectsize
## 1 0.65 0.04 0.95   0.55    0.72       HDI conditional Bayesian R-squared
## 2 0.15 0.11 0.95   0.00    0.34       HDI    marginal Bayesian R-squared
##
## [[4]]
##                              Parameter Effects   Component  Mean   CI CI_low
## 1                         b_condition1   fixed conditional -0.11 0.95  -0.21
## 2                         b_condition2   fixed conditional  0.05 0.95  -0.05
## 3 sd_condition:participant__Intercept  random conditional  0.16 0.95   0.11
## 4       sd_condition:trial__Intercept  random conditional  0.01 0.95   0.00
## 5                                sigma   fixed       sigma  0.12 0.95   0.10
##   CI_high   pd log_BF Rhat      ESS
## 1    0.00 0.98   0.56    1 30831.21
## 2    0.16 0.85  -1.02    1 30072.63
## 3    0.23 1.00  15.42    1 34790.49
## 4    0.05 1.00  -4.47    1 97322.66
## 5    0.14 1.00  38.87    1 87163.39
```

Diagnostics for TSM

```
## [[1]]
## responseTSM_procustus ~ 0 + condition + (1 | condition:participant) + (1 | condition:trial)
##
```

```
## [[2]]
```

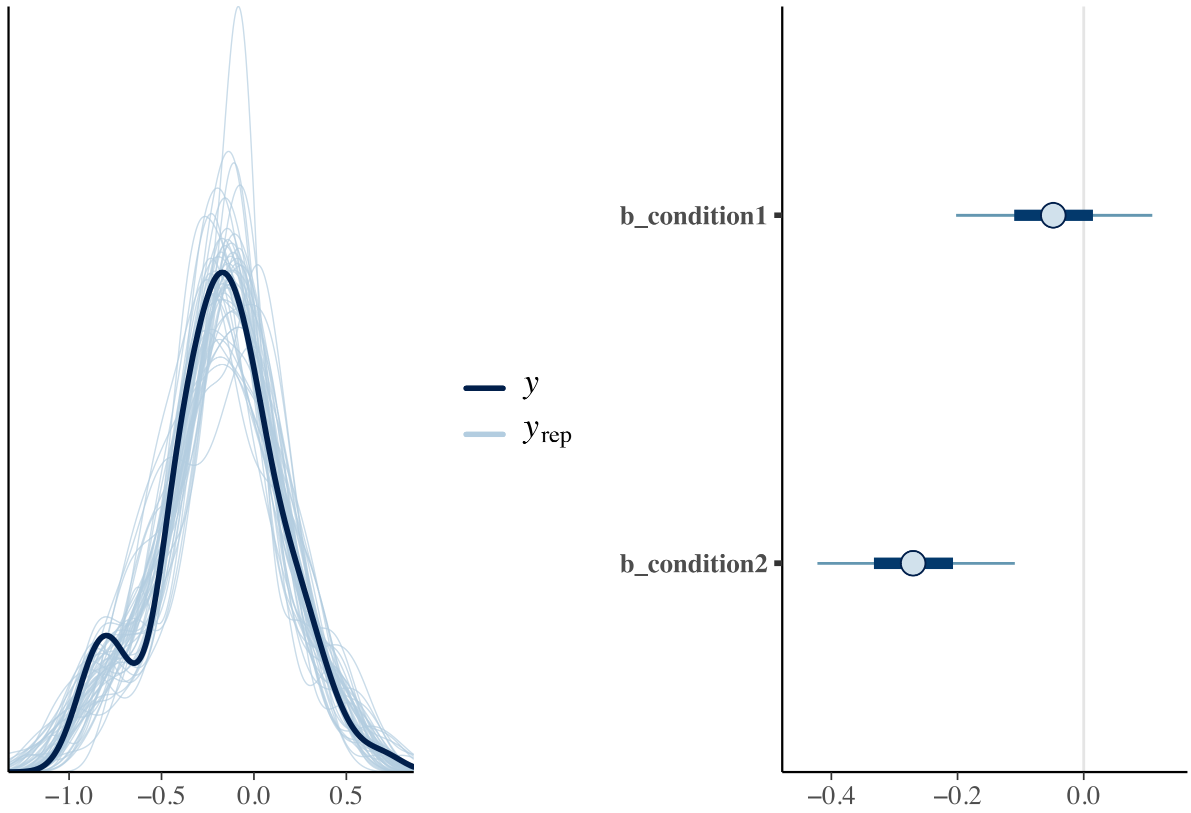


```
##
## [[3]]
##     R2   SD   CI CI_low CI_high CI_method   Component         Effectsize
## 1 0.81 0.03 0.95   0.75    0.85       HDI conditional Bayesian R-squared
## 2 0.12 0.11 0.95   0.00    0.32       HDI    marginal Bayesian R-squared
##
## [[4]]
##                             Parameter Effects   Component  Mean   CI CI_low
## 1                        b_condition1   fixed conditional -0.05 0.95  -0.23
## 2                        b_condition2   fixed conditional -0.27 0.95  -0.45
## 3 sd_condition:participant__Intercept  random conditional  0.30 0.95   0.21
## 4       sd_condition:trial__Intercept  random conditional  0.07 0.95   0.01
## 5                               sigma   fixed       sigma  0.15 0.95   0.13
##   CI_high  pd log_BF Rhat      ESS
## 1    0.14 0.7  -0.85    1 25226.22
## 2   -0.07 1.0   2.62    1 26099.77
## 3    0.43 1.0  23.69    1 25498.70
## 4    0.18 1.0  -2.10    1 38376.41
## 5    0.18 1.0  38.48    1 66260.63
```

3. Contrasts

We show the contrasts both for the procustus model_1 and the sparc model_1. See the paper for a discussion about the contrast results. The `Label` is coded as follows: c stands for condition, c12 for a contrast of condition 1 and condition 2. t stands for trial, t12 stands for a contrast of trial 1 and trial 2. Accordingly, c1t12 stands for a contrast of trial 1 an trial 2 in condition 1. c12t1 stands for a contrast of trial 1 in condiition 1 versus condition 2.

Contrasts for non-calibrated models

```r
load(file = "Results/hypothesis_test_procustusOM.RData")
load(file = "Results/hypothesis_test_sparcOM.RData")

hypothesis_test_procustusOM[[1]][,1]
```

```
##  [1] "(b_condition1)-(b_condition2) < 0"
##  [2] "(r_condition:trial[1_1,Intercept])-(r_condition:trial[1_2,Intercept]) > 0"
##  [3] "(r_condition:trial[1_1,Intercept])-(r_condition:trial[1_3,Intercept]) > 0"
##  [4] "(r_condition:trial[1_1,Intercept])-(r_condition:trial[1_4,Intercept]) > 0"
##  [5] "(r_condition:trial[1_2,Intercept])-(r_condition:trial[1_3,Intercept]) > 0"
##  [6] "(r_condition:trial[1_2,Intercept])-(r_condition:trial[1_4,Intercept]) > 0"
##  [7] "(r_condition:trial[1_3,Intercept])-(r_condition:trial[1_4,Intercept]) > 0"
##  [8] "(r_condition:trial[2_1,Intercept])-(r_condition:trial[2_2,Intercept]) > 0"
##  [9] "(r_condition:trial[2_1,Intercept])-(r_condition:trial[2_3,Intercept]) > 0"
## [10] "(r_condition:trial[2_1,Intercept])-(r_condition:trial[2_4,Intercept]) > 0"
## [11] "(r_condition:trial[2_2,Intercept])-(r_condition:trial[2_3,Intercept]) > 0"
## [12] "(r_condition:trial[2_2,Intercept])-(r_condition:trial[2_4,Intercept]) > 0"
## [13] "(r_condition:trial[2_3,Intercept])-(r_condition:trial[2_4,Intercept]) > 0"
## [14] "(b_condition1+r_condition:trial[1_1,Intercept])-(b_condition2+r_condition:trial[2_1,Intercept]
## [15] "(b_condition1+r_condition:trial[1_2,Intercept])-(b_condition2+r_condition:trial[2_2,Intercept]
## [16] "(b_condition1+r_condition:trial[1_3,Intercept])-(b_condition2+r_condition:trial[2_3,Intercept]
## [17] "(b_condition1+r_condition:trial[1_4,Intercept])-(b_condition2+r_condition:trial[2_4,Intercept]
```

```r
hypothesis_test_procustusOM[[1]][,-1]
```

```
##     Label Estimate CI.Lower CI.Upper Post.Prob Star
## t1    c12     0.14    -0.03     0.30      0.08
## t2  c1t12     0.05    -0.03     0.14      0.84
## t3  c1t13     0.12     0.02     0.21      0.98    *
## t4  c1t14     0.15     0.05     0.25      0.99    *
## t5  c1t23     0.07    -0.01     0.15      0.92
## t6  c1t24     0.10     0.02     0.19      0.98    *
## t7  c1t34     0.04    -0.04     0.11      0.79
## t8  c2t12     0.07    -0.02     0.15      0.90
## t9  c2t13     0.06    -0.01     0.14      0.92
## t10 c2t14     0.07     0.00     0.15      0.94
## t11 c2t23     0.00    -0.08     0.07      0.49
## t12 c2t24     0.00    -0.07     0.08      0.55
## t13 c2t34     0.01    -0.07     0.08      0.56
## t14 c12t1     0.17     0.02     0.32      0.97    *
## t15 c12t2     0.19     0.04     0.34      0.98    *
```

```
## t16 c12t3     0.12    -0.03     0.26      0.90
## t17 c12t4     0.09    -0.06     0.23      0.84
```

```
hypothesis_test_procustusOM[[2]]
```

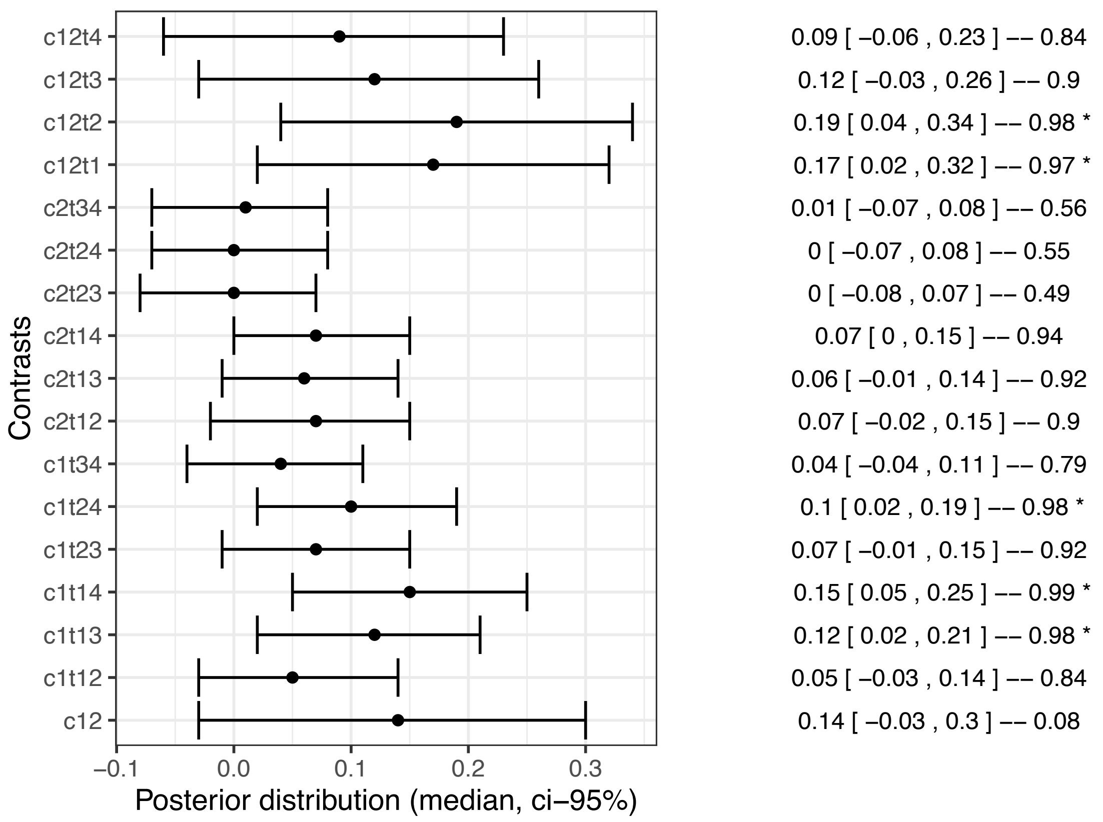


```
hypothesis_test_sparcOM[[1]][,1]
```

```
##  [1] "(b_condition1)-(b_condition2) < 0"
##  [2] "(r_condition:trial[1_1,Intercept])-(r_condition:trial[1_2,Intercept]) > 0"
##  [3] "(r_condition:trial[1_1,Intercept])-(r_condition:trial[1_3,Intercept]) > 0"
##  [4] "(r_condition:trial[1_1,Intercept])-(r_condition:trial[1_4,Intercept]) > 0"
##  [5] "(r_condition:trial[1_2,Intercept])-(r_condition:trial[1_3,Intercept]) > 0"
##  [6] "(r_condition:trial[1_2,Intercept])-(r_condition:trial[1_4,Intercept]) > 0"
##  [7] "(r_condition:trial[1_3,Intercept])-(r_condition:trial[1_4,Intercept]) > 0"
##  [8] "(r_condition:trial[2_1,Intercept])-(r_condition:trial[2_2,Intercept]) > 0"
##  [9] "(r_condition:trial[2_1,Intercept])-(r_condition:trial[2_3,Intercept]) > 0"
## [10] "(r_condition:trial[2_1,Intercept])-(r_condition:trial[2_4,Intercept]) > 0"
## [11] "(r_condition:trial[2_2,Intercept])-(r_condition:trial[2_3,Intercept]) > 0"
## [12] "(r_condition:trial[2_2,Intercept])-(r_condition:trial[2_4,Intercept]) > 0"
## [13] "(r_condition:trial[2_3,Intercept])-(r_condition:trial[2_4,Intercept]) > 0"
## [14] "(b_condition1+r_condition:trial[1_1,Intercept])-(b_condition2+r_condition:trial[2_1,Intercept]
## [15] "(b_condition1+r_condition:trial[1_2,Intercept])-(b_condition2+r_condition:trial[2_2,Intercept]
## [16] "(b_condition1+r_condition:trial[1_3,Intercept])-(b_condition2+r_condition:trial[2_3,Intercept]
## [17] "(b_condition1+r_condition:trial[1_4,Intercept])-(b_condition2+r_condition:trial[2_4,Intercept]
```

```
hypothesis_test_sparcOM[[1]][,-1]
```

```
##      Label Estimate CI.Lower CI.Upper Post.Prob Star
## t1     c12    -0.18    -0.34    -0.02      0.96    *
## t2  c1t12     0.00    -0.03     0.03      0.50
## t3  c1t13     0.00    -0.03     0.03      0.51
## t4  c1t14     0.00    -0.03     0.03      0.50
## t5  c1t23     0.00    -0.03     0.03      0.51
## t6  c1t24     0.00    -0.03     0.03      0.50
## t7  c1t34     0.00    -0.03     0.03      0.49
## t8  c2t12     0.00    -0.03     0.03      0.50
## t9  c2t13     0.00    -0.03     0.03      0.50
## t10 c2t14     0.00    -0.02     0.04      0.56
## t11 c2t23     0.00    -0.03     0.03      0.50
## t12 c2t24     0.00    -0.02     0.04      0.56
## t13 c2t34     0.00    -0.02     0.04      0.56
## t14 c12t1    -0.18    -0.35    -0.02      0.04
## t15 c12t2    -0.18    -0.35    -0.02      0.04
## t16 c12t3    -0.18    -0.35    -0.02      0.04
## t17 c12t4    -0.18    -0.34    -0.01      0.04
```

```
hypothesis_test_sparcOM[[2]]
```

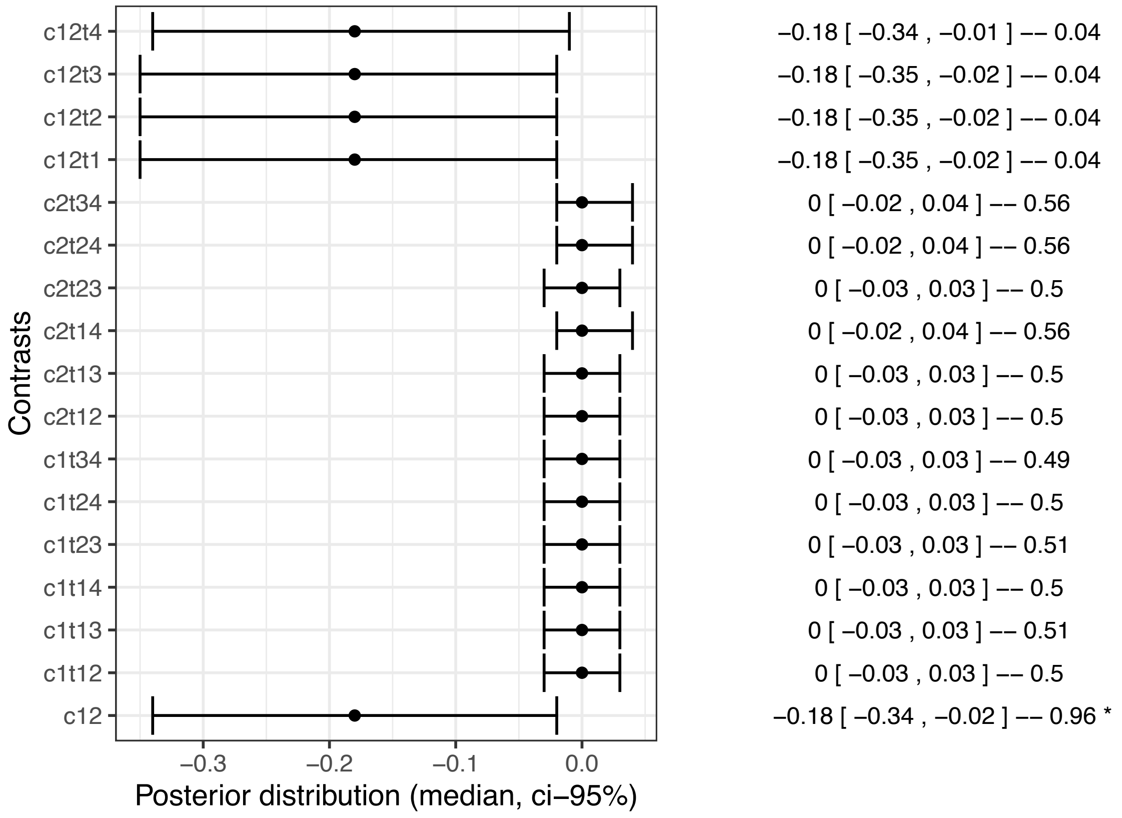

Contrasts for calibrated models

```
load(file = "Results/hypothesis_test_procustus1M.RData")
load(file = "Results/hypothesis_test_sparc1M.RData")

hypothesis_test_procustus1M[[1]][,1]
```

```
##  [1] "(b_condition1)-(b_condition2) < 0"
##  [2] "(r_condition:trial[1_1,Intercept])-(r_condition:trial[1_2,Intercept]) > 0"
##  [3] "(r_condition:trial[1_1,Intercept])-(r_condition:trial[1_3,Intercept]) > 0"
##  [4] "(r_condition:trial[1_1,Intercept])-(r_condition:trial[1_4,Intercept]) > 0"
##  [5] "(r_condition:trial[1_2,Intercept])-(r_condition:trial[1_3,Intercept]) > 0"
##  [6] "(r_condition:trial[1_2,Intercept])-(r_condition:trial[1_4,Intercept]) > 0"
##  [7] "(r_condition:trial[1_3,Intercept])-(r_condition:trial[1_4,Intercept]) > 0"
##  [8] "(r_condition:trial[2_1,Intercept])-(r_condition:trial[2_2,Intercept]) > 0"
##  [9] "(r_condition:trial[2_1,Intercept])-(r_condition:trial[2_3,Intercept]) > 0"
## [10] "(r_condition:trial[2_1,Intercept])-(r_condition:trial[2_4,Intercept]) > 0"
## [11] "(r_condition:trial[2_2,Intercept])-(r_condition:trial[2_3,Intercept]) > 0"
## [12] "(r_condition:trial[2_2,Intercept])-(r_condition:trial[2_4,Intercept]) > 0"
## [13] "(r_condition:trial[2_3,Intercept])-(r_condition:trial[2_4,Intercept]) > 0"
## [14] "(b_condition1+r_condition:trial[1_1,Intercept])-(b_condition2+r_condition:trial[2_1,Intercept]
## [15] "(b_condition1+r_condition:trial[1_2,Intercept])-(b_condition2+r_condition:trial[2_2,Intercept]
## [16] "(b_condition1+r_condition:trial[1_3,Intercept])-(b_condition2+r_condition:trial[2_3,Intercept]
## [17] "(b_condition1+r_condition:trial[1_4,Intercept])-(b_condition2+r_condition:trial[2_4,Intercept]
```

```
hypothesis_test_procustus1M[[1]][,-1]
```

```
##     Label Estimate CI.Lower CI.Upper Post.Prob Star
## t1    c12     0.13    -0.03     0.28      0.09
## t2  c1t12     0.06    -0.02     0.15      0.88
## t3  c1t13     0.13     0.03     0.22      0.99    *
## t4  c1t14     0.16     0.06     0.26      1.00    *
## t5  c1t23     0.07    -0.01     0.14      0.91
## t6  c1t24     0.10     0.02     0.19      0.98    *
## t7  c1t34     0.04    -0.04     0.12      0.80
## t8  c2t12     0.07    -0.01     0.16      0.92
## t9  c2t13     0.07    -0.01     0.15      0.93
## t10 c2t14     0.07    -0.01     0.15      0.93
## t11 c2t23     0.00    -0.08     0.08      0.48
## t12 c2t24     0.00    -0.08     0.08      0.49
## t13 c2t34     0.00    -0.08     0.08      0.51
## t14 c12t1     0.17     0.03     0.31      0.97    *
## t15 c12t2     0.18     0.04     0.32      0.98    *
## t16 c12t3     0.11    -0.03     0.25      0.90
## t17 c12t4     0.07    -0.07     0.21      0.81
```

```
hypothesis_test_procustus1M[[2]]
```

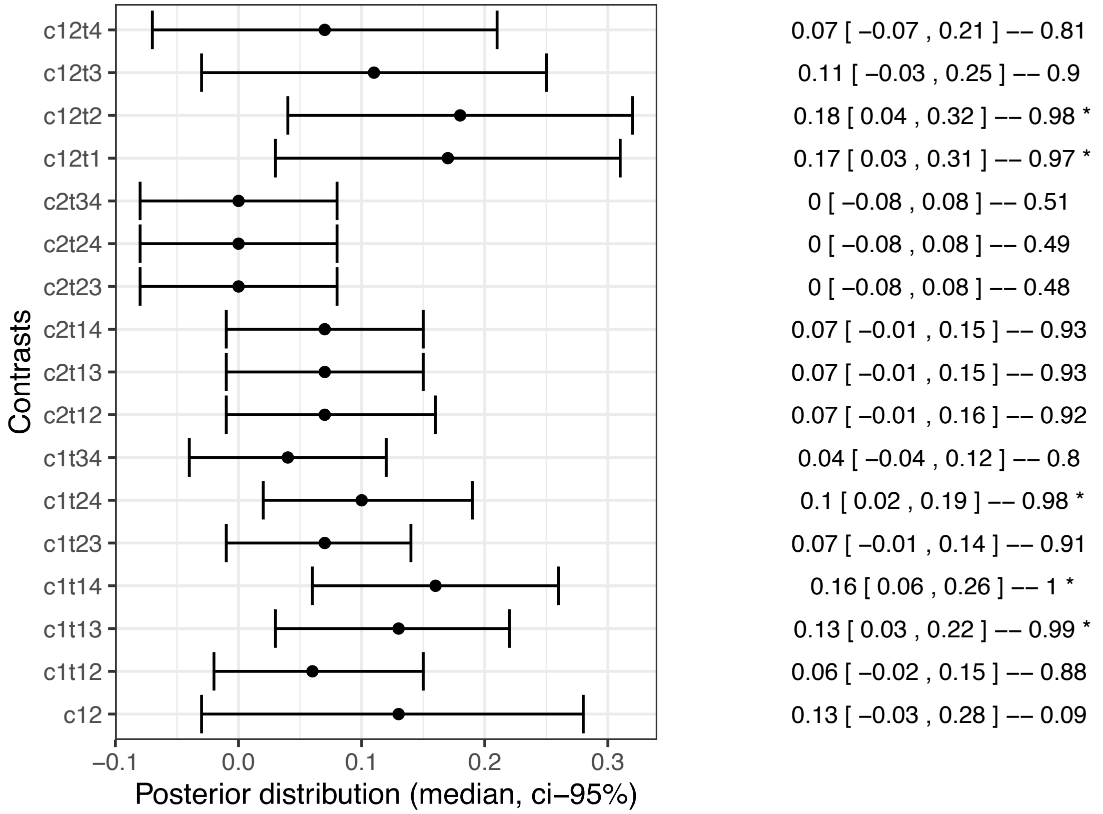


```
hypothesis_test_sparc1M[[1]][,1]
```

```
##  [1] "(b_condition1)-(b_condition2) < 0"
##  [2] "(r_condition:trial[1_1,Intercept])-(r_condition:trial[1_2,Intercept]) > 0"
##  [3] "(r_condition:trial[1_1,Intercept])-(r_condition:trial[1_3,Intercept]) > 0"
##  [4] "(r_condition:trial[1_1,Intercept])-(r_condition:trial[1_4,Intercept]) > 0"
##  [5] "(r_condition:trial[1_2,Intercept])-(r_condition:trial[1_3,Intercept]) > 0"
##  [6] "(r_condition:trial[1_2,Intercept])-(r_condition:trial[1_4,Intercept]) > 0"
##  [7] "(r_condition:trial[1_3,Intercept])-(r_condition:trial[1_4,Intercept]) > 0"
##  [8] "(r_condition:trial[2_1,Intercept])-(r_condition:trial[2_2,Intercept]) > 0"
##  [9] "(r_condition:trial[2_1,Intercept])-(r_condition:trial[2_3,Intercept]) > 0"
## [10] "(r_condition:trial[2_1,Intercept])-(r_condition:trial[2_4,Intercept]) > 0"
## [11] "(r_condition:trial[2_2,Intercept])-(r_condition:trial[2_3,Intercept]) > 0"
## [12] "(r_condition:trial[2_2,Intercept])-(r_condition:trial[2_4,Intercept]) > 0"
## [13] "(r_condition:trial[2_3,Intercept])-(r_condition:trial[2_4,Intercept]) > 0"
## [14] "(b_condition1+r_condition:trial[1_1,Intercept])-(b_condition2+r_condition:trial[2_1,Intercept]
## [15] "(b_condition1+r_condition:trial[1_2,Intercept])-(b_condition2+r_condition:trial[2_2,Intercept]
## [16] "(b_condition1+r_condition:trial[1_3,Intercept])-(b_condition2+r_condition:trial[2_3,Intercept]
## [17] "(b_condition1+r_condition:trial[1_4,Intercept])-(b_condition2+r_condition:trial[2_4,Intercept]
```

```
hypothesis_test_sparc1M[[1]][,-1]
```

```
##     Label Estimate CI.Lower CI.Upper Post.Prob Star
## t1    c12    -0.16    -0.28    -0.04      0.98    *
```

```
## t2  c1t12     0.00    -0.03     0.03      0.49
## t3  c1t13     0.00    -0.03     0.03      0.49
## t4  c1t14     0.00    -0.03     0.03      0.50
## t5  c1t23     0.00    -0.03     0.03      0.50
## t6  c1t24     0.00    -0.03     0.03      0.51
## t7  c1t34     0.00    -0.03     0.03      0.50
## t8  c2t12     0.00    -0.03     0.03      0.50
## t9  c2t13     0.00    -0.03     0.03      0.51
## t10 c2t14     0.00    -0.02     0.04      0.58
## t11 c2t23     0.00    -0.03     0.03      0.51
## t12 c2t24     0.00    -0.02     0.04      0.58
## t13 c2t34     0.00    -0.02     0.04      0.57
## t14 c12t1    -0.16    -0.28    -0.04      0.02
## t15 c12t2    -0.16    -0.28    -0.04      0.02
## t16 c12t3    -0.16    -0.28    -0.04      0.02
## t17 c12t4    -0.16    -0.28    -0.04      0.02
```

```
hypothesis_test_sparc1M[[2]]
```

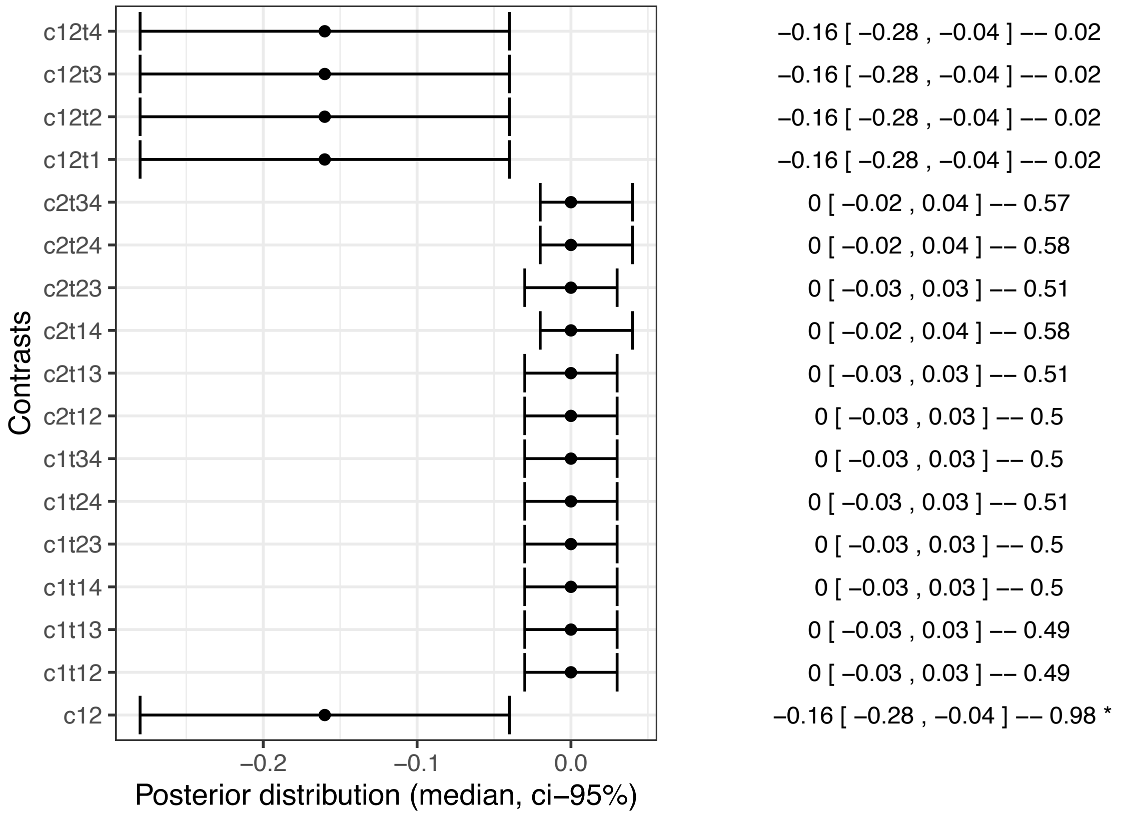

Added view on trial distributions

In Figure 5 and 6 we show the trial distributions drawn from the calibrated model for the procustus and sparc metrics. The graphs below are based on the calibrated models from which we took the group-level effects of `trial` and build their posterior distributions. In this approach, as known, estimates are typically shrinked towards the mean, assuring robust modelling. Alternatively, we could have modelled a longitudinal

model using time (weeks or days) as temporal variable. However, in this context, we believe that order was more relevant than time and therefore we coded trial as a factor rather than a numeric variable.

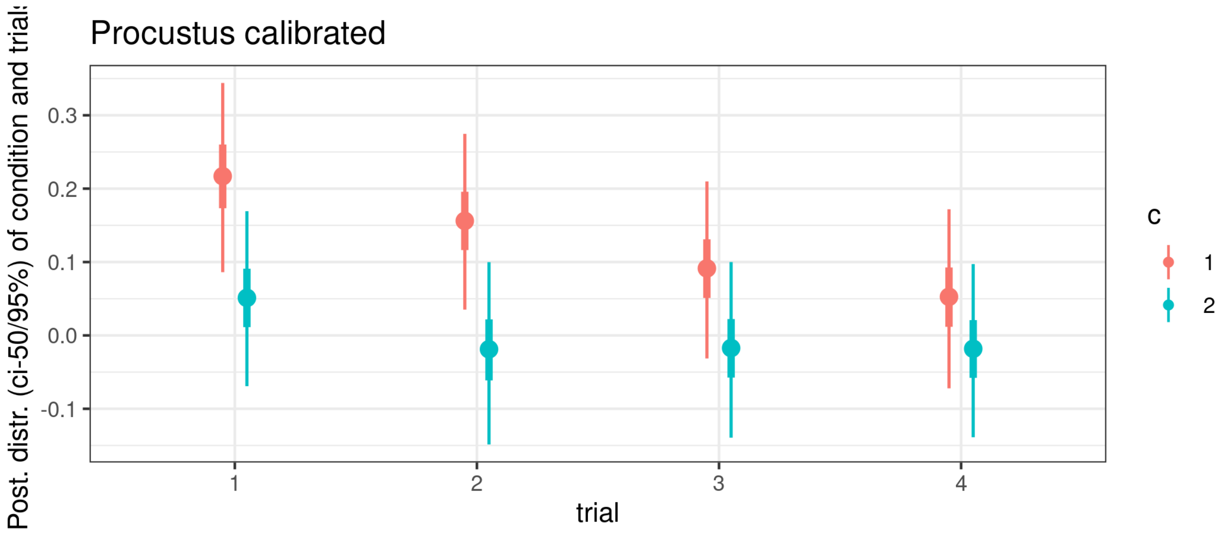

Figure 5: Posterior distributions of condition and trials – procustus1M

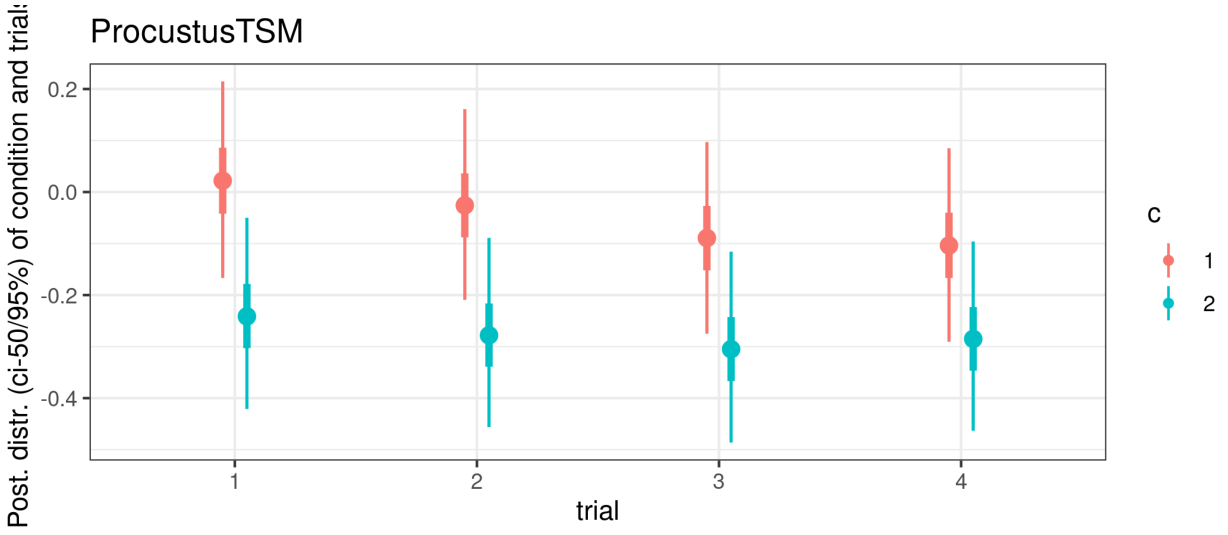

Figure 6: Posterior distributions of condition and trials – procustusTSM

Contrasts for TSM

Here we show the contrasts of the TSM modelling. Resuts are in line with the calibrated procustus.

```
load(file = "Results/hypothesis_test_procustusTSM.RData")

hypothesis_test_procustusTSM[[1]][,1]
```

```
##  [1] "(b_condition1)-(b_condition2) < 0"
```

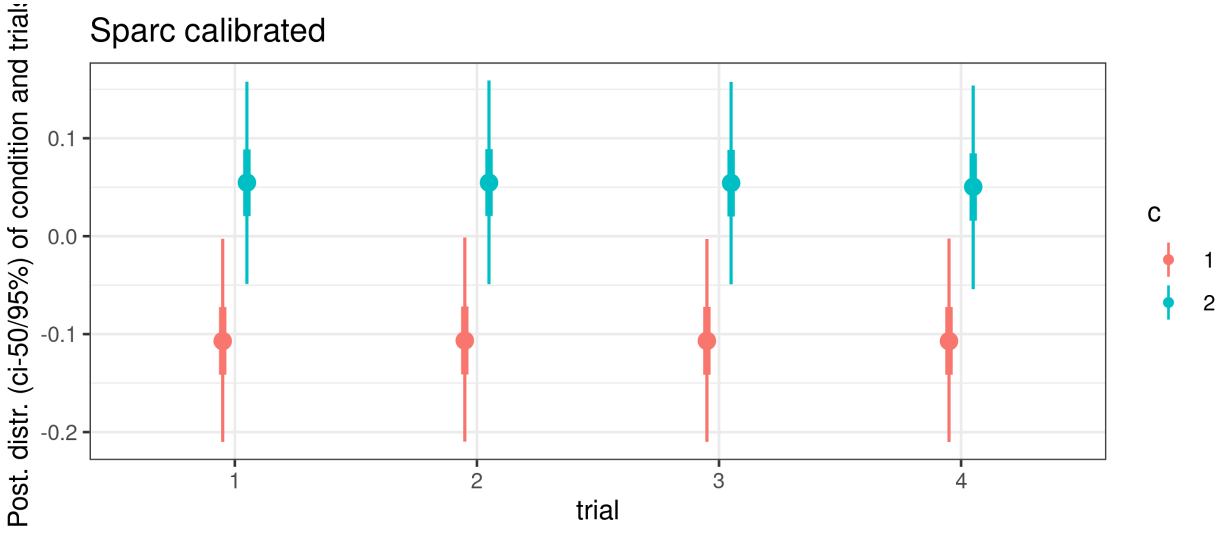

Figure 7: Posterior distributions of condition and trials – sparc1M

```
##  [2] "(r_condition:trial[1_1,Intercept])-(r_condition:trial[1_2,Intercept]) > 0"
##  [3] "(r_condition:trial[1_1,Intercept])-(r_condition:trial[1_3,Intercept]) > 0"
##  [4] "(r_condition:trial[1_1,Intercept])-(r_condition:trial[1_4,Intercept]) > 0"
##  [5] "(r_condition:trial[1_2,Intercept])-(r_condition:trial[1_3,Intercept]) > 0"
##  [6] "(r_condition:trial[1_2,Intercept])-(r_condition:trial[1_4,Intercept]) > 0"
##  [7] "(r_condition:trial[1_3,Intercept])-(r_condition:trial[1_4,Intercept]) > 0"
##  [8] "(r_condition:trial[2_1,Intercept])-(r_condition:trial[2_2,Intercept]) > 0"
##  [9] "(r_condition:trial[2_1,Intercept])-(r_condition:trial[2_3,Intercept]) > 0"
## [10] "(r_condition:trial[2_1,Intercept])-(r_condition:trial[2_4,Intercept]) > 0"
## [11] "(r_condition:trial[2_2,Intercept])-(r_condition:trial[2_3,Intercept]) > 0"
## [12] "(r_condition:trial[2_2,Intercept])-(r_condition:trial[2_4,Intercept]) > 0"
## [13] "(r_condition:trial[2_3,Intercept])-(r_condition:trial[2_4,Intercept]) > 0"
## [14] "(b_condition1+r_condition:trial[1_1,Intercept])-(b_condition2+r_condition:trial[2_1,Intercept]
## [15] "(b_condition1+r_condition:trial[1_2,Intercept])-(b_condition2+r_condition:trial[2_2,Intercept]
## [16] "(b_condition1+r_condition:trial[1_3,Intercept])-(b_condition2+r_condition:trial[2_3,Intercept]
## [17] "(b_condition1+r_condition:trial[1_4,Intercept])-(b_condition2+r_condition:trial[2_4,Intercept]
```

```
hypothesis_test_procustusTSM[[1]][,-1]
```

```
##     Label Estimate CI.Lower CI.Upper Post.Prob Star
## t1    c12     0.22     0.00     0.44      0.05
## t2  c1t12     0.05    -0.03     0.14      0.81
## t3  c1t13     0.11     0.00     0.22      0.96    *
## t4  c1t14     0.13     0.01     0.24      0.97    *
## t5  c1t23     0.06    -0.02     0.16      0.88
## t6  c1t24     0.08    -0.01     0.18      0.92
## t7  c1t34     0.01    -0.07     0.10      0.61
## t8  c2t12     0.04    -0.04     0.13      0.76
## t9  c2t13     0.06    -0.02     0.16      0.88
## t10 c2t14     0.04    -0.04     0.14      0.80
## t11 c2t23     0.03    -0.05     0.12      0.70
## t12 c2t24     0.01    -0.07     0.09      0.56
## t13 c2t34    -0.02    -0.11     0.06      0.35
```

```
## t14 c12t1     0.26     0.04     0.48      0.97    *
## t15 c12t2     0.25     0.03     0.46      0.97    *
## t16 c12t3     0.21     0.00     0.43      0.95
## t17 c12t4     0.18    -0.04     0.40      0.91
```

```
hypothesis_test_procustusTSM[[2]]
```

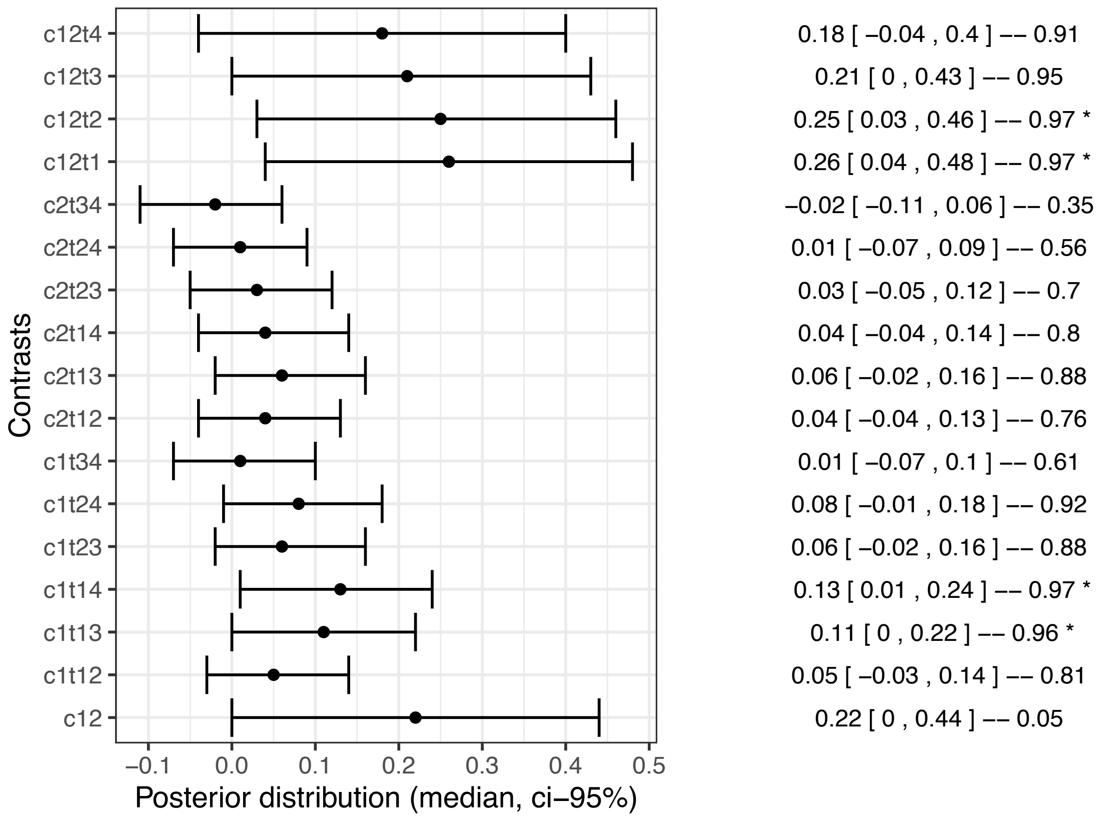

PART 2

Part 2 of this analysis is related to questionnaire models and hypothesis 3 and 4, following the workflow of Figure 1 (bottom part).

1. Comparison

We tested the models for the 4 questions and report here the log of the Bayes factor.

non-calibrated

```
## [1] "Bayes factor in favor of model_3 over model_4 (WPQ_OM):  3.23808397715852"
## [2] "Bayes factor in favor of model_3 over model_4 (WPQ_OM):  TRUE"

## [1] "Bayes factor in favor of model_3 over model_4 (MPQS_OM):  3.48749347270237"
## [2] "Bayes factor in favor of model_3 over model_4 (MPQS_OM):  TRUE"
```

```
## [1] "Bayes factor in favor of model_3 over model_4 (MPQP_0M):  3.35170469945722"
## [2] "Bayes factor in favor of model_3 over model_4 (MPQP_0M):  TRUE"
```

```
## [1] "Bayes factor in favor of model_3 over model_4 (Difficulty_0M):  2.79861803356677"
## [2] "Bayes factor in favor of model_3 over model_4 (Difficulty_0M):  TRUE"
```

calibrated

```
## [1] "Bayes factor in favor of model_3 over model_4 (WPQ_1M):  4.21250426730387"
## [2] "Bayes factor in favor of model_3 over model_4 (WPQ_1M):  TRUE"
```

```
## [1] "Bayes factor in favor of model_3 over model_4 (MPQS_1M):  2.8211870036081"
## [2] "Bayes factor in favor of model_3 over model_4 (MPQS_1M):  TRUE"
```

```
## [1] "Bayes factor in favor of model_3 over model_4 (MPQP_1M):  3.37800328385366"
## [2] "Bayes factor in favor of model_3 over model_4 (MPQP_1M):  TRUE"
```

```
## [1] "Bayes factor in favor of model_3 over model_4 (Difficulty_1M):  2.18813696147069"
## [2] "Bayes factor in favor of model_3 over model_4 (Difficulty_1M):  TRUE"
```

From this analysis it can be concluded that there is moderate evidence for model_3 for presence, and anecdotical evidence for model_3 for difficulty. Basically, it means that `procustus` does not contribute to an explanation of those responses.

2. Diagnostics

We show the diagnostics both for the 4 question models:

- the model_1 formula,
- the Bayes_R2 analysis,
- the model parameters,
- the posterior prediction check (pp_check) next to the plot of fixed parameters (i.e. `condition`)

Given the fact that `procustus` has no substantial influence we use model_1 (without `procustus`)

```
## [[1]]
## WPQ ~ 0 + condition + (1 | condition:participant + condition:trial)
##
## [[2]]
```

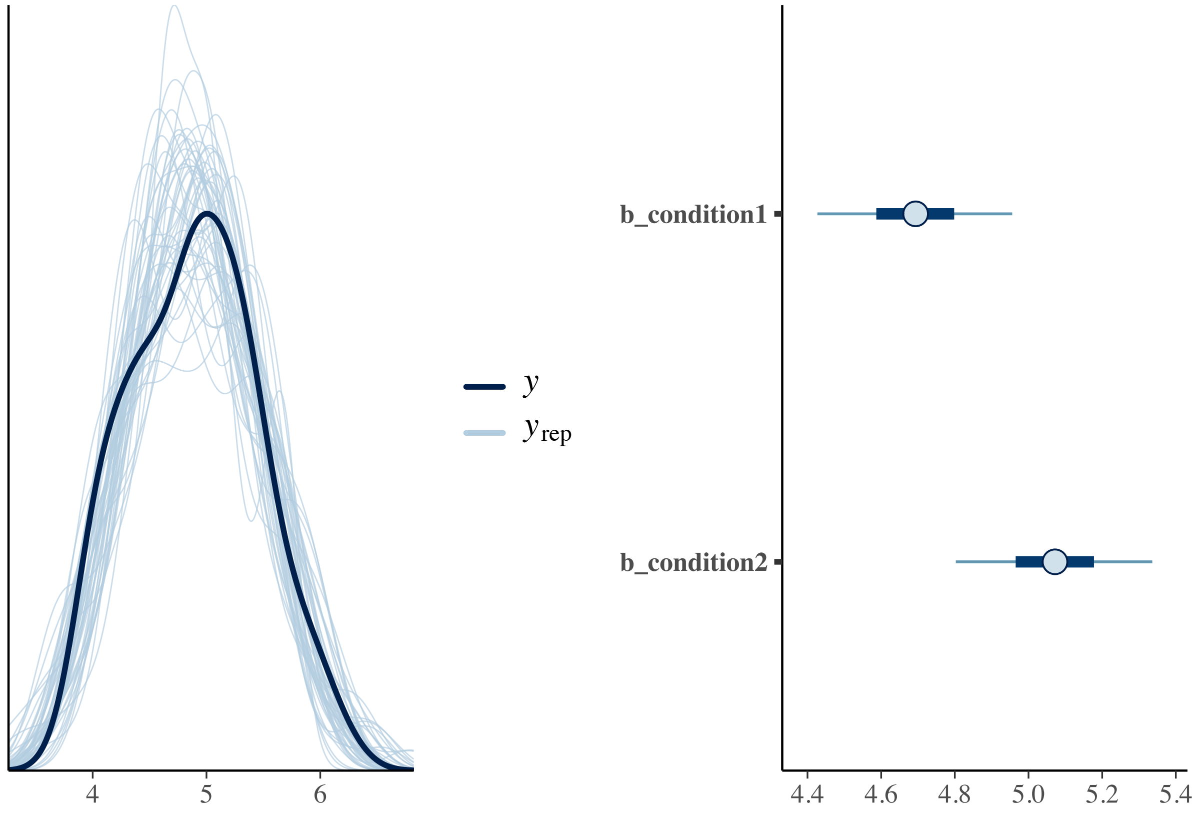


```
##
## [[3]]
##     R2   SD   CI CI_low CI_high CI_method   Component         Effectsize
## 1 0.73 0.04 0.95   0.65    0.79       HDI conditional Bayesian R-squared
## 2 0.12 0.11 0.95   0.00    0.32       HDI    marginal Bayesian R-squared
##
## [[4]]
##                             Parameter Effects   Component Mean   CI CI_low
## 1                        b_condition1   fixed conditional 4.69 0.95   4.37
## 2                        b_condition2   fixed conditional 5.07 0.95   4.75
## 3 sd_condition:participant__Intercept  random conditional 0.48 0.95   0.34
## 4       sd_condition:trial__Intercept  random conditional 0.08 0.95   0.00
## 5                               sigma   fixed       sigma 0.30 0.95   0.25
##   CI_high pd log_BF Rhat      ESS
## 1    5.01  1  56.78    1 27161.72
## 2    5.39  1  78.13    1 28582.79
## 3    0.69  1  20.77    1 30035.87
## 4    0.24  1  -3.27    1 47334.57
## 5    0.36  1  40.22    1 81251.62

## [[1]]
## MPQS ~ 0 + condition + (1 | condition:participant + condition:trial)
##
## [[2]]
```

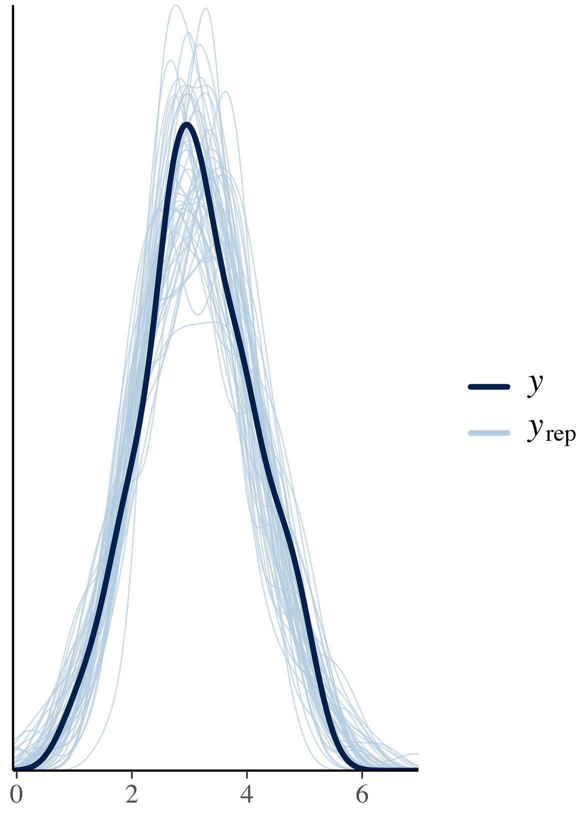

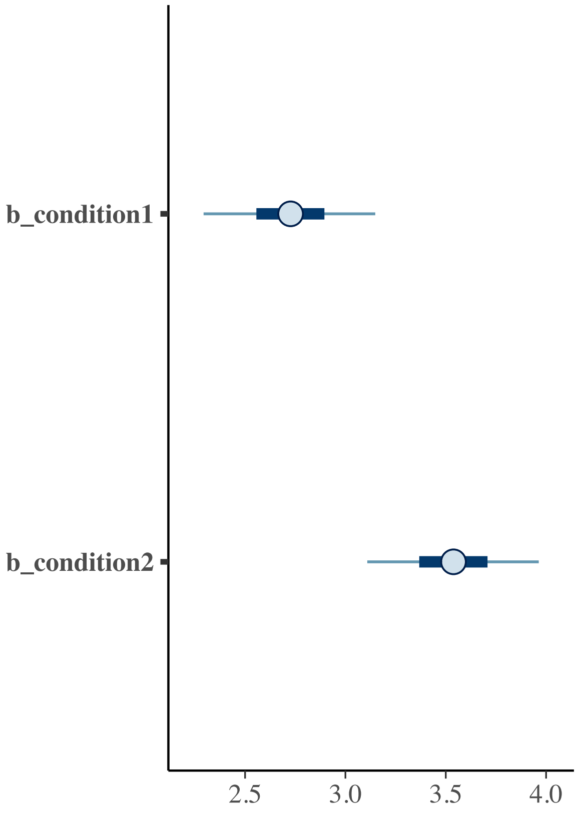


```
##
## [[3]]
##     R2   SD   CI CI_low CI_high CI_method   Component         Effectsize
## 1 0.66 0.05 0.95   0.56    0.74       HDI conditional Bayesian R-squared
## 2 0.18 0.12 0.95   0.00    0.37       HDI    marginal Bayesian R-squared
##
## [[4]]
##                             Parameter Effects   Component Mean   CI CI_low
## 1                        b_condition1   fixed conditional 2.72 0.95   2.20
## 2                        b_condition2   fixed conditional 3.54 0.95   3.02
## 3 sd_condition:participant__Intercept  random conditional 0.73 0.95   0.49
## 4       sd_condition:trial__Intercept  random conditional 0.17 0.95   0.01
## 5                               sigma   fixed       sigma 0.58 0.95   0.48
##   CI_high pd log_BF Rhat      ESS
## 1    3.24  1  17.38    1 31127.91
## 2    4.05  1  22.39    1 31307.50
## 3    1.06  1  17.46    1 35700.87
## 4    0.48  1  -2.57    1 45038.29
## 5    0.69  1  54.37    1 85891.55

## [[1]]
## MPQP ~ 0 + condition + (1 | condition:participant + condition:trial)
##
## [[2]]
```

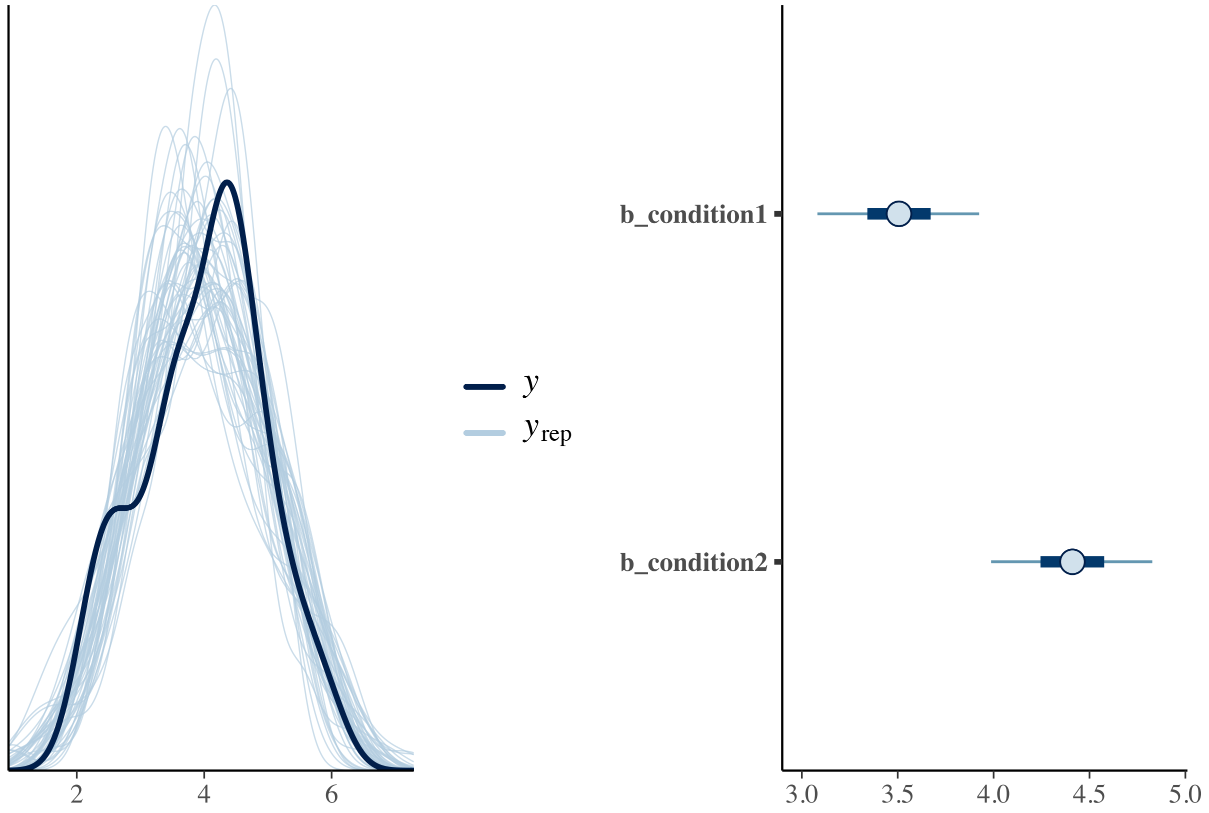


```
##
## [[3]]
##     R2   SD   CI CI_low CI_high CI_method   Component         Effectsize
## 1 0.65 0.05 0.95   0.53    0.73       HDI conditional Bayesian R-squared
## 2 0.22 0.13 0.95   0.00    0.41       HDI    marginal Bayesian R-squared
##
## [[4]]
##                               Parameter Effects   Component Mean   CI CI_low
## 1                          b_condition1   fixed conditional 3.51 0.95   2.99
## 2                          b_condition2   fixed conditional 4.41 0.95   3.89
## 3 sd_condition:participant__Intercept  random conditional 0.68 0.95   0.45
## 4       sd_condition:trial__Intercept  random conditional 0.20 0.95   0.01
## 5                                 sigma   fixed       sigma 0.59 0.95   0.50
##   CI_high pd log_BF Rhat      ESS
## 1    4.02  1  15.63    1 37493.93
## 2    4.91  1  23.69    1 37203.86
## 3    0.99  1  14.94    1 37492.70
## 4    0.56  1  -2.04    1 41823.47
## 5    0.72  1  45.60    1 81490.67
```

3. Contrasts

```
##   Label Estimate CI.Lower CI.Upper Post.Prob Star
## 1   c12    -0.38    -0.75     0.00      0.95    *
## 2   c12    -0.81    -1.42    -0.21      0.98    *
```

```
## 3   c12    -0.90    -1.50    -0.31      0.99    *
```

Data Analysis

Detailed workflow:

1. Alignment of motion capture (MoCap) data and audio data of avatar (section leader) and participant.
2. From the MoCap data, calculate the distance between the frog (the beginning of the bow) and the violin string (fid_1), and between the contact point between bow and string and the bridge (fid_2), both in avatar and participant.
3. Every instance diff(fid_1) changes sign, the bow movement changes direction relative to the string. A bow stroke is defined as the bow motion contained between 2 successive changes of sign of diff(fid_1).
4. Identify start and end time of each individual bow stroke of the avatar, defining a set of ROIs.
5. Retain only ROIs in which avatar bow strokes cover a distance along the strings equal or longer than 150 mm.
6. Retain only ROIs in which avatar bow strokes produce a sound with a loudness level equal or higher than 15% of the median of the loudness of the entire recording.
7. A set of ROIs is defined in the data, defined as the start and end times of those bow strokes that travel a certain distance and reach a certain loudness in the avatar.
8. These ROIs are used to construct a dataset of synchronized bow movements (both fid_1 and fid_2) of both avatar and participant.
9. The 2D Procrustes distance (PD) between the Mx2 column vector [fid_1,fid_2] of avatar and participant is calculated for each bow stroke [1] (cf. code below).
10. The SPARC index (SI) is calculated on fid_1 of avatar and participant as adapted for MATLAB from [2] (cf. code below). The difference of the SPARC index (dSI) is calculated as the difference $dSI = SI_{participant} – SI_{avatar}$.

PD MATLAB code:

```
function [cm] = PD(P,Q)

    % to be tested
    xP = P(:,1);
    yP = P(:,2);

    % reference
    xQ = Q(:,1);
    yQ = Q(:,2);

    % calculate 2D Procrusted distance
    sP = sqrt((sum((xP - nanmean(xP)).^2) / numel(xP)) + (sum((yP -
nanmean(yP)).^2) / numel(yP)));
    sQ = sqrt((sum((xQ - nanmean(xQ)).^2) / numel(xQ)) + (sum((yQ -
nanmean(yQ)).^2) / numel(yQ)));

    yP = (yP - nanmean(yP))/sP; yP = yP(:);
    yQ = (yQ - nanmean(yQ))/sQ; yQ = yQ(:);
    xP = (xP - nanmean(xP))/sP; xP = xP(:);
    xQ = (xQ - nanmean(xQ))/sQ; xQ = xQ(:);

    theta = atan2(sum(xQ.*yP - xP.*yQ), sum(xP.*xQ + yP.*yQ));
```

```
    xQ = cos(theta)*xQ - sin(theta)*yQ;
    yQ = sin(theta)*xQ + cos(theta)*yQ;

    cm = mean(sqrt((xP-xQ).^2 + (yP-yQ).^2));

end
```

SI MATLAB code:

```
function Output = Sparc(Input1, Input2, Input3, Input4, Input5)

    %     Calculates the smoothness of the given speed profile using the modified
    %     spectral arc length metric.
    %
    %     Parameters
    %     ----------
    %     Input1 : The array containing the movement speed profile.
    %     Input2 : The sampling frequency of the data.
    %     Input3 : Indicates the amount of zero padding to be done to the movement
    %              data for estimating the spectral arc length. [default = 4]
    %     Input4 : The max. cut off frequency for calculating the spectral arc
    %              length metric. [default = 10.]
    %     Input5 : The amplitude threshold to used for determing the cut off
    %              frequency upto which the spectral arc length is to be estimated.
    %              [default = 0.05]
    %
    %     Returns
    %     -------
    %     Output : The spectral arc length estimate of the given movement's
    %              smoothness.

    if nargin < 2
        Input2 = 120;
        Input3 = 4;
        Input4 = 10;
        Input5 = 0.05;
    end

    try

    % Number of zeros to be padded.
    nfft = round(2.^(ceil(log2(numel(Input1))) + Input3));

    % Frequency
    f = (0 : (Input2 / nfft) : Input2);

    % Normalized magnitude spectrum
    tmp = zeros(1,nfft);
    tmp(1:numel(Input1)) = Input1;
    Mf = abs(fft(tmp));
    Mf = Mf / max(Mf);

    % Indices to choose only the spectrum within the given cut off frequency Fc.
    % NOTE: This is a low pass filtering operation to get rid of high frequency
    % noise from affecting the next step (amplitude threshold based cut off for
    % arc length calculation).
    sel = find(f <= Input4);
    f_sel = f(sel);
    Mf_sel = Mf(sel);

    % Choose the amplitude threshold based cut off frequency.
    % Index of the last point on the magnitude spectrum that is greater than
    % or equal to the amplitude threshold.
    sel = find(Mf_sel >= Input5);
    sel = (sel(1):1:sel(end));
    f_sel = f_sel(sel);
```

```
    Mf_sel = Mf_sel(sel);

    % Calculate arc length
    Output = -sum( sqrt( (diff(f_sel) / (f_sel(end) - f_sel(1))).^2 +
(diff(Mf_sel)).^2));

    catch
        Output = nan;
    end
end
```

Questionnaires: Musical Background

VR experience 1

There are 60 questions in this survey.

Welkom!

Beste deelnemer,

U bent gevraagd mee te werken aan een onderzoek omtrent het gebruik van Virtual Reality (VR) technologie bij het spelen van muziek. Dit onderzoek wordt georganiseerd door de Universiteit Gent, faculteit Letteren en Wijsbegeerte, en staat onder leiding van Aleksandra Michalko, Adriaan Campo en prof. dr. Marc Leman. Met ons onderzoek willen we de ervaring van de musici tijdens het spelen in VR beter te beschrijven en begrijpen. Als u akkoord gaat om deel te nemen aan deze studie zal u gevraagd worden om relevante algemene informatie te verschaffen over uw demografische, educationele, medische en muzikale achtergrond. Aangezien we de gestandariseerde vragenlijsten gebruiken die in het Engels gevalideerd worden, worden hierop volgende vragen in het Engels gesteld.

Voor vragen of opmerkingen kan je terecht bij Aleksandra Michalko (aleksandra.michalko@ugent.be) of Adriaan Campo (adriaan.campo@ugent.be).

Door op "Ga door" te klikken, aanvaard je om deel te nemen aan deze studie aan bovenvermelde voorwaarden.

*

Please choose **only one** of the following:

◯ Ga door

◯ Ik wens niet deel te nemen

Please, fill in your participant's ID: *

Please write your answer here:

Demographics

What is your age?

*

Please write your answer here:

What gender do you identify as?

*

Please choose **only one** of the following:

- Female
- Male
- Prefer not to say
- Other

Do you have normal (or corrected to normal) vision?

*

Please choose **only one** of the following:

- ◯ Yes
- ◯ No
- ◯ I do not know

Make a comment on your choice here:

Do you have normal (or corrected to normal) hearing?

*

Please choose **only one** of the following:

- ◯ Yes
- ◯ No
- ◯ I do not know

Make a comment on your choice here:

Do you have any motor impariments?

*

Please choose **only one** of the following:

- Yes
- No
- I do not know

Make a comment on your choice here:

What is the highest degree or level of musi education you have completed?

*

Please choose **only one** of the following:

- Privéleraar
- Muziekacademie/ Muziekschool/ Privéschool
- Hoger onderwijs (vb. Conservatorium, musicologie…)
- Autodidact
- Other

What age did you start to play violin?

*

Please write your answer here:

Do you play other instruments besides violin? If so, which ones?

Please write your answer here:

Do you have absolute pitch? Absolute or perfect pitch is the ability to recognise and name an isolated musical tone without a reference tone, e.g. being able to say 'F#' if someone plays that note on the piano.

*

Please choose **only one** of the following:

- Yes
- No

MSI

I played/have been playing in the orchestra for __ years.

*

Please write your answer here:

I follow violin lessons for ___ hours per week.

*

Please write your answer here:

On average, I practice violin for _ hours per day.

*

Please choose the appropriate response for each item:

0	0.5	1	1.5	2	3-4	5 or more
○	○	○	○	○	○	○

I have had formal training in music theory for _ years.

*

Please write your answer here:

I spend a lot of my free time doing music-related activities.

*

Please choose the appropriate response for each item:

Completely disagree	Strongly disagree	Disagree	Neither agree nor disagree	Agree	Strongly agree	Completely agree
◯	◯	◯	◯	◯	◯	◯

I enjoy writing about music, for example on blogs and forums.

*

Please choose the appropriate response for each item:

Completely disagree	Strongly disagree	Disagree	Neither agree nor disagree	Agree	Strongly agree	Completely agree
◯	◯	◯	◯	◯	◯	◯

I often read or search the internet for things related to music.

*

Please choose the appropriate response for each item:

Completely disagree	Strongly disagree	Disagree	Neither agree nor disagree	Agree	Strongly agree	Completely agree
○	○	○	○	○	○	○

I'm intrigued by musical styles I'm not familiar with and want to find out more.

*

Please choose the appropriate response for each item:

Completely disagree	Strongly disagree	Disagree	Neither agree nor disagree	Agree	Strongly agree	Completely agree
○	○	○	○	○	○	○

Music is like of an addiction to me - I couldn't live without it.

*

Please choose the appropriate response for each item:

Completely disagree	Strongly disagree	Disagree	Neither agree nor disagree	Agree	Strongly agree	Completely agree
○	○	○	○	○	○	○

I listen attentively to music for __ per day.

*

Please choose the appropriate response for each item:

0-15 min	15-30 min	30-60 min	60-90 min	2 hrs	2-3 hrs	4 hrs or more
○	○	○	○	○	○	○

I keep track of new music that I come across (e.g. new artists or recordings).

*

Please choose the appropriate response for each item:

Completely disagree	Strongly disagree	Disagree	Neither agree nor disagree	Agree	Strongly agree	Completely agree
○	○	○	○	○	○	○

I find it difficult to spot mistakes in a performance of a song even if I know the tune.

*

Please choose the appropriate response for each item:

Completely disagree	Strongly disagree	Disagree	Neither agree nor disagree	Agree	Strongly agree	Completely agree
○	○	○	○	○	○	○

I usually know when I'm hearing a song for the first time.

*

Please choose the appropriate response for each item:

Completely disagree	Strongly disagree	Disagree	Neither agree nor disagree	Agree	Strongly agree	Completely agree
◯	◯	◯	◯	◯	◯	◯

I have trouble recognizing a familiar song when played in a different way or by a different performer.

*

Please choose the appropriate response for each item:

Completely disagree	Strongly disagree	Disagree	Neither agree nor disagree	Agree	Strongly agree	Completely agree
◯	◯	◯	◯	◯	◯	◯

I can compare and discuss differences between two performances or versions of the same piece of music.

*

Please choose the appropriate response for each item:

Completely disagree	Strongly disagree	Disagree	Neither agree nor disagree	Agree	Strongly agree	Completely agree
○	○	○	○	○	○	○

I can tell when people sing or play out of time with the beat.

*

Please choose the appropriate response for each item:

Completely disagree	Strongly disagree	Disagree	Neither agree nor disagree	Agree	Strongly agree	Completely agree
○	○	○	○	○	○	○

I can tell when people sing or play out of tune.

*

Please choose the appropriate response for each item:

Completely disagree	Strongly disagree	Disagree	Neither agree nor disagree	Agree	Strongly agree	Completely agree
◯	◯	◯	◯	◯	◯	◯

When I sing, I have no idea whether I'm in tune or not.

*

Please choose the appropriate response for each item:

Completely disagree	Strongly disagree	Disagree	Neither agree nor disagree	Agree	Strongly agree	Completely agree
◯	◯	◯	◯	◯	◯	◯

I engaged in regular, daily practice of violin for_ years.

*

Please choose the appropriate response for each item:

0	1	2	3	4-5	6-9	10 or more
◯	◯	◯	◯	◯	◯	◯

At the peak of my interest, I practised violin for _ hours per day.

*

Please choose the appropriate response for each item:

0	0.5	1	1.5	2	3-4	5 or more
○	○	○	○	○	○	○

I have never been complimented for my talents as a musical performer.

*

Please choose the appropriate response for each item:

Completely disagree	Strongly disagree	Disagree	Neither agree nor disagree	Agree	Strongly agree	Completely agree
○	○	○	○	○	○	○

I am able to identify what is special about a given musical piece.

*

Please choose the appropriate response for each item:

Completely disagree	Strongly disagree	Disagree	Neither agree nor disagree	Agree	Strongly agree	Completely agree
○	○	○	○	○	○	○

I have had _ years of formal training on violin during my lifetime.

*

Please choose the appropriate response for each item:

0	0.5	1	2	3-5	6-9	10 or more
◯	◯	◯	◯	◯	◯	◯

I would not consider myself a musician.

*

Please choose the appropriate response for each item:

Completely disagree	Strongly disagree	Disagree	Neither agree nor disagree	Agree	Strongly agree	Completely agree
◯	◯	◯	◯	◯	◯	◯

I can sing or play music from memory.

*

Please choose the appropriate response for each item:

Completely disagree	Strongly disagree	Disagree	Neither agree nor disagree	Agree	Strongly agree	Completely agree
◯	◯	◯	◯	◯	◯	◯

VR

Virtual reality (VR) refers to a computer-generated simulation in which a person can interact with an artificial three-dimensional environment using electronic devices, such as special goggles with a screen or gloves fitted with sensors. VR technology gives users a truly immersive, first-person perspective in virtually created situations. Most importantly, users can interact with these virtual environments. VR is used in healthcare to practice procedures and surgical operation, in sport to improve athletes' cognitive abilities and education for teaching and learning situations.

Have you ever used VR?

*

Please choose **only one** of the following:

- ◯ Yes
- ◯ No

How often do you use VR technology?

*

Please write your answer here:

In which context do or did you use VR technology?

*

Please write your answer here:

Have you ever used VR in the context of music performance or music education? If yes, please describe the context.

*

Please choose **only one** of the following:

- ◯ Yes
- ◯ No

Make a comment on your choice here:

When you use VR, how long does your VR session usually last?

*

Please choose **only one** of the following:

- Less than 20 min
- 20 - 40 min
- 40 - 60 min
- More than 60 min

Could you describe the sensations you experience while being in a VR environment?

*

Please write your answer here:

ITQ

Do you easily become deeply involved in movies or tv dramas?

*

Please choose the appropriate response for each item:

1 - Never	2	3	4 - Occasionally	5	6	7 - Often
○	○	○	○	○	○	○

Do you ever become so involved in a television program or book that people have problems getting your attention?

*

Please choose the appropriate response for each item:

1 - Never	2	3	4 - Occasionally	5	6	7 - Often
○	○	○	○	○	○	○

How mentally alert do you feel at the present time?

*

Please choose the appropriate response for each item:

1 - Not alert	2	3	4 - Moderately	5	6	7 - Fully alert
○	○	○	○	○	○	○

Do you ever become so involved in a movie that you are not aware of things happening around you?

*

Please choose the appropriate response for each item:

1 - Never	2	3	4 - Occasionally	5	6	7 - Often
○	○	○	○	○	○	○

How frequently do you find yourself closely identifying with the characters in a story line?

*

Please choose the appropriate response for each item:

1 - Never	2	3	4 - Occasionally	5	6	7 - Often
○	○	○	○	○	○	○

Do you ever become so involved in a video game that it is as if you are inside the game rather than moving a joystick and watching the screen?

*

Please choose the appropriate response for each item:

1 - Never	2	3	4 - Occasionally	5	6	7 - Often
○	○	○	○	○	○	○

How physically fit do you feel today?

*

Please choose the appropriate response for each item:

1- Not fit	2	3	4 - Moderately fit	5	6	7 - Extremely fit
◯	◯	◯	◯	◯	◯	◯

How good are you at blocking out external distractions when you are involved in something?

*

Please choose the appropriate response for each item:

1 - Not very good	2	3	4 - Somewhat good	5	6	7 - Very good
◯	◯	◯	◯	◯	◯	◯

When watching sports, do you ever become so involved in the game that you react as if you were one of the players?

*

Please choose the appropriate response for each item:

1 - Never	2	3	4 - Occasionally	5	6	7 - Often
◯	◯	◯	◯	◯	◯	◯

Do you ever become so involved in a daydream that you are not aware of things happening around you?

*

Please choose the appropriate response for each item:

1 - Never	2	3	4 - Occasionally	5	6	7 - Often
○	○	○	○	○	○	○

Do you ever have dreams that are so real that you feel disoriented when you awake?

*

Please choose the appropriate response for each item:

1 - Never	2	3	4 - Occasionally	5	6	7 - Often
○	○	○	○	○	○	○

When playing sports, do you become so involved in the game that you lose track of time?

*

Please choose the appropriate response for each item:

1 - Never	2	3	4 - Occasionally	5	6	7 - Often
○	○	○	○	○	○	○

How well do you concentrate on enjoyable activities?

*

Please choose the appropriate response for each item:

1 - Not at all	2	3	4 - Moderately well	5	6	7 - Very well
○	○	○	○	○	○	○

How often do you play arcade or video games? (OFTEN should be taken to mean every day or every two days, on average.)

*

Please choose the appropriate response for each item:

1 - Never	2	3	4 - Occasionally	5	6	7 - Often
○	○	○	○	○	○	○

Have you ever gotten excited during a chase or fight scene on TV or in the movies?

*

Please choose the appropriate response for each item:

1 - Never	2	3	4 - Occasionally	5	6	7 - Often
○	○	○	○	○	○	○

Have you ever gotten scared by something happening on a TV show or in a movie?

*

Please choose the appropriate response for each item:

1 - Never	2	3	4 - Occasionally	5	6	7 - Often
◯	◯	◯	◯	◯	◯	◯

Have you ever remained apprehensive or fearful long after watching a scary movie?

*

Please choose the appropriate response for each item:

1 - Never	2	3	4 - Occasionally	5	6	7 - Often
◯	◯	◯	◯	◯	◯	◯

Do you ever become so involved in doing something that you lose all track of time?

*

Please choose the appropriate response for each item:

1 - Never	2	3	4 - Occasionally	5	6	7 - Often
◯	◯	◯	◯	◯	◯	◯

Bedankt voor uw medewerking.

We zien elkaar binnekort in ons lab!

De Krook, Miriam Makebaplein 1, 9000 Ghent, Belgium

Submit your survey.

Thank you for completing this survey.

Questionnaires: 2D condition

Mixed Reality Beamer

Welcome to the survey!

There are 61 questions in this survey.

Please fill in below your participant ID number and session number

*

Session *

Choose one of the following answers

Please choose **only one** of the following:

- 1
- 2
- 3
- 4

SOMA

Please indicate how much you agree or disagree with each statement by moving the slide handle.

I was in full control of the music. *

Each answer must be between 0 and 100

Please write your answer(s) here:

|Strongly disagree|Strongly agree

I feel like the music was controlled by someone else. *

Each answer must be between 0 and 100

Please write your answer(s) here:

|Strongly disagree|Strongly agree

The way I made the music was not in line with my intentions. *

Each answer must be between 0 and 100

Please write your answer(s) here:

|Strongly disagree|Strongly agree

I felt like the author of my musical actions. *

Each answer must be between 0 and 100

Please write your answer(s) here:

|Strongly disagree|Strongly agree

I did not feel like the music I played logically followed from my interactions with the instrument/musical interface. *

Each answer must be between 0 and 100

Please write your answer(s) here:

|Strongly disagree|Strongly agree

The music was automatic – I could not control it through my actions. *

Each answer must be between 0 and 100

Please write your answer(s) here:

|Strongly disagree|Strongly agree

I was puzzled by which musical actions corresponded to which musical outcomes. *

Each answer must be between 0 and 100

Please write your answer(s) here:

|Strongly disagree|Strongly agree

The music I played was subject to my free will. *

Each answer must be between 0 and 100

Please write your answer(s) here:

|Strongly disagree|Strongly agree

The decision of whether and when to play musical notes/patterns/sounds next in the music is entirely my own. *

Each answer must be between 0 and 100

Please write your answer(s) here:

|Strongly disagree|Strongly agree

Nothing I did in the music was actually voluntary. *

❗ Each answer must be between 0 and 100

Please write your answer(s) here:

|Strongly disagree|Strongly agree

I felt like a remote-controlled robot while playing the music. *

❗ Each answer must be between 0 and 100

Please write your answer(s) here:

|Strongly disagree|Strongly agree

My musical expressions/actions were planned by me from the very beginning to the very end. *

❗ Each answer must be between 0 and 100

Please write your answer(s) here:

|Strongly disagree|Strongly agree

I am completely responsible for any music that results from my actions. *

❗ Each answer must be between 0 and 100

Please write your answer(s) here:

|Strongly disagree|Strongly agree

MPS

The mixed environment seemed real to me.

*

Please choose the appropriate response for each item:

Completely disagree						Strongly agree
○	○	○	○	○	○	○

I had a sense of acting in the mixed environment, rather than operating something from outside.

*

Please choose the appropriate response for each item:

Completely disagree						Strongly agree
○	○	○	○	○	○	○

My experience in the mixed environment seemed consistent with my experiences in the real world.

*

Please choose the appropriate response for each item:

Completely disagree						Strongly agree
○	○	○	○	○	○	○

While I was in the mixed environment, I had a sense of “being there”.

*

Please choose the appropriate response for each item:

Completely disagree						Strongly agree
○	○	○	○	○	○	○

I was completely captivated by the virtual world.

*

Please choose the appropriate response for each item:

Completely disagree						Strongly agree
○	○	○	○	○	○	○

I felt like I was in the presence of another person in the mixed environment.

*

Please choose the appropriate response for each item:

Completely disagree						Strongly agree
○	○	○	○	○	○	○

I felt that the people in the mixed environment were aware of my presence.

*

Please choose the appropriate response for each item:

Completely disagree						Strongly agree
○	○	○	○	○	○	○

The people in the mixed environment appeared to be sentient (conscious and alive) to me.

*

Please choose the appropriate response for each item:

Completely disagree						Strongly agree
○	○	○	○	○	○	○

During the simulation there were times where the computer interface seemed to disappear, and I felt like I was working directly with another person.

*

Please choose the appropriate response for each item:

Completely disagree						Strongly agree
○	○	○	○	○	○	○

I had a sense that I was interacting with other people in the mixed environment, rather than a computer simulation.

*

Please choose the appropriate response for each item:

Completely disagree						Strongly agree
○	○	○	○	○	○	○

PQ VR

Vragenlijst voor VR applicatie.

How much were you able to control events? *

Please choose the appropriate response for each item:

1- Not at all	2	3	4 - Somewhat	5	6	7 - Completely
○	○	○	○	○	○	○

How responsive was the environment to actions that you initiated (or performed)? *

Please choose the appropriate response for each item:

1- Not responsive	2	3	4 - Moderately responsive	5	6	7 - Completely responsive
○	○	○	○	○	○	○

How natural did your interactions with the environment seem? *

Please choose the appropriate response for each item:

1- Extremely artificial	2	3	4 - Borderline	5	6	7 - Completely natural
○	○	○	○	○	○	○

How much did the visual aspects of the environment involve you? *

Please choose the appropriate response for each item:

1- Not at all	2	3	4 - Somewhat	5	6	7 - Completely
○	○	○	○	○	○	○

How natural was the mechanism which controlled movement through the environment? *

Please choose the appropriate response for each item:

1- Extremely artificial	2	3	4 - Borderline	5	6	7 - Completely natural
○	○	○	○	○	○	○

How compelling was your sense of objects moving through space? *

Please choose the appropriate response for each item:

1- Not at all	2	3	4 - Moderately compelling	5	6	7 - Very compelling
○	○	○	○	○	○	○

Were you able to anticipate what would happen next in response to the actions that you performed? *

Please choose the appropriate response for each item:

1- Not at all	2	3	4 - Somewhat	5	6	7 - Completely
○	○	○	○	○	○	○

How completely were you able to actively survey or search the environment using vision? *

Please choose the appropriate response for each item:

1- Not at all	2	3	4 - Somewhat	5	6	7 - Completely
○	○	○	○	○	○	○

How compelling was your sense of moving around inside the mixed environment? *

Please choose the appropriate response for each item:

1- Not at all	2	3	4 - Moderately compelling	5	6	7 - Very compelling
○	○	○	○	○	○	○

How closely were you able to examine objects? *

Please choose the appropriate response for each item:

1- Not at all	2	3	4 - Pretty closely	5	6	7 - Very closely
○	○	○	○	○	○	○

How well could you examine objects from multiple viewpoints? *

Please choose the appropriate response for each item:

1- Not at all	2	3	4 - Somewhat	5	6	7 - Extensively
○	○	○	○	○	○	○

How much delay did you experience between your actions and expected outcomes? *

Please choose the appropriate response for each item:

1- No delays	2	3	4 - Moderate delays	5	6	7 - Long delays
○	○	○	○	○	○	○

How quickly did you adjust to the mixed environment experience? *

Please choose the appropriate response for each item:

1- Not at all	2	3	4 - Slowly	5	6	7 - Less than one minute
○	○	○	○	○	○	○

How proficient in moving and interacting with the mixed environment did you feel at the end of the experience? *

Please choose the appropriate response for each item:

1- Not proficient	2	3	4 - Reasonably proficient	5	6	7 - Very proficient
○	○	○	○	○	○	○

How much did the visual display quality interfere or distract you from performing assigned tasks or required activities? *

Please choose the appropriate response for each item:

1- Not at all	2	3	4 - Interfered somewhat	5	6	7 - Prevented task performance
○	○	○	○	○	○	○

How much did the control devices interfere with the performance of assigned tasks or with other activities? *

Please choose the appropriate response for each item:

1- Not at all	2	3	4 - Interfered somewhat	5	6	7 - Interfered greatly
○	○	○	○	○	○	○

How well could you concentrate on the assigned tasks or required activities rather than on the mechanisms used to perform those tasks or activities? *

Please choose the appropriate response for each item:

1- Not at all	2	3	4 - Somewhat	5	6	7 - Completely
○	○	○	○	○	○	○

How much did the auditory aspects of the environment involve you? *

Please choose the appropriate response for each item:

1- Not at all	2	3	4 - Somewhat	5	6	7 - Completely
◯	◯	◯	◯	◯	◯	◯

How well could you identify sounds? *

Please choose the appropriate response for each item:

1- Not at all	2	3	4 - Somewhat	5	6	7 - Completely
◯	◯	◯	◯	◯	◯	◯

How well could you localize sounds? *

Please choose the appropriate response for each item:

1- Not at all	2	3	4 - Somewhat	5	6	7 - Completely
◯	◯	◯	◯	◯	◯	◯

How completely were your senses engaged in this experience?

*

Please choose the appropriate response for each item:

1- Not engaged	2	3	4 - Mildly engaged	5	6	7 - Completely engaged
○	○	○	○	○	○	○

Were there moments during the mixed environment experience when you felt completely focused on the task or environment?

*

Please choose the appropriate response for each item:

1- Not at all	2	3	4 - Sometimes	5	6	7 - All the time
○	○	○	○	○	○	○

How easily did you adjust to the control devices used to interact with the mixed environment?

*

Please choose the appropriate response for each item:

1- Not at all	2	3	4 - Somewhat difficult	5	6	7 - Very easily
○	○	○	○	○	○	○

Was the information provided through different senses in the mixed environment (e.g., vision, hearing, touch) consistent?

*

Please choose the appropriate response for each item:

1- Not consistent	2	3	4 - Moderately consistent	5	6	7 - Very consistent
○	○	○	○	○	○	○

To what extent did you feel completely surrounded by and enveloped by the mixed environment?

*

Please choose the appropriate response for each item:

1- Not at all	2	3	4 - Somewhat	5	6	7 - Completely
○	○	○	○	○	○	○

As you moved through the mixed environment and interacted with it, did you feel like you were inside the mixed environment, affecting or being affected by objects and events in that environment?

*

Please choose the appropriate response for each item:

1- Not at all	2	3	4 - Moderately affected	5	6	7 - Very affected
○	○	○	○	○	○	○

How much did your experience in the mixed environment seem like you were in a real place, able to directly sense and interact with the environment?

*

Please choose the appropriate response for each item:

1- Not at all	2	3	4 - Somewhat	5	6	7 - Completely
○	○	○	○	○	○	○

How physically fit do you feel at the present time? *

Please choose the appropriate response for each item:

1- Not fit	2	3	4 - Moderately fit	5	6	7 - Extremely fit
◯	◯	◯	◯	◯	◯	◯

How mentally alert do you feel at the present time? *

Please choose the appropriate response for each item:

1- Not alert	2	3	4 - Moderately	5	6	7 - Fully alert
◯	◯	◯	◯	◯	◯	◯

OQ

If it is more convenient for you, answer in Dutch.

How effective was the training with the screen?
*

Please choose the appropriate response for each item:

1- Not at all effective	2	3	4	5	6	7 - Very effective
0 ◯	◯	◯	◯	◯	◯	◯

Please, argument your answer to the previous question. *

Please write your answer here:

How similar was it to the experience of practising at home with video? *

Please choose the appropriate response for each item:

	1- Not at all similar	2	3	4	5	6	7 - Extremely similar
3D	○	○	○	○	○	○	○

Please, argument your answer to the previous question. *

Please write your answer here:

Did your playing improve in comparison with the last session? Please, argument your answer.

Only answer this question if the following conditions are met:
Answer was NOT '1' at question '2 [PN002]' (Session)

Please write your answer here:

Are there any functions and/or features you would like to add to the VR application that would improve your training/playing more? Please give concrete examples.

Only answer this question if the following conditions are met:
Answer was '4' at question '2 [PN002]' (Session)

Please write your answer here:

In which view mode was it best for you to practice? Please, explain. *

Only answer this question if the following conditions are met:
Answer was '4' at question '2 [PN002]' (Session)

Please choose **only one** of the following:

- ◯ Condition with 3D avatar
- ◯ Condition with the beamer

Make a comment on your choice here:

Thank you for your participation!

Aleksandra Michalko & Adriaan Campo

If you have more questions about the experiment, do not hesitate to contact us!

aleksandra.michalko@ugent.be adriaan.campo@ugent.be

Submit your survey.
Thank you for completing this survey.

Questionnaires: 3D condition

Mixed Reality 3D

Welcome to the survey!

There are 67 questions in this survey.

Please fill in below your participant ID number and session number

*

Session

*

Please choose **only one** of the following:

- ◯ 1
- ◯ 2
- ◯ 3
- ◯ 4

SOMA

Please indicate how much you agree or disagree with each statement by moving the slide handle.

I was in full control of the music. *

Please write your answer(s) here:

|Strongly disagree|Strongly agree

I feel like the music was controlled by someone else.

*

Please write your answer(s) here:

|Strongly disagree|Strongly agree

The way I made the music was not in line with my intentions.

*

Please write your answer(s) here:

|Strongly disagree|Strongly agree

I felt like the author of my musical actions.

*

Please write your answer(s) here:

|Strongly disagree|Strongly agree

I did not feel like the music I played logically followed from my interactions with the instrument/musical interface.

*

Please write your answer(s) here:

|Strongly disagree|Strongly agree

The music was automatic – I could not control it through my actions.

*

Please write your answer(s) here:

|Strongly disagree|Strongly agree

I was puzzled by which musical actions corresponded to which musical outcomes.

*

Please write your answer(s) here:

|Strongly disagree|Strongly agree

The music I played was subject to my free will.

*

Please write your answer(s) here:

|Strongly disagree|Strongly agree

The decision of whether and when to play musical notes/patterns/sounds next in the music is entirely my own.

*

Please write your answer(s) here:

|Strongly disagree|Strongly agree

Nothing I did in the music was actually voluntary.

*

Please write your answer(s) here:

|Strongly disagree|Strongly agree

I felt like a remote-controlled robot while playing the music.

*

Please write your answer(s) here:

|Strongly disagree|Strongly agree

My musical expressions/actions were planned by me from the very beginning to the very end.

*

Please write your answer(s) here:

|Strongly disagree|Strongly agree

I am completely responsible for any music that results from my actions.

*

Please write your answer(s) here:

|Strongly disagree|Strongly agree

MPS

The mixed environment seemed real to me.

*

Please choose the appropriate response for each item:

Completely disagree						Strongly agree
○	○	○	○	○	○	○

I had a sense of acting in the mixed environment, rather than operating something from outside.

*

Please choose the appropriate response for each item:

**Completely disagree**						**Strongly agree**
○	○	○	○	○	○	○

My experience in the mixed environment seemed consistent with my experiences in the real world.

*

Please choose the appropriate response for each item:

**Completely disagree**						**Strongly agree**
○	○	○	○	○	○	○

While I was in the mixed environment, I had a sense of “being there”.

*

Please choose the appropriate response for each item:

**Completely disagree**						**Strongly agree**
○	○	○	○	○	○	○

I was completely captivated by the virtual world.

*

Please choose the appropriate response for each item:

Completely disagree						Strongly agree
○	○	○	○	○	○	○

I felt like I was in the presence of another person in the mixed environment.

*

Please choose the appropriate response for each item:

Completely disagree						Strongly agree
○	○	○	○	○	○	○

I felt that the people in the mixed environment were aware of my presence.

*

Please choose the appropriate response for each item:

Completely disagree						Strongly agree
○	○	○	○	○	○	○

The people in the mixed environment appeared to be sentient (conscious and alive) to me.

*

Please choose the appropriate response for each item:

Completely disagree						Strongly agree
○	○	○	○	○	○	○

During the simulation there were times where the computer interface seemed to disappear, and I felt like I was working directly with another person.

*

Please choose the appropriate response for each item:

Completely disagree						Strongly agree
○	○	○	○	○	○	○

I had a sense that I was interacting with other people in the mixed environment, rather than a computer simulation.

*

Please choose the appropriate response for each item:

Completely disagree						Strongly agree
○	○	○	○	○	○	○

PQ VR

Vragenlijst voor VR applicatie.

How much were you able to control events?

*

Please choose the appropriate response for each item:

1- Not at all	2	3	4 - Somewhat	5	6	7 - Completely
○	○	○	○	○	○	○

How responsive was the environment to actions that you initiated (or performed)?

*

Please choose the appropriate response for each item:

1- Not responsive	2	3	4 - Moderately responsive	5	6	7 - Completely responsive
○	○	○	○	○	○	○

How natural did your interactions with the environment seem?

*

Please choose the appropriate response for each item:

1- Extremely artificial	2	3	4 - Borderline	5	6	7 - Completely natural
○	○	○	○	○	○	○

How much did the visual aspects of the environment involve you?

*

Please choose the appropriate response for each item:

1- Not at all	2	3	4 - Somewhat	5	6	7 - Completely
◯	◯	◯	◯	◯	◯	◯

How natural was the mechanism which controlled movement through the environment?

*

Please choose the appropriate response for each item:

1- Extremely artificial	2	3	4 - Borderline	5	6	7 - Completely natural
◯	◯	◯	◯	◯	◯	◯

How compelling was your sense of objects moving through space?

*

Please choose the appropriate response for each item:

1- Not at all	2	3	4 - Moderately compelling	5	6	7 - Very compelling
◯	◯	◯	◯	◯	◯	◯

Were you able to anticipate what would happen next in response to the actions that you performed?

*

Please choose the appropriate response for each item:

1- Not at all	2	3	4 - Somewhat	5	6	7 - Completely
○	○	○	○	○	○	○

How completely were you able to actively survey or search the environment using vision?

*

Please choose the appropriate response for each item:

1- Not at all	2	3	4 - Somewhat	5	6	7 - Completely
○	○	○	○	○	○	○

How compelling was your sense of moving around inside the mixed environment?

*

Please choose the appropriate response for each item:

1- Not at all	2	3	4 - Moderately compelling	5	6	7 - Very compelling
○	○	○	○	○	○	○

How closely were you able to examine objects?

*

Please choose the appropriate response for each item:

1- Not at all	2	3	4 - Pretty closely	5	6	7 - Very closely
◯	◯	◯	◯	◯	◯	◯

How well could you examine objects from multiple viewpoints?

*

Please choose the appropriate response for each item:

1- Not at all	2	3	4 - Somewhat	5	6	7 - Extensively
◯	◯	◯	◯	◯	◯	◯

How much delay did you experience between your actions and expected outcomes?

*

Please choose the appropriate response for each item:

1- No delays	2	3	4 - Moderate delays	5	6	7 - Long delays
◯	◯	◯	◯	◯	◯	◯

How quickly did you adjust to the mixed environment experience?

*

Please choose the appropriate response for each item:

1- Not at all	2	3	4 - Slowly	5	6	7 - Less than one minute
◯	◯	◯	◯	◯	◯	◯

How proficient in moving and interacting with the mixed environment did you feel at the end of the experience?

*

Please choose the appropriate response for each item:

1- Not proficient	2	3	4 - Reasonably proficient	5	6	7 - Very proficient
◯	◯	◯	◯	◯	◯	◯

How much did the visual display quality interfere or distract you from performing assigned tasks or required activities?

*

Please choose the appropriate response for each item:

1- Not at all	2	3	4 - Interfered somewhat	5	6	7 - Prevented task performance
○	○	○	○	○	○	○

How much did the control devices interfere with the performance of assigned tasks or with other activities?

*

Please choose the appropriate response for each item:

1- Not at all	2	3	4 - Interfered somewhat	5	6	7 - Interfered greatly
○	○	○	○	○	○	○

How well could you concentrate on the assigned tasks or required activities rather than on the mechanisms used to perform those tasks or activities?

*

Please choose the appropriate response for each item:

1- Not at all	2	3	4 - Somewhat	5	6	7 - Completely
○	○	○	○	○	○	○

How much did the auditory aspects of the environment involve you?

*

Please choose the appropriate response for each item:

1- Not at all	2	3	4 - Somewhat	5	6	7 - Completely
○	○	○	○	○	○	○

How well could you identify sounds?

*

Please choose the appropriate response for each item:

1- Not at all	2	3	4 - Somewhat	5	6	7 - Completely
○	○	○	○	○	○	○

How well could you localize sounds?

*

Please choose the appropriate response for each item:

1- Not at all	2	3	4 - Somewhat	5	6	7 - Completely
◯	◯	◯	◯	◯	◯	◯

How completely were your senses engaged in this experience?

*

Please choose the appropriate response for each item:

1- Not engaged	2	3	4 - Mildly engaged	5	6	7 - Completely engaged
◯	◯	◯	◯	◯	◯	◯

Were there moments during the mixed environment experience when you felt completely focused on the task or environment?

*

Please choose the appropriate response for each item:

1- Not at all	2	3	4 - Sometimes	5	6	7 - All the time
◯	◯	◯	◯	◯	◯	◯

How easily did you adjust to the control devices used to interact with the mixed environment?

*

Please choose the appropriate response for each item:

1- Not at all	2	3	4 - Somewhat difficult	5	6	7 - Very easily
◯	◯	◯	◯	◯	◯	◯

Was the information provided through different senses in the mixed environment (e.g., vision, hearing, touch) consistent?

*

Please choose the appropriate response for each item:

1- Not consistent	2	3	4 - Moderately consistent	5	6	7 - Very consistent
◯	◯	◯	◯	◯	◯	◯

To what extent did you feel completely surrounded by and enveloped by the mixed environment?

*

Please choose the appropriate response for each item:

1- Not at all	2	3	4 - Somewhat	5	6	7 - Completely
◯	◯	◯	◯	◯	◯	◯

As you moved through the mixed environment and interacted with it, did you feel like you were inside the mixed environment, affecting or being affected by objects and events in that environment?

*

Please choose the appropriate response for each item:

1- Not at all	2	3	4 - Moderately affected	5	6	7 - Very affected
◯	◯	◯	◯	◯	◯	◯

How much did your experience in the mixed environment seem like you were in a real place, able to directly sense and interact with the environment?

*

Please choose the appropriate response for each item:

1- Not at all	2	3	4 - Somewhat	5	6	7 - Completely
◯	◯	◯	◯	◯	◯	◯

How physically fit do you feel at the present time?

*

Please choose the appropriate response for each item:

1- Not fit	2	3	4 - Moderately fit	5	6	7 - Extremely fit
◯	◯	◯	◯	◯	◯	◯

How mentally alert do you feel at the present time?

*

Please choose the appropriate response for each item:

1- Not alert	2	3	4 - Moderately	5	6	7 - Fully alert
◯	◯	◯	◯	◯	◯	◯

OQ

If it is more convenient for you, answer in Dutch.

How effective was the training with the 3D avatar?

*

Please choose the appropriate response for each item:

	1- Not at all effective	2	3	4	5	6	7 - Very effective
0	◯	◯	◯	◯	◯	◯	◯

Please, argument your answer to the previous question.

*

Please write your answer here:

How similar was it to the experience of practising with your colleague?

*

Please choose the appropriate response for each item:

	1- Not at all similar	2	3	4	5	6	7 - Extremely similar
3D	◯	◯	◯	◯	◯	◯	◯

Please, argument your answer to the previous question.

*

Please write your answer here:

Did your playing improve in comparison with the last session? Please, argument your answer.

Please write your answer here:

Are there any functions and/or features you would like to add to the VR application that would improve your training/playing more? Please give concrete examples.

Please write your answer here:

In which view mode was it best for you to practice? Please, explain.

*

Please choose **only one** of the following:

- Condition with 3D avatar
- Condition with the beamer

Make a comment on your choice here:

Practicing at home

Did you feel well prepared for the first session?

Please write your answer(s) here:

|Not at all|Very well

Did you find the first excerpt (Dvorak) difficult?

Please write your answer(s) here:

|Not at all|Very difficult

Did you find the second excerpt difficult?

Please write your answer(s) here:

|Not at all|Very difficult

Did you practice orchestra excerpts last week at home?

*

Please choose **only one** of the following:

- Yes
- No

How often did you practice them?

*

Please choose **only one** of the following:

- ◯ 1 time
- ◯ 2 times
- ◯ 3 times
- ◯ 4 - 5 times
- ◯ 6 times
- ◯ Everyday
- ◯ Other

Make a comment on your choice here:

On average, how long was your practice session?

*

Please choose **only one** of the following:

- Less than 30 min
- 30 - 60 min
- 60 - 90 min
- More than 90 min
- Other

Make a comment on your choice here:

Thank you for your participation!

Aleksandra Michalko & Adriaan Campo

If you have more questions about the experiment, do not hesitate to contact us!

aleksandra.michalko@ugent.be adriaan.campo@ugent.be

Submit your survey.
Thank you for completing this survey.
